# Supplementary material for: Large electronegativity differences between adjacent atomic sites activate and stabilize ZnIn2S4 for efficient photocatalytic overall water splitting
Source: Nat Commun. 2024 Jan 6;15:337. doi: 10.1038/s41467-024-44725-1 (PMC10771526; doi:10.1038/s41467-024-44725-1)
Supplement: Supplementary file 1 — Supplementary Information [file 41467_2024_44725_MOESM1_ESM.pdf]

Supplementary Information

Large electronegativity differences between adjacent atomic sites activate and stabilize $ZnIn_2S_4$ for efficient photocatalytic overall water splitting

Xu Xin[1,2], Yuke Li[3], Youzi Zhang[1,2], Yijin Wang[1,2], Xiao Chi[4], Yanping Wei[5], Caozheng Diao[6], Jie Su[7], Ruiling Wang[1,2], Peng Guo[1,2], Jiakang Yu[1], Jia Zhang[3], Ana Jorge Sobrido[8], Maria-Magdalena Titirici[9], Xuanhua Li[1,2*]

[1] State Key Laboratory of Solidification Processing, Center for Nano Energy Materials, School of Materials Science and Engineering, Northwestern Polytechnical University, Xi'an 710072, China

[2] Research & Development Institute of Northwestern Polytechnical University, Shenzhen 518057, China

[3] Institute of High Performance Computing (IHPC), Agency for Science, Technology and Research (A*STAR), 1 Fusionopolis Way, #16-16 Connexis, Singapore 138632, Republic of Singapore

[4] Department of Physics, National University of Singapore 117576, Singapore

[5] College of Science, Gansu Agricultural University, Lanzhou 730070, China

[6] Singapore Synchrotron Light Source, National University of Singapore, 5 Research Link, 117603, Singapore

[7] College of Microelectronics, Xidian University, Xi'an 710072, China

[8] School of Engineering and Materials Science, Faculty of Science and Engineering, Queen Mary University of London, Mile End Road, London E1 4NS, UK

[9] Department of Chemical Engineering, Imperial College London, South Kensington Campus, London SW7 2AZ, United Kingdom

[*]Corresponding author E-mail: lixh32@nwpu.edu.cn

Contents

Supporting Texts......4

Materials......4

Synthesis of photocatalysts......4

Characterization of the photocatalysts......6

X-Ray absorption fine structure......7

Photoelectrochemical measurements......7

Photocatalytic hydrogen evolution and oxygen evolution half-reaction tests......8

Water formation reaction......9

Apparent quantum yield (AQY) calculation methods......9

Consistency analysis of AQY and STH......10

^{18}O isotope-labeled measurement......10

Calculation of carrier transport activation energy (CTAE)......10

Calculation of internal electrical field intensity......11

Transient surface photovoltage (TPV) measurement......11

Calculation of charge separation efficiency......12

Calculation of carrier density and the width of space charge region......12

Density functional theory (DFT) calculations......13

Dipole moment calculations......15

Supplementary Fig. 1. O doped in S site of ZIS......16

Supplementary Fig. 2. Morphologies and structures of photocatalysts......18

Supplementary Fig. 3. XPS, Raman, and ESR characterization of photocatalysts......19

Supplementary Fig. 4. XANES characterization of photocatalysts......21

Supplementary Fig. 5. EXAFS characterization of photocatalysts......22

Supplementary Fig. 6. Structural transition models and formation energies at different O doped concentrations......24

Supplementary Fig. 7. The band gap calculation of photocatalysts......25

Supplementary Fig. 8. Morphology and structural analysis of D-O-ZIS modified Pt and CoO_x photocatalyst......26

Supplementary Fig. 9. Photocatalytic overall water splitting performance of D-O-ZIS with cocatalysts loading......28

Supplementary Fig. 10. Morphology and photocatalytic performance of D-O-ZIS with cocatalysts loading......30

Supplementary Fig. 11. DFT calculations of hydrogen-oxygen recombination......32

Supplementary Fig. 12. Hydrogen-oxygen recombination reactions on D-O-ZIS/Pt/CoO_x photocatalyst......33

Supplementary Fig. 13. Hydrogen-oxygen recombination reactions on D-O-ZIS/Pt/CoO_x photocatalyst......34

Supplementary Fig. 14. Photocatalytic overall water splitting performance..36

Supplementary Fig. 15. Photocatalyst for the AQY in overall water splitting performance......................................37

Supplementary Fig. 16. Wavelength-dependent of AQY during photocatalytic overall water-splitting.....................39

Supplementary Fig. 17. Comparison of photocatalytic overall water splitting for the single photocatalysts………..42

Supplementary Fig. 18. Comparison of photocatalytic overall water splitting for the composite photocatalysts…...44

Supplementary Fig. 19. Photocatalytic overall water splitting performance without cocatalysts loading................46

Supplementary Fig. 20. The overall water splitting performance of ZIS and D-ZIS without cocatalysts loading......49

Supplementary Fig. 21. The H_2 or O_2 evolution half reactions of photocatalysts..50

Supplementary Fig. 22. The mass spectrum of oxygen gas evolved for photocatalytic $H_2{}^{18}O$ splitting 51

Supplementary Fig. 23. Structural stability of the D-O-ZIS after 120 h photocatalytic test....................................52

Supplementary Fig. 24. HRTEM images after testing...53

Supplementary Fig. 25. Structural stability of the D-O-ZIS after 120 h photocatalytic test.....................................54

Supplementary Fig. 26. The universality of overall water-splitting performance for metal sulfides photocatalysts..55

Supplementary Fig. 27. The optical properties for photocatalysts..57

Supplementary Fig. 28. The kinetics of charge transport for photocatalysts..58

Supplementary Fig. 29. The internal electric field intensity determination..59

Supplementary Fig. 30. The internal electric field intensity determination..60

Supplementary Fig. 31. The kinetics of charge separation for photocatalysts..61

Supplementary Fig. 32. The calculated average potential..62

Supplementary Fig. 33. Internal electric field determinated by DFT...63

Supplementary Fig. 34. O 1s XPS spectra of D-O-ZIS before and after 120 h photocatalytic test.............................63

Supplementary Fig. 35. Local structure activation determinated by DFT..64

Supplementary Fig. 36. PDOS of S 3*p* bands in ZIS, D-ZIS, and D-O-ZIS for the intermediates.............................65

Supplementary Fig. 37. DFT adsorption models of ZIS in the photocatalytic HER process.....................................65

Supplementary Fig. 38. DFT adsorption models of D-ZIS in the photocatalytic HER process.................................66

Supplementary Fig. 39. DFT adsorption models of D-O-ZIS in the photocatalytic HER process.............................66

Supplementary Fig. 40. DFT calculated for the photocatalytic OER process on D-O-ZIS...67

Supplementary Fig. 41. DFT calculated for the photocatalytic OER process on Zn site of D-O-ZIS.........................68

Supplementary Fig. 42. DFT calculations on structures with varying Zn vacancy levels and O doping.....................69

Supplementary Fig. 43. The adsorption behavior of catalytic sites on photocatalysts..71

Supplementary Fig. 44. Schematic illustration of redox potentials of sulfur ions and band structure for samples......72

Supplementary Fig. 45. Photocatalytic water splitting mechanisms of ZIS and D-ZIS..73

Supplementary Table 1. Zn 2*p* XPS fitting data of ZIS, D-ZIS, and D-O-ZIS....20
Supplementary Table 2. ICP elemental analysis of ZIS, D-ZIS, and D-O-ZIS....20
Supplementary Table 3. Structural parameters of Zn *K*-edge EXAFS fitting for ZIS, D-ZIS, and D-O-ZIS....23
Supplementary Table 4. The overall water splitting performance of D-O-ZIS with different masses....37
Supplementary Table 5. Calculated AQY values of D-O-ZIS/Pt/CoO_x....38
Supplementary Table 6. Calculated AQY values of ZIS/Pt/CoO_x....39
Supplementary Table 7. Calculated AQY values of D-ZIS/Pt/CoO_x....40
Supplementary Table 8. Calculated STH values of D-O-ZIS/Pt/CoO_x for photocatalytic overall water splitting....41
Supplementary Table 9. Comparation of photocatalytic overall water splitting in reported single photocatalysts....43
Supplementary Table 10. Comparation of photocatalytic overall water splitting for composite photocatalysts....45
Supplementary Table 11. Calculated AQY values of single D-O-ZIS without cocatalysts loading....47
Supplementary Table 12. Calculated STH values of single D-O-ZIS without cocatalysts loading....48
Supplementary References....74

Supporting Texts

Materials

Zinc chloride ($ZnCl_2$, ≥99.0%), indium nitrate ($In(NO_3)_3$, ≥99.0%), thioacetamide (C_2H_5NS, ≥99.0), ethanol (C_2H_5OH, ≥99.7%), sodium iodate ($NaIO_3$, ≥99.8%), sodium sulfate (Na_2SO_4, ≥99.0%), sodium molybdate (Na_2MoO_4, ≥99.0%), poly(vinylidene fluoride) (PVDF, ≥99.0%), N-methyl pyrrolidone (NMP, ≥99.0%) were purchased from Sinopharm Chemical. Heavy-oxygen water ($H_2{}^{18}O$, 99%) was purchased from 3A Chemical.

Synthesis of photocatalysts

Synthesis of distortion-evoked cation-site oxygen doping of MoS_2 (D-O-MoS_2). Firstly, MoS_2 nanoflower was prepared by a hydrothermal method. 1 mmol of Na_2MoO_4, and 2 mmol of thioacetamide were dissolved in 35 mL of deionized water and stirred vigorously for 30 minutes. The mixed solution was then transferred to a 50 mL Teflon-lined autoclave and heated at 200 °C for 20 hours. The precipitates were collected by centrifugation and washed with pure water and ethanol and

then dried at 60 °C in vacuum overnight. Then we used a cathodic electrolysis to introduce distortion states to form distorted MoS_2 (D-MoS_2). The as-made MoS_2 powder was mixed with poly(vinylidene fluoride) with a weight ratio of 7:1, and dispersed in N-methyl pyrrolidone (NMP) under ultrasound to form a black suspension. Next, a lithium-ion battery device, using the MoS_2 as the cathode, a lithium foil as the anode to charge and discharge, the D-MoS_2 cathode was dissembled. The D-MoS_2 catalyst was then purified and obtained. Then, the resulting D-MoS_2 was treated with Ar/O_2 (5%) flow at 500 °C for 10 minutes to introduce O doping. The resulting powder (D-O-MoS_2) was then collected and washed three times with ethanol and deionized water, respectively, before being dried under vacuum at 60 °C.

Synthesis of distortion-evoked cation-site oxygen doping of In_2S_3 (D-O-In_2S_3). The synthesis of In_2S_3 followed the method reported elsewhere. 2 mmol of $In(NO_3)_3$ and 2 mmol thioacetamide were dissolved in 35 mL of deionized water and the solution was stirred vigorously for 30 min. The mixed solution was transferred to a 50 mL Teflon cup, sealed in the autoclave, and heated at 90 °C for 12 h. Once cooled down, the obtained orange suspension was collected, washed with deionized water, and dried at 60 °C under vacuum overnight, and denoted as In_2S_3. Starting from In_2S_3, the In_2S_3 with an edge-distorted structure (D-In_2S_3) was synthesized using a thermal migration strategy. The synthesis process involved heating the precursor material at 500 °C for 30 minutes under an atmosphere of Ar/H_2 (1 bar). Once the reaction was completed, the resulting powder product was allowed to cool naturally to room temperature, after which it was collected and washed several times using ethanol and deionized water and dried at 60 °C under vacuum overnight for further use. For the synthetic process of D-O-In_2S_3, the resulting D-In_2S_3 was treated with Ar/O_2 (5%) flow at 500 °C for 10 minutes. The resulting powder was then collected and washed three times with ethanol and deionized water, respectively,

before being dried under vacuum at 60 °C.

Impregnation-photo-deposition method loaded Pt and CoO_x cocatalyst on D-O-ZIS. Typically, 35 mg D-O-ZIS photocatalyst, H_2PtCl_6 (0.5 mg) and $Co(NO_3)_2 \cdot 6H_2O$ (2.1 mg) was injected into the chamber with 50 mL deionized water and the chamber was irradiated under a 300-W Xe lamp (AM1.5G, 100 mW cm^{-2}) for 30 min. After the light irradiation, the obtained photocatalyst was centrifuged and washed by deionized water and then dried at 80 °C, yielding D-O-ZIS/Pt/CoO_x catalyst.

Characterization of the photocatalysts

The photocatalysts were characterized using various techniques. Scanning electron microscopy (SEM) and energy dispersive X-ray spectroscopy (EDX) were performed using an SEM equipment (FEI Helios G4 CX 450) with an EDX spectrometer. The sample morphology was studied using transmission electron microscopy (TEM) equipped with EDX spectroscopy on FEI Talos F200X. Raman spectra were collected on a Renishaw RM1000 laser Raman spectrometer using a 532 nm laser excitation. X-ray diffraction (XRD) using BRUKER D2 PHASER with Cu Kα (λ=1.5406 Å) radiation was used to determine the crystalline structure of the samples. Ultraviolet-visible diffuse reflectance spectra (UV-vis DRS) were recorded using a JASCO V-570 UV-visible/NIR spectrophotometer in diffuse absorption mode with $BaSO_4$ as a white standard reference. The transient-state surface photovoltage (TPV) measurement was conducted using a monochromatic light source, a lock-in amplifier (SR830-DSP) with a light chopper (SR540), a photovoltaic cell, and a computer. Electron Spin Resonance (ESR) Trapping measurements were performed using an electron spin resonance spectrometer (JEOL JES-FA200, 298 K, 9.062 GHz). X-ray photoelectron spectroscopy (XPS) was performed on a Kratos AXIS Ultra DLD spectrometer, with carbon (C 1*s*: 284.6 eV) used as a reference to calibrate the binding energy of the samples. UV photoelectron spectroscopy (UPS) was conducted

using He I excitation (21.2 eV) and recorded with a constant pass energy of 1 eV in the ultrahigh vacuum (UHV) chamber of the XPS instrument. Photoluminescence (PL) spectra were acquired using an FLS980 fluorescence lifetime spectrophotometer with a Xe lamp, and time-resolved photoluminescence (TRPL) spectra were collected using the TRPL decay fluorescence spectrophotometer (FLSP920, Edinburgh) with an excitation wavelength of 375 nm [1]. Elemental analysis was conducted by inductively coupled plasma emission spectrometer (ICP, Vista-MPX, Agilent, USA).

X-Ray absorption fine structure

The X-ray absorption fine structure (XAFS) spectra were collected at room temperature using a Si (111) double-crystal monochromator at the XAFCA beamline of the Singapore Synchrotron Light Source (SSLS) [2]. The storage ring of SSLS was operated at 0.7 GeV, with a maximum current of 200 mA in decay mode, and the data were collected in transmission mode using ionization chambers as detectors. We used the ATHENA program integrated in the Demeter software packages to background-subtracted, normalize, and Fourier transform the XAFS raw data according to standard procedures [2]. To determine the quantitative structural parameters around central atoms, we performed least-squares curve parameter fitting analysis of the Extended X-ray Absorption Fine Structure (EXAFS) data using the ARTEMIS module of the Demeter software packages. All fits were performed in the R space with a *k*-weight of 3.

Photoelectrochemical measurements

The electrochemical and photoelectrochemical tests were conducted using a Chenhua 760 electrochemical workstation and a three-electrode quartz cell [3]. The working electrode was a fluorine-doped tin oxide (FTO) glass coated with photocatalysts, with a 1×1 cm^2 area. A platinum served as the

counter electrode, while an Ag/AgCl electrode acted as the reference electrode. To prepare the working electrode, 4 mg of photocatalysts were added to a solution of 495 μL of water and 495 μL of ethanol (in a 1:1 volume ratio), along with 10 μL of Nafion (Aladdin). The solution was then ultrasonicated for 15 minutes, and 1 mL of the resulting slurry was spin-coated onto the FTO glass [3]. The electrode was dried before photoelectrochemical measurements were taken. For the electrolyte, a 0.5 M aqueous Na_2SO_4 solution was used. Electrochemical impedance spectroscopy (EIS) measurements were performed on the same three-electrode system, with an AC amplitude of 5 mV and a frequency range of 10^{-2} to 10^{5} Hz. The potential (vs. Ag/AgCl) was converted to the reversible hydrogen electrode (RHE) using the Nernst equation (1):

$$E_{RHE} = E + 0.05916\ pH + E_0 \tag{1}$$

where E_{RHE} is the potential vs. RHE, E_0=0.1976 V at 25 °C, and E is the measured potential vs. Ag/AgCl.

Photocatalytic hydrogen evolution and oxygen evolution half-reaction tests

Photocatalytic measurements were conducted in a Pyrex cell with a closed gas circulation and evacuation system (Beijing Perfectlight Technology, Labsolar 6A) [1, 3]. To initiate the photocatalytic H_2 evolution half-reaction, we dispersed 35 mg of photocatalyst powder in 50 mL of aqueous solution containing 0.35 M Na_2S and 0.25 M Na_2SO_3 sacrificial reagents to consume photogenerated holes. We then bubbled the suspension with argon gas to eliminate dissolved oxygen, creating an anaerobic environment within the reactor. We exposed the solution to a 300 W xenon lamp (Beijing Perfectlight Technology Co., Ltd, 300 mW cm^{-2}) with an optical filter (PLS-CUT420, $\lambda > 300$ nm) and maintained the reaction solution temperature at 15 °C using a recirculating cooling water system. The gases produced during thephotocatalytic reaction were analyzed via gas chromatography equipped with a

thermal conductivity detector (TCD) and a 5 Å molecular sieve column. For the photocatalytic O_2 evolution, we followed the same procedure, except that we used a 20 mM, 50 mL $NaIO_3$ aqueous solution as the sacrificial reagent for consuming photogenerated electrons.

Water formation reaction

The H_2 and O_2 recombination reaction was investigated in the closed gas circulation system. The sample was placed at the bottom of the reactor, and then, H_2 and O_2 gases in a stoichiometric ratio of 2:1 was introduced or generated in the system. The reduction in gas content resulting from water formation reaction in the dark was then monitored by gas chromatography at each interval.

Apparent quantum yield (AQY) calculation methods

To produce one H atom from an H^+ ion, one electron is required, while the production of one H_2 molecule requires two electrons through a one-step excitation process. To measure the AQY for H_2 production, we used a 300 W Xe lamp (PLS-SXE300, PerfectLight) equipped with various bandpass filters (λ=400±10, 420±10, 425±10, 450±10, 500±10 and 550±10 nm) to obtain monochromatic light [3, 4]. The number of incident photons was determined with a fiber spectrometer (AvaSpec-ULS2048XL-EVO). The AQY was calculated using the Supplementary Equation (2), (3) [1], and the AQY for the samples are presented in Fig. 3b and Supplementary Fig. 15.

$$\mathrm{N}=\frac{\mathrm{S}\times P\times\lambda\times\mathrm{t}}{h\times c} \tag{2}$$

$$\mathrm{AQY}(\%)=\frac{\text{number of reacted electrons}}{\text{number of incident photons}}\times 100\%$$
$$=\frac{\text{number of evolved hydrogen molecules }(n_{\mathrm{H_2}}\times N_A)\times 2}{\text{number of incident photons}}\times 100\% \tag{3}$$

where P is the intensity of irradiation light, S is the irradiation area, t is the photocatalytic reaction time, and λ is the wavelength of the monochromatic light, $n_{\mathrm{H_2}}$ is the amount of H_2 molecules, N_A is Avogadro's constant, h is the Planck constant, c is the speed of light.

Consistency analysis of AQY and STH

We performed a consistency analysis of the experimentally measured AQY and STH values [3]. Using a one-step excitation process, we integrated the AQY and light intensity $E(\lambda)$ with different wavelengths (λ) spectrum to calculate the STH value. Our calculations predicted a STH value 0.61%, based on the following Supplementary equation (4) [4]:

$$\mathrm{STH} = \int_{300}^{550} \mathrm{AQY} \times \mathrm{E}(\lambda) \tag{4}$$

The calculated STH value is close to the measured STH value, manifesting good consistency in AQY spectrum and STH.

^{18}O isotope-labeled measurement

To illustrate the origin of oxygen production, ^{18}O isotope-labeled photocatalytic overall water-splitting measurements were performed over D-O-ZIS. The tests were conducted on a Bruker 456-GC gas chromatograph coupled to a Scion SQ mass spectrometer equipped with a thermal conductivity detector and flame ionization detector [3]. With $H_2{}^{18}O$ (99%) used for photocatalytic water splitting, the molar content of the evolved $^{18}O_2$ should theoretically be 98.05% due to the adsorption of reactive species on the photocatalyst surface. The O_2 signals with molecular masses m=32 (O_2) and 34 ($^{16\text{-}18}O_2$) were monitored. The O_2 signals calculated a predicted magnitude of the $^{16\text{-}18}O_2$ signal because the only source was the isotope of $^{18}O_2$. The experimental results in Supplementary Fig. 22 show that the detected evolved $^{18}O_2$ is 98%, close to the theoretical value.

Calculation of carrier transport activation energy (CTAE)

The CTAE is calculated according to the following Arrhenius equation (5) [5-7]:

$$\sigma = \frac{\mathrm{A}}{T} \mathrm{e}^{-E_a/\mathrm{k_B}T} \tag{5}$$

where σ is the conductivity and calculated by $\sigma = \frac{L}{R} * S$, R can be obtained from AC impedance in Supplementary Fig. 28, L and S represent the thickness and section area, A is a constant number, E_a is carrier transport activation energy, k_B is the Boltzmann constant, and T is absolute temperature.

Calculation of internal electrical field intensity

The internal electric field intensity is estimated according to the Supplementary equation (6) developed by Kanata et al [5, 6].

$$F_S = \left(-\frac{2V_s\rho}{\varepsilon\varepsilon_0}\right)^{\frac{1}{2}} \quad (6)$$

where, F_S is the internal electric field magnitude, V_s is the surface voltage, ρ is the surface charge density, ε is the low-frequency dielectric constant, and ε_0 is the permittivity of free space. As ε and ε_0 are constants, the internal electric field intensity is determined by the surface voltage (V_s) and the surface charge density (ρ) in Supplementary Fig. 30. Therefore, we could compare the degree of internal electric field intensity of samples according to the $(V_s\rho)^{1/2}$ values, as shown in the main text of Fig. 4c.

Transient surface photovoltage (TPV) measurement

The charge transfer and separation behaviors can be determined by TPV measurement [8, 9]. The Fermi level of an n-type semiconductor is higher than its surface state. The electrons will accumulate on the surface from the bulk until reaching equilibrium at the Fermi level. The negative charges accumulate on the surface and positive charges accumulate in the bulk, displaying a negative surface voltage. An internal electric field is formed towards the surface direction. The electrons in the bulk are driven by the internal electric field, resulting in increased potential energy and the upward bands bending. Thus, the electrons and holes are excited under light irradiation ($h\nu > E_g$) and migrate to the bulk and surface,

respectively, further repelled by the formed internal electric field and band bending. The stronger the TPV signal, the more photogenerated electrons are on the surface, resulting in a more efficient separation of photogenerated charge carriers.

Calculation of charge separation efficiency

The charge separation efficiency (η) is calculated [10]:

$$\eta = J_{\text{sulfite}} / J_{\text{abs}} \tag{7}$$

where J_{abs} is the photocurrent density with absorbed photons converted into current, J_{sulfite} is the photocurrent density:

$$J_{\text{abs}} = \int_a^b f(x)\, d(x) \tag{8}$$

where a is the shortest wavelength of the emitted light from the Xe arc lamp equipped with a 420 nm cutoff filter, b is the wavelength of the absorption edge of photocatalysts, and f(x) is the relationship formula between the light-converted current densities and irradiation wavelength x. According to the photocurrent density, UV-vis absorption spectra, and spectra irradiation intensity of the Xe arc lamp, the charge separation efficiency of ZIS, D-ZIS, and D-O-ZIS is 3.6%, 11.3%, 39.6%, respectively (see Supplementary Fig. 31).

Calculation of carrier density and the width of space charge region

The carrier density of samples was calculated [11]:

$$N_d = (2/e\varepsilon\varepsilon_0)\left[d\left(1/C^2\right)/dV\right]^{-1} \tag{9}$$

where e is the electron charge, ε and ε_0 are the dielectric constant of the photoanode, and the electric permittivity of vacuum, respectively, and the $d\left(1/C^2\right) / dV$ is the slop by the Mott–Schottky (M-S) plot in Supplementary Fig. 7b.

The width of space charge region, as shown in the main text of Fig. 4d, can be calculated:

$$W = \left((2\varepsilon\varepsilon_0 V_{bi})/(qN_d)\right)^{1/2} \tag{10}$$

where ε and ε_0 are the dielectric constant and the electric permittivity of vacuum, respectively, V_{bi} is the flat-band potential obtained by the M–S plot, and q is the elementary charge.

Density functional theory (DFT) calculations

The DFT based on the generalized-gradient approximation with the exchange-correlation functional of Perdew-Burke-Ernzerhof was performed to proceed with all calculations and the dispersion interaction corrected by the D3 scheme was considered [3]. All the calculations are implemented on the Vienna Ab Initio Simulation Package. The calculation uses reciprocal space on the projector-augmented wave method and a plane-wave basis [12, 13]. An energy cutoff was set to be 500 eV and the Brillouin zone was represented by a 2 × 2 × 1 Gamma-centered mesh of k-point mesh for geometry optimizations. The slab model used a 3 × 3 × 1 supercell for $ZnIn_2S_4$ (001). A vacuum of 15 Å was set on the slabs to separate the interaction between periodic images. The Monkhorst-Pack method with 9 × 9 × 1 was employed for the Brillouin zone sampling for Density of states (DOS) calculations. The formation energy (E_f) of the system from conventional total energy is calculated according to the definition [13]:

$$E_f = E_{tot} - \sum_x E_{tot}(x) \tag{11}$$

where E_f represents the energy required to dissociate the material into its individual components (x).

The H* adsorption free energy (ΔG_{H*}) as a descriptor of hydrogen evolution reaction (HER) activity can be acquired by correcting the adsorption energy of H^*. Gibbs free energy change in 298 K is calculated [14, 15]:

$$\Delta G_{H*} = \Delta E(H^*) + \Delta ZPE - T\Delta S \tag{12}$$

where $\Delta E(H^*)$ is the binding of H^* on the surface. ΔZPE is the zero point energy difference

between absorbed and the gas phase, and $T\Delta S$ is the entropy change of H^*. $T\Delta S$ is considered as half of $TS(H_2)$. $S(H_2)$ is the entropy of H_2 in the gas phase at standard condition. The binding energy is defined as:

$$\Delta E(H^*) = E(H^*) - E(*) - 1/2\ E(H_2) \quad (13)$$

where $E(H^*)$, $E(*)$, and $E(H_2)$ are the energies of H adsorbed to the surface, the energy of the catalyst surface, and the energy of diatomic hydrogen in the gas phase.

The oxygen evolution reaction OER process is a four-electron reaction pathway, which is described by [4]:

$$H_2O + * \rightarrow OH^* + H^+ + e^- \quad (14)$$

$$OH^* \rightarrow O^* + H^+ + e^- \quad (15)$$

$$H_2O + O^* \rightarrow OOH^* + H^+ + e^- \quad (16)$$

$$OOH^* \rightarrow * + O_2 + H^+ + e^- \quad (17)$$

where the symbol * describes an active site on the surface of a catalyst, OH^*, O^* and OOH^* show the adsorbed intermediates during oxygen evolution process.

The Gibbs free energy ΔG for each step can be calculated by [4, 16]:

$$\Delta G = \Delta E + \Delta E_{ZPE} - T\Delta S + \Delta G_U \quad (18)$$

where ΔE is the total energy difference between reactant and product. ΔE_{ZPE} and $T\Delta S$ are the changes in the zero-point energy and the entropic contribution, respectively. The temperature T=298.15 K, and ΔS is the change of entropy. ΔG_U = eU, and the U is the potential vs. standard hydrogen electrode. The zero-point energies for intermediates was calculated through the vibrational frequencies on the adsorbed species including OH, O, and OOH, respectively, for OH^*, O^*, and OOH^*. The over potential (η) for OER process is determined [4, 17]:

$$\eta = \frac{\max\{\Delta G_1, \Delta G_2, \Delta G_3, \Delta G_4,\}}{e} - 1.23V \tag{19}$$

where ΔG_1, ΔG_2, ΔG_3, and ΔG_4 are the Gibbs free energy difference respectively for reaction (14)-(17), when U=0.

Dipole moment calculations

The dipole moment of single relaxed primitive unit cell is carried out by Gaussian09 code. The HF/Lanl2dz is used as the basis sets and the value of the dipole moment is 3.5 D. The dipolar potential field was calculated with the following formula:

$$\widehat{H} = \sum_{\text{dipole, dipole}}^{\text{n, m}} \frac{1}{4\pi\varepsilon_0\varepsilon_r}\left(\frac{p_i p_j}{r^3} - \frac{3(\hat{n}p_i)(\hat{n}p_j)}{r^3}\right) \tag{20}$$

where P_i represents the dipole moment, $\hat{n}$ is the unit vector along the vector r between the dipoles, ε_0 is the dielectric constant of vacuum, and ε_r is the relative dielectric constant. For hexagonal ZIS, the structural parameters are based on the reference (JCPDS No. 03-065-2023), i.e., a = 11.550 Å, b = 11.550 Å, and c = 36.6073 Å, and $\alpha = \beta = 90$ °C, $\gamma = 120$ °C. The Zn-S bond length is estimated to be 2.28 Å for ZIS. When the distortion introduced, the dipole moments change much on D-ZIS and D-O-ZIS along with the changed bond lengths on outer surface, producing the dipolar potential field in samples. The calculated dipolar potential fields for ZIS, D-ZIS, and D-O-ZIS are 0.02×10^{-25}, 2.13×10^{-21}, 7.89×10^{-21} V m^{-1}, respectively.

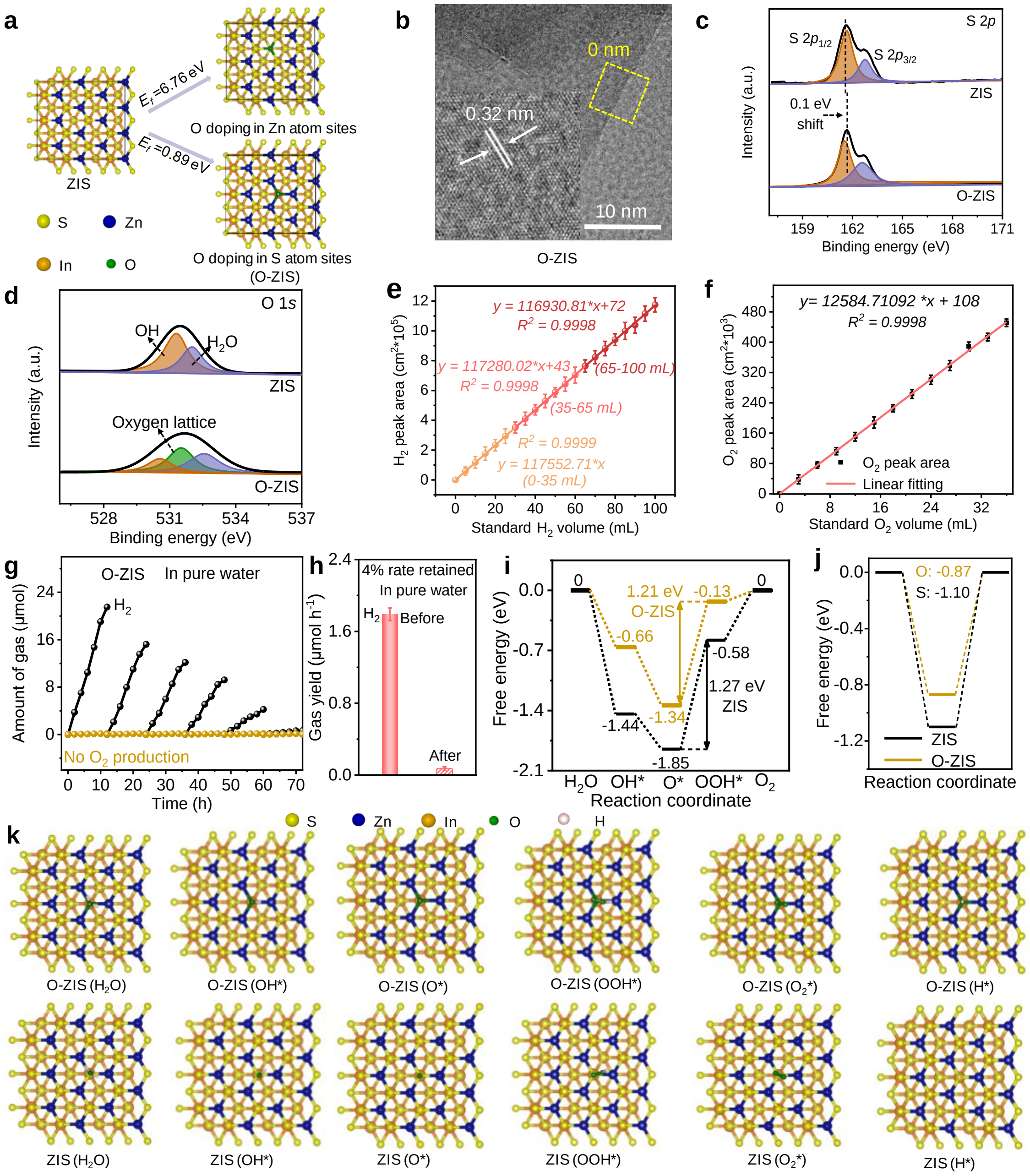

Supplementary Fig. 1. **O doped in S site of ZIS. a** The calculated energy input required for O doping in S atom sites of ZIS (O-ZIS) and O doping in Zn atom sites of ZIS. The structures are in top view; **b** TEM image of O-ZIS; **c** XPS spectra of S 2*p* in ZIS and O-ZIS; **d** XPS spectra of O 1*s* in ZIS and O-ZIS; **e** The standard curve for H_2 testing of peak area obtained from the chromatographic column for each standard concentration of H_2 (Volume range: 0-35 mL, 35-65 mL, 65-100 mL). The standard curves for the volumes in these three intervals approximate a straight line. Error bars represent the standard deviations from the statistic results of three sets of experiments; **f** The standard curve for O_2 testing of peak area obtained from the chromatographic column for each standard concentration of O_2 (Volume range: 0-36 mL). Error bars represent the standard deviations from the statistic results of three sets of experiments; **g** Time-dependent photocatalytic overall water splitting over O-ZIS. Reaction conditions: under standard AM1.5G illumination (100 mW cm^{-2}), the photocatalyst mass is 35 mg and the photocatalytic activity is evaluated via the total hydrogen and oxygen yield of a cycle, the time of each cycle is 12 h. **h** Photocatalytic gas evolution rate of O-ZIS before and after photocatalytic overall water splitting test in pure water. Error bars represent the standard deviations

from the statistic results of three sets of experiments; **i** DFT calculated free energy profile of OER process on O site of O-ZIS and pristine ZIS at pH=0 and U=1.23 V vs. SHE (where * represents the intermediate state); **j** The computed values of $\triangle G_{H^*}$ on ZIS, and O-ZIS; **k** The corresponding DFT models of H_2O, OH^*, O^*, OOH^*, and O_2 intermediates adsorption and H^* adsorption on O-ZIS and ZIS.

We investigate the effects of O doping in S atom sites of ZIS (O-ZIS) on its photocatalytic properties. Firstly, we performed DFT calculations to gain insight into the energy input required for O doping at different sites (Supplementary Fig. 1a). DFT calculations showed that O doping in S atom sites had an energy input of 0.89 eV, while doping O atoms into Zn atom sites required a high-energy input of 6.76 eV, making it difficult to take place. Experimentally, we synthesized O-ZIS using a hydrothermal method and examined its structural and electronic properties using HRTEM and XPS tests. HRTEM image confirmed that the O-ZIS sample had a hexagonal phase with an interplanar distance of 0.32 nm corresponding to the (102) lattice plane (Supplementary Fig. 1b) [17-21]. Notably, no distorted edge shell was induced generation on the O-ZIS sample, indicating that conventional O doping in anion-site of S atom sites did not induce distortion generation. XPS analysis revealed a higher binding-energy peak shift for S 2*p* in O-ZIS, which attributed to O doping (Supplementary Fig. 1c). The oxygen lattice peak at 531.8 eV was detected only in the O-ZIS sample (Supplementary Fig. 1d). The standard curve for hydrogen gas was fitted within three volume intervals (0-35, 35-65, 65-100 mL) for hydrogen quantification (Supplementary Fig. 1e). The range of O_2 standard curve was between 0-36 mL for oxygen quantification (Supplementary Fig. 1f). We conducted photocatalytic experiments to assess the effectiveness of O-ZIS for photocatalytic performance. Photocatalytic experiments demonstrated that O-ZIS only produced H_2, with a H_2 evolution amount of 21.5 μmol and no O_2 detected (Supplementary Fig. 1g). O-ZIS retained only ~4% of its original photocatalytic gas evolution rate after 72 h reaction (Supplementary Fig. 1h). We performed DFT calculations to gain insight into the impact of O doping on hydrogen and oxygen adsorption/desorption. O-ZIS and ZIS showed high free energy barriers of 1.21 and 1.27 eV for O^* adsorbed during the oxygen evolution process, respectively, indicating that O doping in S atom sites of ZIS had little improvement on oxygen evolution (Supplementary Fig. 1i). O-ZIS had an optimum hydrogen adsorption-free energy ($\triangle G_{H^*}$) of -0.87 eV at the doped O site and -1.10 eV at ZIS (Supplementary Fig. 1j), indicating that O doping in S atom sites improved hydrogen species adsorption/desorption on O-ZIS. The adsorption structures of O-ZIS and ZIS is shown in Supplementary Fig. 1k.

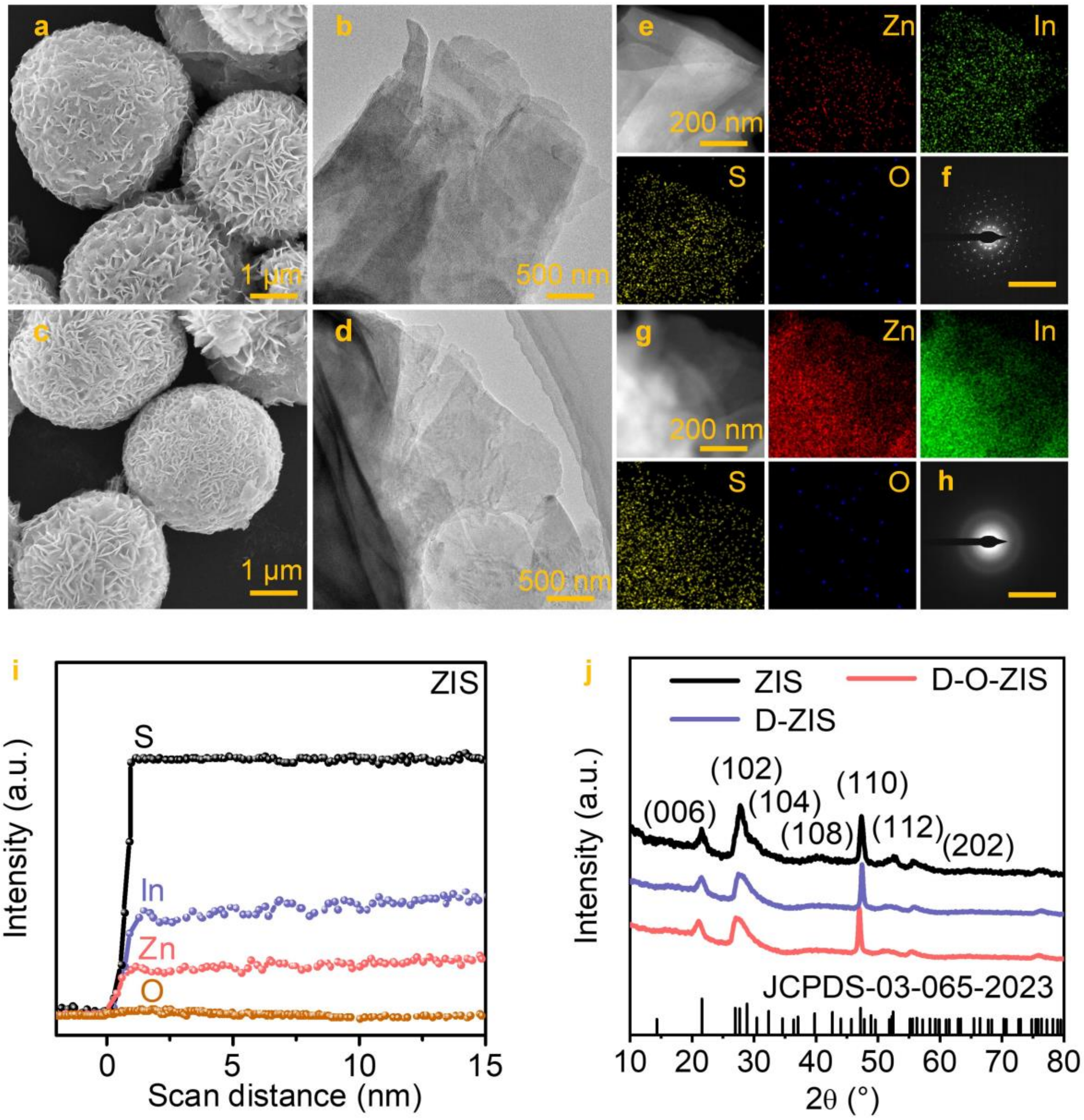

Supplementary Fig. 2. **Morphologies and structures of photocatalysts. a** SEM image of ZIS; **b** TEM image of ZIS; **c** SEM image of D-ZIS; **d** TEM image of D-ZIS; **e** The energy-dispersive X-ray spectroscopy (EDX) elemental mapping images of ZIS; **f** SAED pattern of ZIS. The scale bar is 5 nm^{-1}; **g** The corresponding EDX mapping images of D-ZIS; **h** SAED pattern of D-ZIS. The scale bar is 5 nm^{-1}; **i** The element distribution and HAADF-STEM line scans of Zn, In, S and O elements of the ZIS from the outer edge to the core; **j** XRD patterns of ZIS, D-ZIS and D-O-ZIS.

The EDX mapping images revealed that Zn, In, and S elements were uniformly dispersed in both ZIS and D-ZIS, while O element dispersion was absent in both (Supplementary Fig. 2e, g). The ZIS structure displayed a crystalline phase in its SAED pattern (Supplementary Fig. 2f), while the D-ZIS structure showed a distorted structure (Supplementary Fig. 2h).

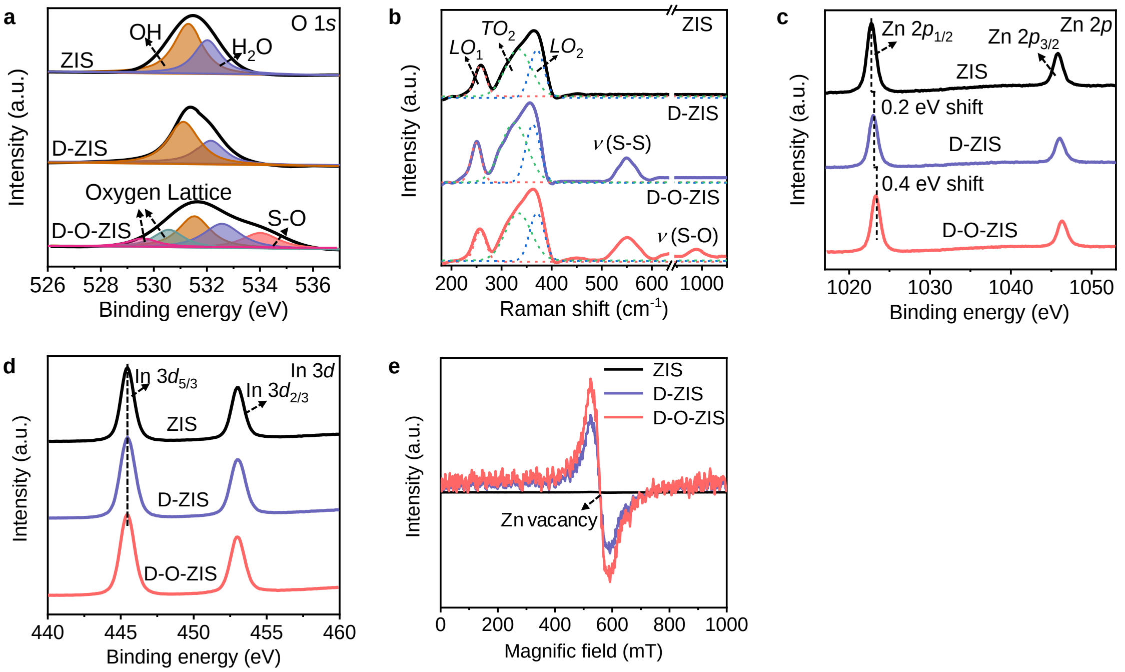

Supplementary Fig. 3. **XPS, Raman, and ESR characterization of photocatalysts.** **a** XPS spectra of O 1*s* in ZIS, D-ZIS and D-O-ZIS; **b** Raman spectra and the peak fitting of ZIS, D-ZIS and D-O-ZIS; **c** XPS spectra of Zn 2*p* in ZIS, D-ZIS and D-O-ZIS; **d** XPS spectra of In 3*d* in ZIS, D-ZIS and D-O-ZIS; **e** ESR spectra of ZIS, D-ZIS and D-O-ZIS.

The O 1*s* XPS spectra shows OH and H_2O XPS peaks located at 531.3 and 532.3 eV for ZIS, D-ZIS, and D-O-ZIS, and the lattice O and S-O XPS peaks only for D-O-ZIS (Supplementary Fig. 3a) [18]. In the lattice O peak, the doped oxygen atoms coordinate with both the Zn atoms and the S atoms. Zn-O bonding can be observed at 529.81 eV [19]. The Raman patterns of D-ZIS and D-O-ZIS had ν(S-S) Raman signatures at about 550 cm^{-1} [18]. Additionally, a peak at 990 cm^{-1} for D-O-ZIS was attributed to the S-O stretch (Supplementary Fig. 3b) [18]. The Zn 2*p* peaks of ZIS, D-ZIS, and D-O-ZIS exhibited shifts. The higher binding energy shifts of Zn 2*p* peaks in D-ZIS (0.2 eV shift) and D-O-ZIS (0.4 eV shift) suggested increased electron loss of Zn atoms due to edge distortion or O atom doping incorporation (Supplementary Fig. 3c) [14, 20]. The In 3*d* peaks of ZIS, D-ZIS, and D-O-ZIS showed negligible shifts, indicating an unchanged local structure and electronic structure for In sites in all samples (Supplementary Fig. 3d). The Raman peaks at 242, 318, and 363 cm^{-1} were assigned to the LO_1, TO_2, and LO_2 modes of ZIS, respectively [1, 14, 18]. Meanwhile, we calculated the Zn vacancies from Zn 2*p* XPS a with a concentration of ~2.3% and ~3.7% for D-ZIS and D-O-ZIS, respectively.

Supplementary Table 1. Zn 2*p* XPS fitting data of ZIS, D-ZIS, and D-O-ZIS

Catalysts	Zn $2p_{3/2}$			Zn $2p_{1/2}$		
	Peak (eV)	FWHM	Area	Peak (eV)	FWHM	Area
ZIS	1022.7	1.62	6163.4	1045.6	1.72	4003.9
D-ZIS	1022.9	1.61	5987.9	1045.9	1.70	3946.5
D-O-ZIS	1023.1	1.63	5911.8	1046.2	1.73	3881.2

The Zn content can be approximately calculated based on the XPS peak area of Zn 2p. By comparing the Zn element content of ZIS, D-ZIS, and D-O-ZIS, the approximate amount of Zn loss can be determined. The lost Zn content in D-ZIS is estimated to be 2.3%, whereas in D-O-ZIS, it is approximately 3.7%.

Supplementary Table 2. ICP elemental analysis of ZIS, D-ZIS, and D-O-ZIS

Catalysts	Zn (mg L^{-1})	In (mg L^{-1})	S (mg L^{-1})
ZIS	3.67	12.31	7.75
D-ZIS	3.43	12.32	7.71
D-O-ZIS	3.18	12.31	7.72

Inductively coupled plasma (ICP) emission spectrometer instrument was employed to accurately analyze the amounts of Zn, In and S in ZIS, D-ZIS, and D-O-ZIS. The atomic ratio of Zn, In and S in ZIS, D-ZIS, and D-O-ZIS is 1: 2.05 :3.93, 0.91:2.05:3.92, and 0.86:2.05:3.93, respectively. From the comparison about the atomic proportions of ZIS, D-ZIS, and D-O-ZIS, it can be identified that the Zn vacancies exist in D-ZIS and D-O-ZIS. ICP analysis revealed that the Zn vacancy content in D-ZIS is approximately 1.01 wt%, while in D-O-ZIS, it is approximately 2.06 wt%.

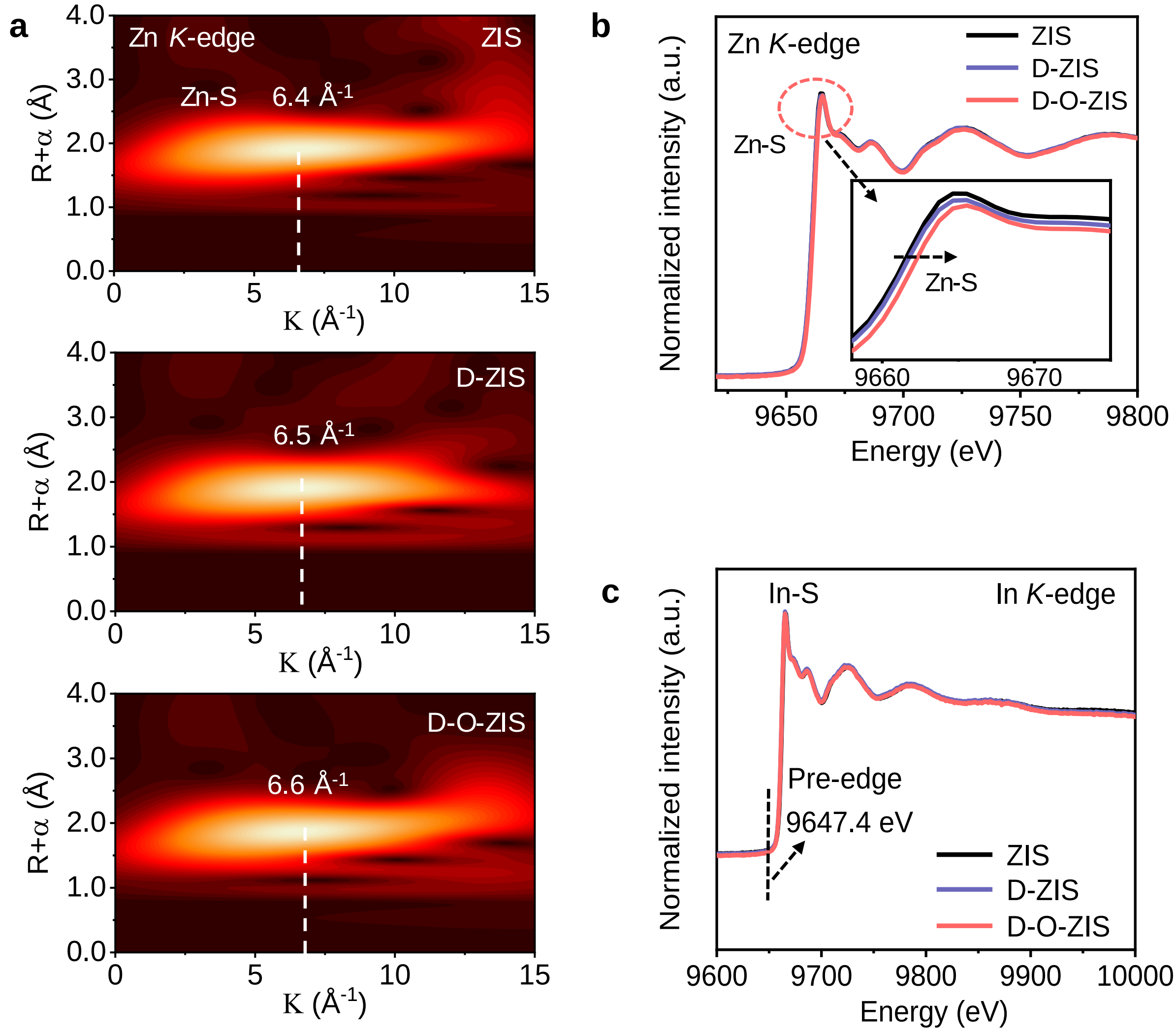

Supplementary Fig. 4. **XANES characterization of photocatalysts.** **a** Wavelet transform of the Zn *K*-edge k^3-weighted EXAFS spectra of ZIS, D-ZIS and D-O-ZIS. (The extracted fitting results in Supplementary Table 3); **b** The XANES spectra of Zn *K*-edge of ZIS, D-ZIS, and D-O-ZIS. Embedded image is an enlargement for the Zn *K*-edge; **c** The XANES spectra of In *K*-edge of ZIS, D-ZIS, and D-O-ZIS.

The ZIS sample showed a strong wavelet contour at 6.4 $Å^{-1}$, which is attributed to Zn-S coordination (Supplementary Fig. 4a). The D-O-ZIS sample, on the other hand, displayed an intensity peak at 6.6 $Å^{-1}$, higher than those for ZIS (6.4 $Å^{-1}$) and D-ZIS (6.5 $Å^{-1}$), due to electron loss in Zn atoms caused by the Zn vacancy. Furthermore, the Zn *K*-edge XANES spectra of D-O-ZIS showed a different edge around 9665.4 eV, with a slightly higher energy shift, resulting from electron loss in Zn atoms due to the Zn vacancy (Supplementary Fig. 4b). In contrast, the In *K*-edge XANES spectra for all samples exhibited a similar pre-edge feature at 9647.4 eV, indicating similar chemical composition and site states (Supplementary Fig. 4c).

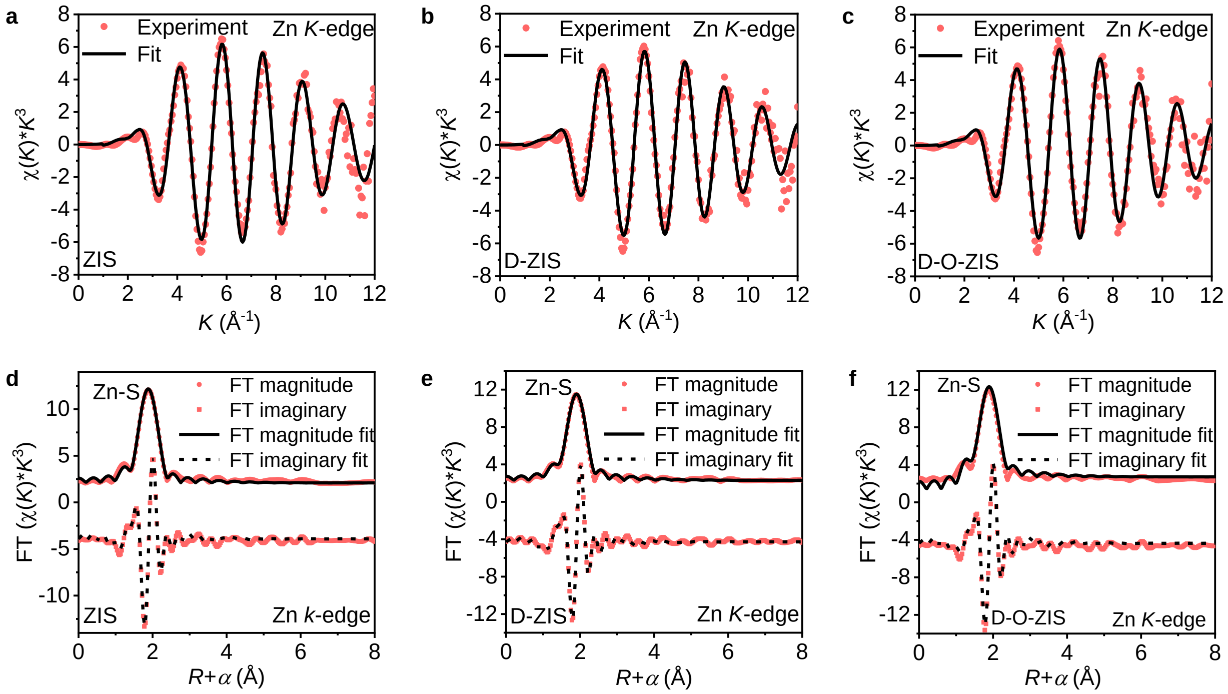

Supplementary Fig. 5. **EXAFS characterization of photocatalysts.** The EXAFS spectra of Zn *K*-edge (points) and the curve fit (line) for **a** ZIS; **b** D-ZIS; and **c** D-O-ZIS; The spectra are shown in k^3-weighted *k*-space. The Fourier-transformed $k^3\chi(k)$ curves of Zn *K*-edge EXAFS (FT-EXAFS) spectra (points) and the curve fit (line) for **d** ZIS, **e** D-ZIS, and **f** D-O-ZIS.

We used the Monte Carlo Method and the Least Square Method to analyze FT-EXAFS fitting results [2], with the coordination number and bonding lengths presented in Supplementary Table 3. Our analysis revealed that the theoretical and experimental spectra had good agreement, indicating that the structure was similar to the actual situation. We obtained the shell of the coordination element through EXAFS fitting, which provided information on coordination number *N*, bond length *R*, system disorder σ^2 (Debye-Waller factor), energy correction $\triangle E^0$, and *R* factor used to determine the quality of fitting. Our DFT calculations produced optimized models, as shown in Fig. 2g, which matched well with the XANES spectra information of the structures. The bond length results in Supplementary Table 3 were also consistent with these findings, demonstrating the accuracy of our calculations. Further analysis using Zn *K*-edge EXAFS fitted results revealed a decrease in the coordination number to 2.8 in the first shell of the Zn-S path and a change in Zn-S bond length to 2.32 Å for D-ZIS, indicating the generation of distortion states. The Zn *K*-edge EXAFS fitting results also showed a reduced coordination number of 2.6 in the Zn-S path for D-O-ZIS, with a Zn-S bond length of 2.33 Å. These results were consistent with the DFT-calculated Zn-S bond lengths of ZIS, D-ZIS, and D-O-ZIS, which were approximately 2.28 Å, 2.32 Å, and 2.33 Å, respectively, and matched well with the Zn *K*-edge

EXAFS fitting of bond length results. In summary, our results confirm the successful determination of the D-O-ZIS structure using both EXAFS results and DFT modeling calculations.

Supplementary Table 3. Structural parameters of Zn *K*-edge EXAFS fitting for ZIS, D-ZIS, and D-O-ZIS.

Sample	Scattering path	N	σ^2	ΔE_0	R (Å)	R-factor
ZIS	Zn-S1	3.0 (0.5)	0.0049 (0.0020)	0.7	2.29 (0.02)	0.0006
D-ZIS	Zn-S1	2.8 (0.6)	0.0107 (0.0030)	1.2	2.32 (0.02)	
D-O-ZIS	Zn-S1	2.6 (0.4)	0.0046 (0.0010)	0.9	2.33 (0.02)	

Note. N: Coordination number, σ^2: Debye Waller factors, R: Bond distance, ΔE_0: The inner potential correction, R factor: Goodness of fit [2].

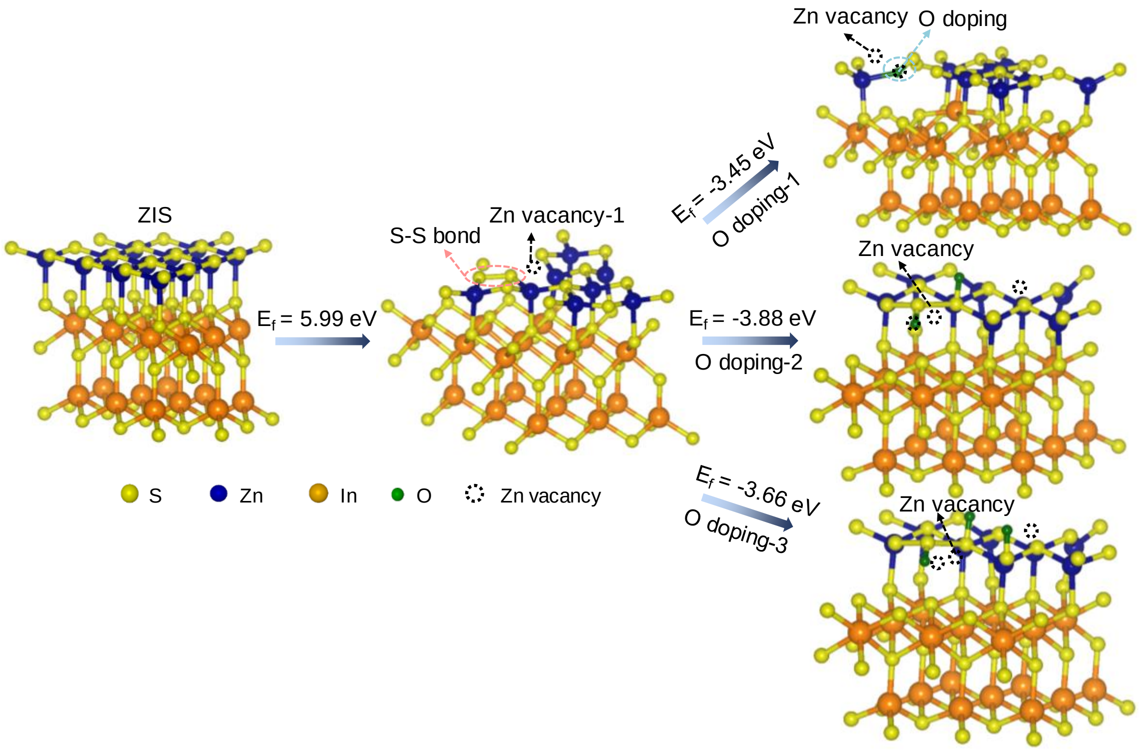

Supplementary Fig. 6. Structural transition models and formation energies at different O doped concentrations.

We utilized DFT calculations to investigate the impact of different O doping concentrations on the structure of D-ZIS as shown in Supplementary Fig. 6. Specifically, we examined the effects of one, two, and three O atoms being doped. Our findings reveal that when one O atom is doped, it has a propensity to occupy the position of a Zn atom in the resulting structure, leading to the creation of a neighboring Zn vacancy. This phenomenon can induce structural distortion. Moreover, the introduction of two or three O atoms further promotes the formation of Zn vacancies, thereby intensifying the degree of structural distortion.

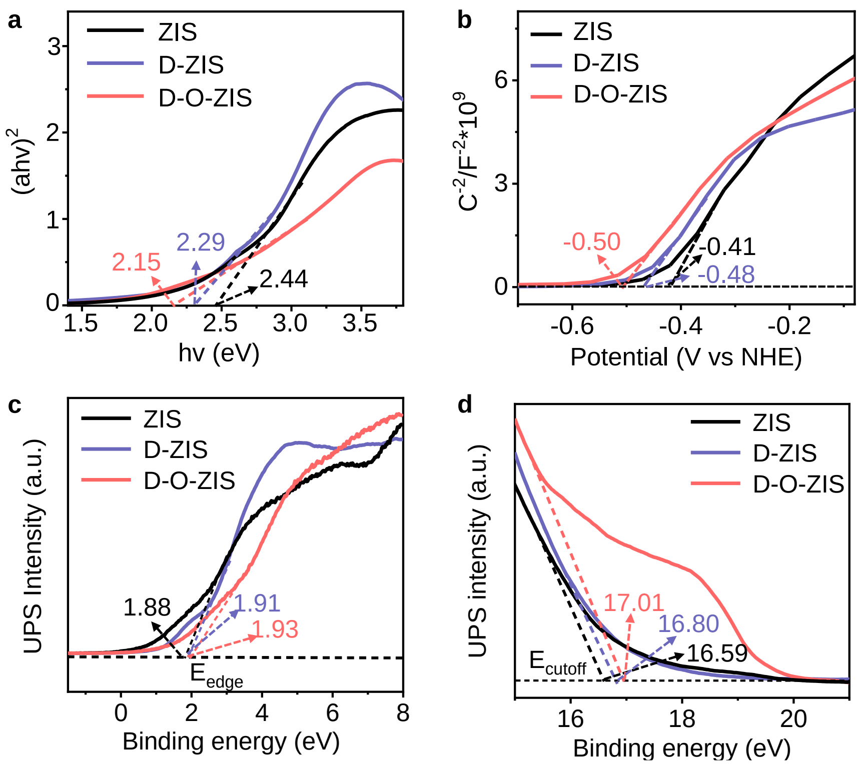

Supplementary Fig. 7. **The band gap calculation of photocatalysts. a** Tauc plots of ZIS, D-ZIS, and D-O-ZIS using Kubelka-Munk function vs. the energy of incident light obtained; **b** Mott-Schottky (M-S) plots of ZIS, D-ZIS, and D-O-ZIS; **c** UPS spectra of the valence band (E_{VBM}) region (E_{edge}); **d** UPS spectra of the cut-off region (E_{cutoff}) for the ZIS, D-ZIS and D-O-ZIS.

We investigated the energy band structures of ZIS, D-ZIS, and D-O-ZIS samples using two methods: M-S plots and UPS spectra. The flat band calculated by M-S plots were close to -0.41, -0.48, and -0.50 eV (vs. NHE), respectively. The estimated valence band (VB) were 2.01, 1.83, and 1.64 eV for ZIS, D-ZIS, and D-O-ZIS, respectively. UPS was used to further determine the ionization potential, which is equivalent to the energy band of VB. The values of work function (φ) for ZIS, D-ZIS, and D-O-ZIS were 4.63, 4.42, and 4.21 eV (vs. vacuum), respectively. The VB of ZIS, D-ZIS, and D-O-ZIS were calculated to be 2.01, 1.83, and 1.64 eV [20-24]. The estimated conduction band (CB) calculated by UPS were close to -0.43, -0.46, and -0.51 eV for ZIS, D-ZIS, and D-O-ZIS, respectively, which is consistent with the M-S results. The Fermi level (E_F) of the samples was aligned at 0.13, -0.08, and -0.29 eV for ZIS, D-ZIS, and D-O-ZIS, respectively.

$$\mathrm{hv} = E_{\mathrm{cutoff}} + \varphi \tag{21}$$

$$E_{\mathrm{VB}} = E_{\mathrm{edge}} + \varphi \tag{22}$$

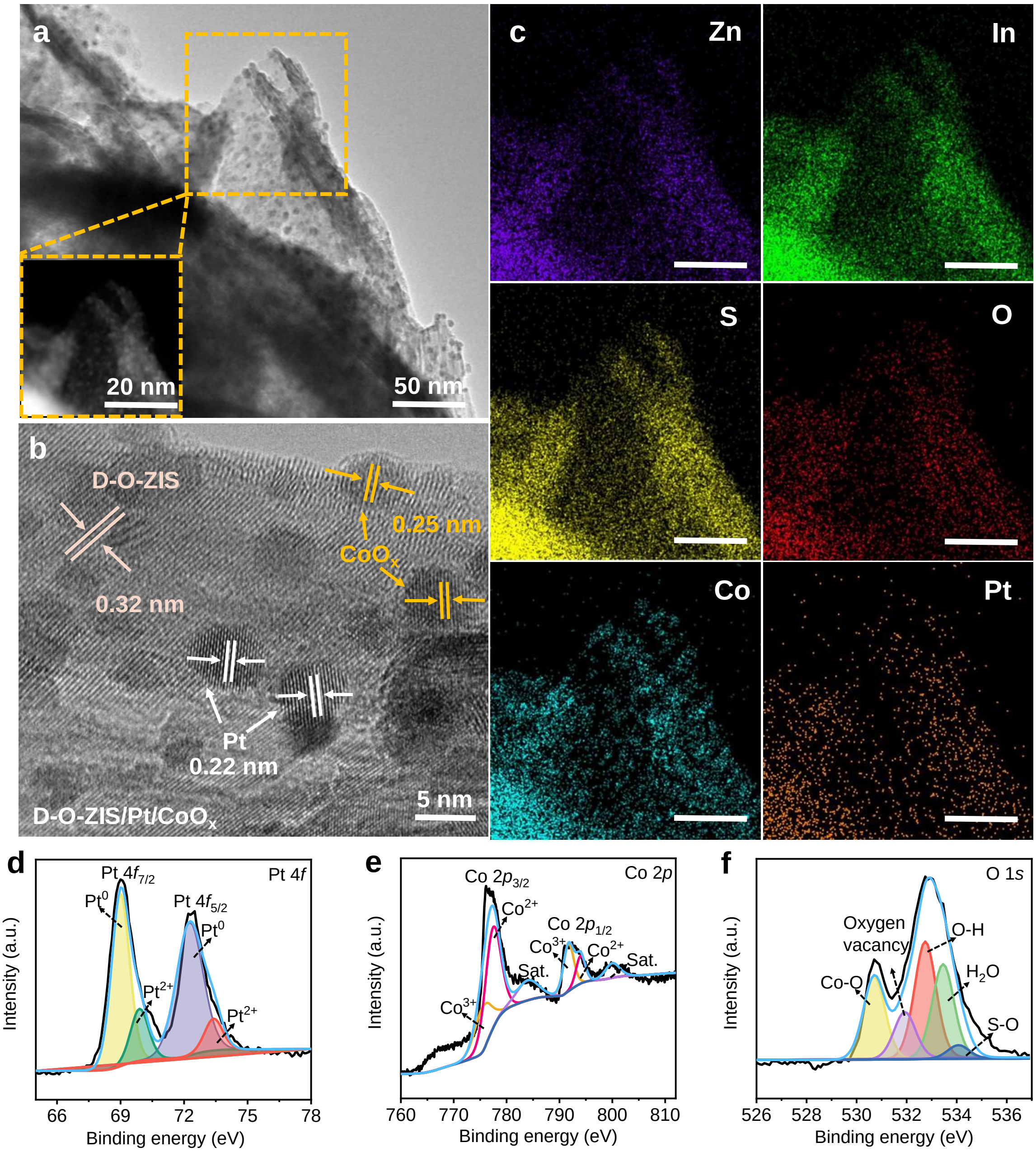

Supplementary Fig. 8. **Morphology and structural analysis of D-O-ZIS modified Pt and CoO_x photocatalyst.** **a** TEM image of D-O-ZIS/Pt/CoO_x at low magnification; **b** HRTEM image of D-O-ZIS/Pt/CoO_x; **c** Scanning TEM image (Embedded in Supplementary Fig. 8a) and the corresponding EDX elemental mapping images of the distribution of Zn, In, S, O, Co, and Pt species on D-O-ZIS/Pt/CoO_x. The scale (20 nm) applies to the images in Supplementary Fig. 8c; **d** XPS spectra of Pt 4*f* in D-O-ZIS/Pt/CoO_x; **e** XPS spectra of Co 2*p* in D-O-ZIS/Pt/CoO_x; **f** XPS spectra of O 1*s* in D-O-ZIS/Pt/CoO_x.

As shown in Supplementary Fig. 8, TEM image of the D-O-ZIS/Pt/CoO_x photocatalyst by the impregnation-photo-deposition method depicts a uniform distribution of Pt nanoparticles and CoO_x nanoparticles on the D-O-ZIS surface (Supplementary Fig. 8a). To further elucidate the interfacial contact between CoO_x and D-O-ZIS, we measured the high-resolution transmission electron

microscopy (HRTEM) image of D-O-ZIS/Pt/CoO_x (Supplementary Fig. 8b). The Pt nanoparticles exhibited diameters of approximately 5-6 nm, and the CoO_x nanoparticles measured around 5 nm on the surface of D-O-ZIS. A small number of large particles appeared in the morphology. The resolved lattice distances of 0.22 nm match well with the (111) plane of Pt, while a lattice distance of 0.25 nm is attributed to the (311) plane of CoO_x. Both Pt and CoO_x are closely connected with the (102) plane of D-O-ZIS measuring 0.32 nm. The energy-dispersive X-ray spectroscopy (EDX) elemental mapping results in Supplementary Fig. 8c further prove the intimate contact between CoO_x and D-O-ZIS [25-30]. As shown in Supplementary Fig. 8d, the XPS of Pt 4*f* spectrum of D-O-ZIS/Pt/CoO_x presents two dominating peaks located at 69.0 and 72.3 eV, which belongs to metallic Pt. The other two peaks at 69.9 and 73.4 eV are attributed to Pt^{2+}, which is probably owing to the constructed Pt single atoms [20]. As shown in Supplementary Fig. 8e, the Co 2*p* spectrum of D-O-ZIS/Pt/CoO_x showed the binding energy of Co^{3+} peak (775.9 and 791.6 eV) and Co^{2+} peak (777.6 and 793.9 eV), indicating the formation of the CoO_x structure [25, 26]. As shown in Supplementary Fig. 8f, the deconvoluted O 1*s* spectrum of D-O-ZIS/Pt/CoO_x exhibits two peaks located at 530.7 and 531.8 eV representing the lattice oxygen (CoO) and oxygen deficiency, respectively. The other three peaks are attributed to the OH vibration, H_2O peak, and S-O bonding peak.

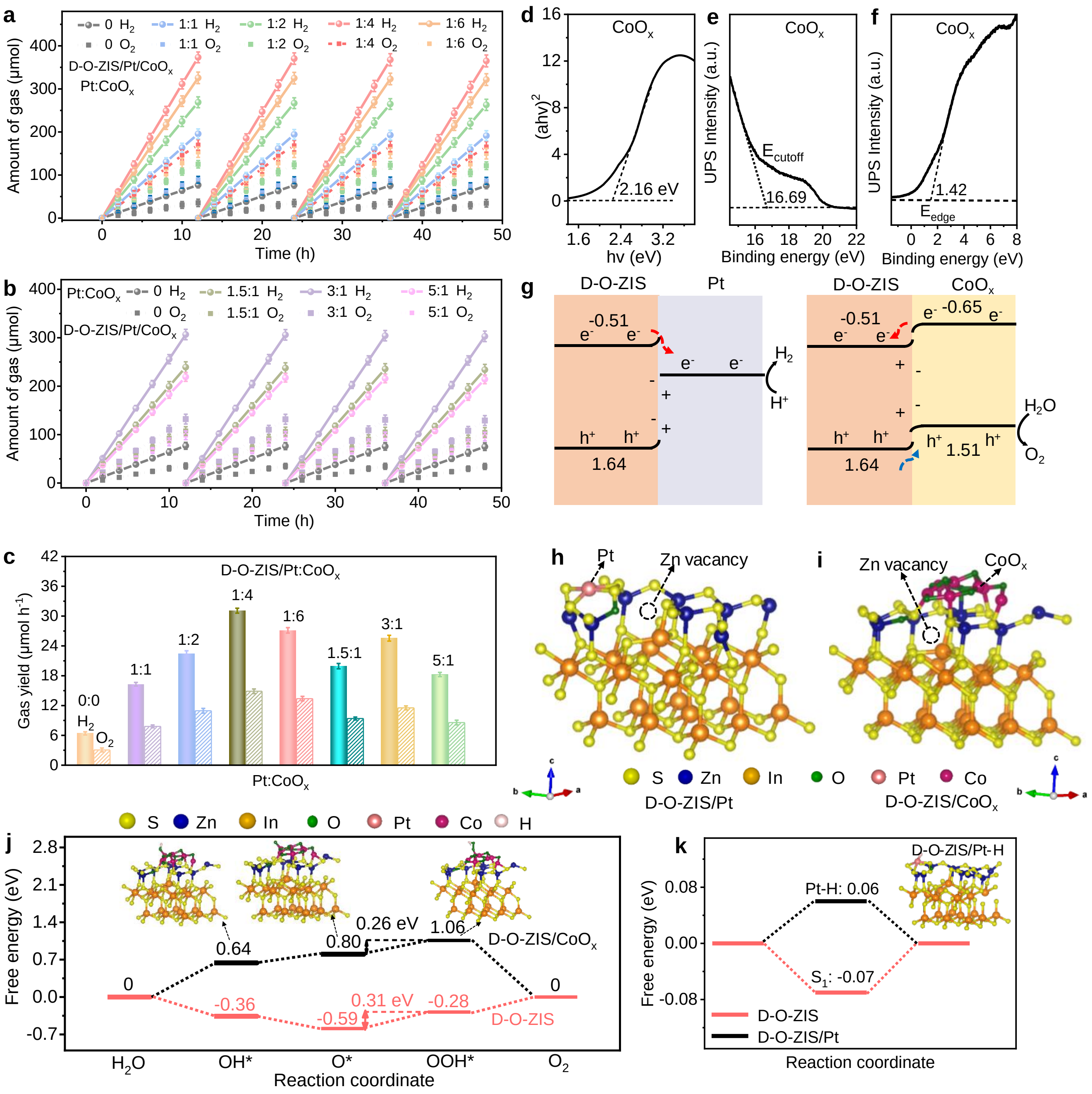

Supplementary Fig. 9. **Photocatalytic overall water splitting performance of D-O-ZIS with cocatalysts (Pt, CoO_x) loading. a, b** Time-dependent photocatalytic overall water splitting over D-O-ZIS and D-O-ZIS/Pt/CoO_x with different Pt:CoO_x ratios in pure water under standard AM 1.5 illumination (100 mW cm^{-2}), the photocatalyst mass was 35 mg and the photocatalytic activity was evaluated via the total hydrogen and oxygen yield of a cycle, the time of each cycle is 12 h. Error bars represent the standard deviations from the statistic results of three sets of experiments; **c** Photocatalytic gas evolution rate of D-O-ZIS and D-O-ZIS/Pt/CoO_x with different Pt:CoO_x ratios for photocatalytic overall water splitting test in pure water; Error bars represent the standard deviations from the statistic results of three sets of experiments; **d** Kubelka-Munk function vs. the energy of incident light plot of CoO_x; **e** UPS spectra of the cut-off region (Secondary cut-off binding energy: E_{cutoff}) for CoO_x; **f** UPS spectrum of the valence band energy region (E_{edge}) of CoO_x; **g** Band diagram for equilibrium in the D-O-ZIS/Pt and D-O-ZIS/CoO_x photocatalysts interface during the photocatalytic water splitting; **h** Visual representation of D-O-ZIS/Pt surface; **i** Visual representation of D-O-ZIS/CoO_x surface; **j** DFT calculated free energy profile of OER process on D-O-ZIS and D-O-ZIS/CoO_x at pH=0

and U=1.23 V vs. SHE (where * represents the intermediate state); **k** The computed values of $\triangle G_{H^*}$ in D-O-ZIS, and D-O-ZIS/Pt. The adsorption structure is embedded in the diagram.

We loaded cocatalysts of Pt and CoO_x on D-O-ZIS to investigate the photocatalytic activity. By optimizing the co-catalyst content, the photocatalytic overall water-splitting performance of D-O-ZIS/Pt/CoO_x was enhanced (Supplementary Fig. 9a, b). When the ratio of Pt to CoO_x is 1:4, the D-O-ZIS/Pt/CoO_x exhibits highest photocatalytic H_2 and O_2 evolution rate with a value of 31.1 and 14.8 μmol h^{-1}, respectively, which is increased by 4.8 times compared to ZIS (Supplementary Fig. 9c). Meanwhile, the matched energy band structures of D-O-ZIS/Pt and D-O-ZIS/CoO_x induced that photoinduced electrons are driven from the CBM of D-O-ZIS to Pt cocatalyst to produce H_2, while photogenerated holes are consumed in the VBM of CoO_x to generate O_2 (Supplementary Fig. 9d-f). DFT calculations were performed to further elucidate the mechanism of the promotion effects of the Pt cocatalyst on the H_2-evolving reaction and CoO_x cocatalyst on the O_2-evolving reaction from the viewpoint of surface catalysis [19, 20]. The Pt cluster and CoO_x cluster were simply extracted and placed on the surface of D-O-ZIS to simulate the D-O-ZIS/Pt and D-O-ZIS/CoO_x interfaces, respectively (Supplementary Fig. 9g, h). The Gibbs free energy change diagram of the four elementary steps of O_2 evolution on the surface of D-O-ZIS and D-O-ZIS/CoO_x was conducted. It was demonstrated that the rate-determining step of D-O-ZIS and D-O-ZIS/CoO_x is the adsorption of one OH^* to form OOH^* from O^*. The decrease of the Gibbs free energy barrier of 0.26 eV was observed for D-O-ZIS/CoO_x compared to D-O-ZIS (0.31 eV), suggesting the functionality of the cocatalyst (Supplementary Fig. 9i). The optimum hydrogen adsorption-free energy ($\triangle G_{H^*}$) was −0.07 eV at the S_1 site and 0.06 eV at the Pt site, indicating that the Pt site is slightly conducive to hydrogen adsorption/desorption than constructed S_1 site in D-O-ZIS, indicating the Pt cocatalyst enhances photocatalytic hydrogen production due to its suitable adsorption (Supplementary Fig. 9j).

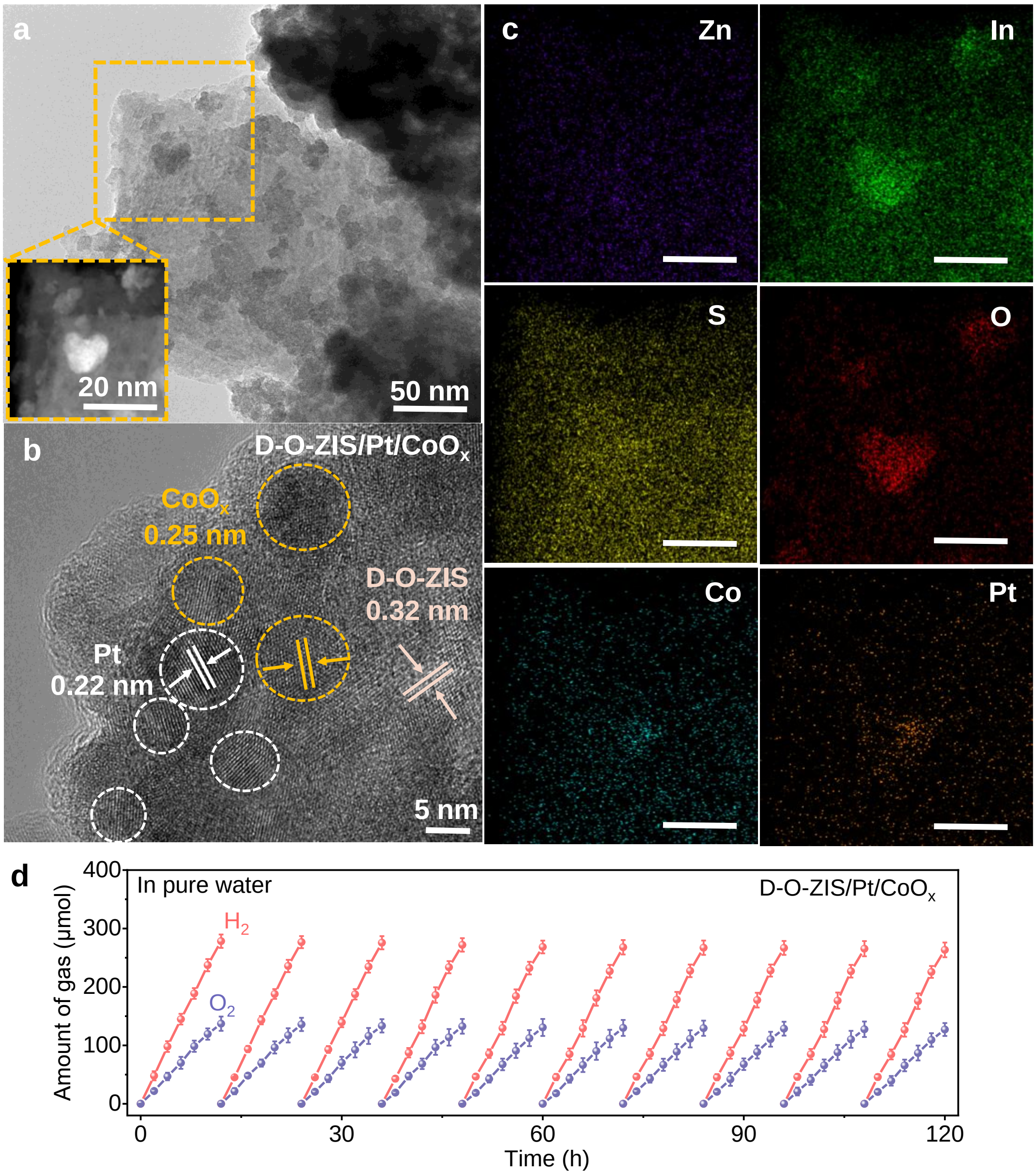

Supplementary Fig. 10. **Morphology and photocatalytic performance of D-O-ZIS modified Pt and CoO_x photocatalyst by utilizing the impregnation-heat treatment method**. **a** TEM image of D-O-ZIS/Pt/CoO_x at low magnification; **b** HRTEM image of D-O-ZIS/Pt/CoO_x; **c** Scanning TEM image (Embedded in Supplementary Fig. 10a) and the corresponding EDX elemental mapping images of the distribution of Zn, In, S, O, Co, and Pt species on D-O-ZIS/Pt/CoO_x. The scale (20 nm) applies to the images in Supplementary Fig. 10c; **d** Time-dependent photocatalytic overall water splitting over D-O-ZIS/Pt/CoO_x in pure water under standard AM 1.5 illumination (100 mW cm^{-2}). 333 μL, 1.5 mg mL^{-1} H_2PtCl_6, 2.1 mg $Co(NO_3)_2$ $6H_2O$, the photocatalyst mass was 35 mg and the photocatalytic activity was evaluated via the total hydrogen and oxygen yield of a cycle, the time of each cycle is 12 h. Error bars represent the standard deviations from the statistic results of three sets of experiments.

We also used the impregnation-heat treatment method to load Pt and CoO_x nanoparticles onto D-O-ZIS [27, 28]. In detail, $Co(NO_3)_2·6H_2O$ (2.1 mg) and D-O-ZIS (35 mg) were dispersed in 50 mL of deionized water under continuous stirring. The solution was heated on a heating plate until complete

water evaporation, and the resulting precipitate was then subjected to thermal decomposition in a muffle furnace at 400 °C for 1 hour. Following the thermal treatment, D-O-ZIS/CoO_x was obtained. Afterward, the obtained D-O-ZIS/CoO_x photocatalyst and H_2PtCl_6 (333 μL, 1.5 mg mL^{-1}) were dispersed in an aqueous solution for Pt loading. Following light irradiation under standard AM1.5G illumination (100 mW cm^{-2}) for 1 hour, the photocatalyst was centrifuged, washed with deionized water, and then dried, resulting in the D-O-ZIS/Pt/CoO_x catalyst. The D-O-ZIS/Pt/CoO_x catalyst prepared using the impregnation-heat treatment method exhibited relatively large particles. Supplementary Fig. 10a, b displayed Pt particles around 6-8 nm and CoO_x nanoparticles about 7-12 nm in size with aggregation, attached to the D-O-ZIS surface. HRTEM revealed a lattice fringe distance of 0.22 nm corresponding to the (111) plane of Pt nanocrystals, and 0.25 nm for the (311) plane of CoO_x. The EDX elemental mapping results in Supplementary Fig. 10c confirm the presence of distributed CoO_x and Pt nanoparticles on the surface of D-O-ZIS.

Furthermore, the photocatalytic performance of the resultant D-O-ZIS/Pt/CoO_x photocatalyst, loaded with cocatalysts through the impregnation-heat treatment method, was also evaluated. As shown in Supplementary Fig. 10d, the D-O-ZIS/Pt/CoO_x exhibits photocatalytic H_2 and O_2 evolution rate of 23.2 and 11.4 μmol h^{-1}, respectively. The photocatalytic performance of D-O-ZIS/Pt/CoO_x, loaded with cocatalysts via the impregnation-heat treatment method, demonstrated a decreased photocatalytic activity compared with that prepared by impregnation-photo-deposition method (photocatalytic H_2 and O_2 evolution rate of 31.1 and 14.8 μmol h^{-1}).

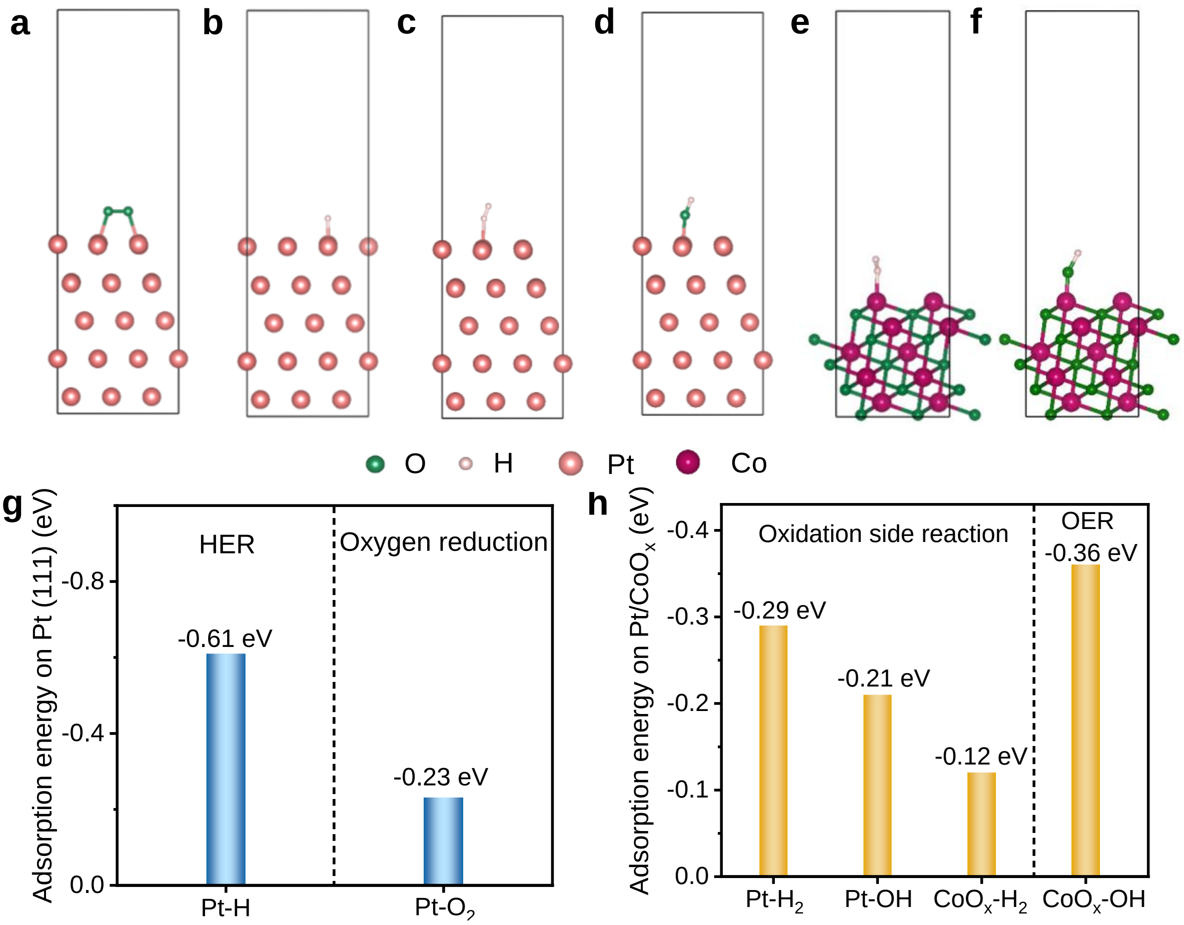

Supplementary Fig. 11. **DFT calculations of hydrogen-oxygen recombination reactions on D-O-ZIS/Pt/CoO_x photocatalyst. a, b** Configuration of O_2 and H adsorption model on Pt site; **c, d** Configuration of H_2 and OH adsorption model on Pt site; **e, f** Configuration of H_2 and OH adsorption model on CoO_x site; **g** Adsorption energy of O_2 and H adsorption model on Pt site; **h** Adsorption energy of H_2 and OH adsorption model on Pt and CoO_x sites.

We calculated the adsorption energies of O_2 and H on Pt, as well as the adsorption energies of H and OH on Pt and CoO_x. The adsorption models are shown in Supplementary Fig. 11a-f. Adsorption energy calculations indicate that, apart from H and OH adsorption, both Pt and CoO_x sites are capable of adsorbing H_2 and O_2, leading to the occurrence of hydrogen-oxygen evolution reactions, as well as hydrogen-oxygen recombination reactions (Supplementary Fig. 11g, h). The lower adsorption energies of H_2 and O_2 suggest that these species are less likely to adsorb on the surface and undergo subsequent reverse reactions. Pt-H exhibits a more negative adsorption energy of -0.61 eV, which is lower than the adsorption energies of Pt-H_2 (-0.29 eV) and Pt-O_2 (-0.23 eV). This indicates that the D-O-ZIS/Pt/CoO_x exhibits stronger adsorption ability for active hydrogen. Furthermore, CoO_x exhibits a more negative adsorption energy of -0.36 eV for OH, indicating that the D-O-ZIS/Pt/CoO_x surface is more favourable for the generation of O_2.

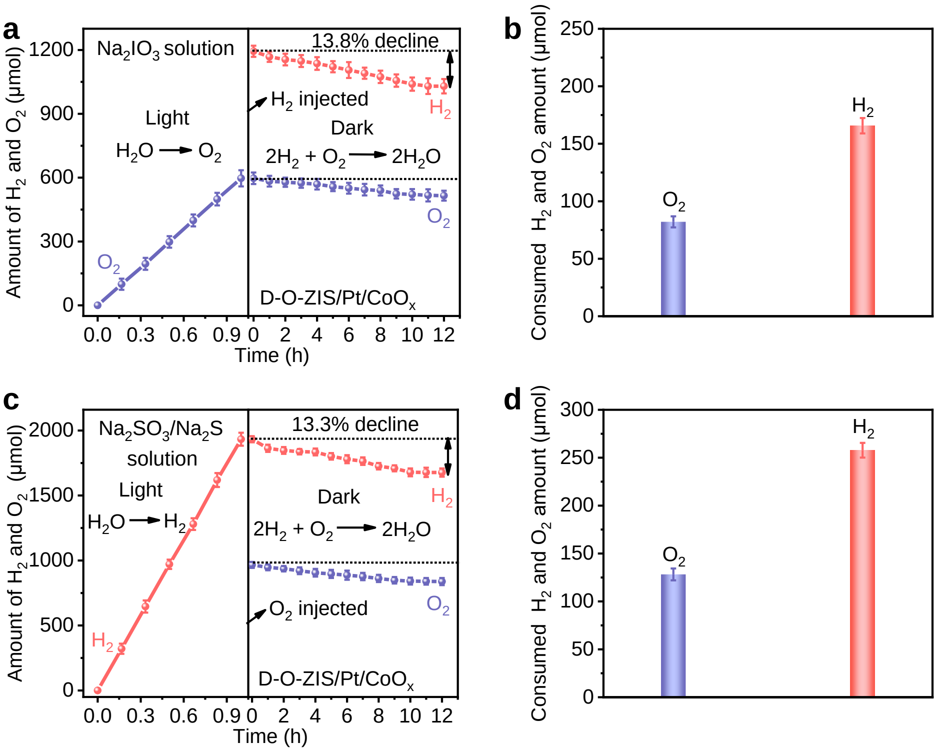

Supplementary Fig. 12. **Hydrogen-oxygen recombination reactions on D-O-ZIS/Pt/CoO_x photocatalyst. a** Hydrogen–oxygen recombination reaction after 1h photocatalytic oxygen evolution half-reaction, the injected H_2 amount is 1194 umol; **b** The corresponding amounts of H_2 and O_2 consumed in the hydrogen–oxygen recombination reaction; **c** Hydrogen–oxygen recombination reaction after 1h hydrogen evolution half-reaction, the injected O_2 amount is 966 umol; **d** The corresponding amounts of H_2 and O_2 consumed in the hydrogen–oxygen recombination reaction. Error bars in Supplementary Fig. 12 represent the standard deviations from the statistic results of three sets of experiments.

Supplementary Fig. 12a illustrates that D-O-ZIS/Pt/CoO_x generated O_2 gas of 597 umol after a 1-hour photocatalytic oxygen evolution half-reaction under standard AM1.5G illumination (100 mW cm^{-2}). Following this, illumination was removed, and about 1194 umol of H_2 was introduced in an approximate 2:1 ratio to the generated O_2 gas, initiating a subsequent dark reaction. Under dark conditions, the amounts of H_2 and O_2 gradually decreased with time at an approximate stoichiometric ratio of 2:1, confirming hydrogen-oxygen recombination. After 12 h dark reaction, about 165.7 umol of H_2 and 82.1 umol of O_2 gases were consumed, indicating a 13.8% decline of H_2 and O_2 gas (Supplementary Fig. 12a, b). Similarly, after hydrogen evolution half-reaction on D-O-ZIS/Pt/CoO_x, 966 umol O_2 was introduced in an approximate 1:2 ratio to the generated H_2 gas (1933 umol) under dark reaction and the H_2 and O_2 gas exhibits a 13.3% decline (Supplementary Fig. 12c, d). Thus, reverse hydrogen-oxygen recombination reaction occurs at D-O-ZIS/Pt/CoO_x surface, resulting in an approximate 13-14% decline after 12 h dark reaction [27].

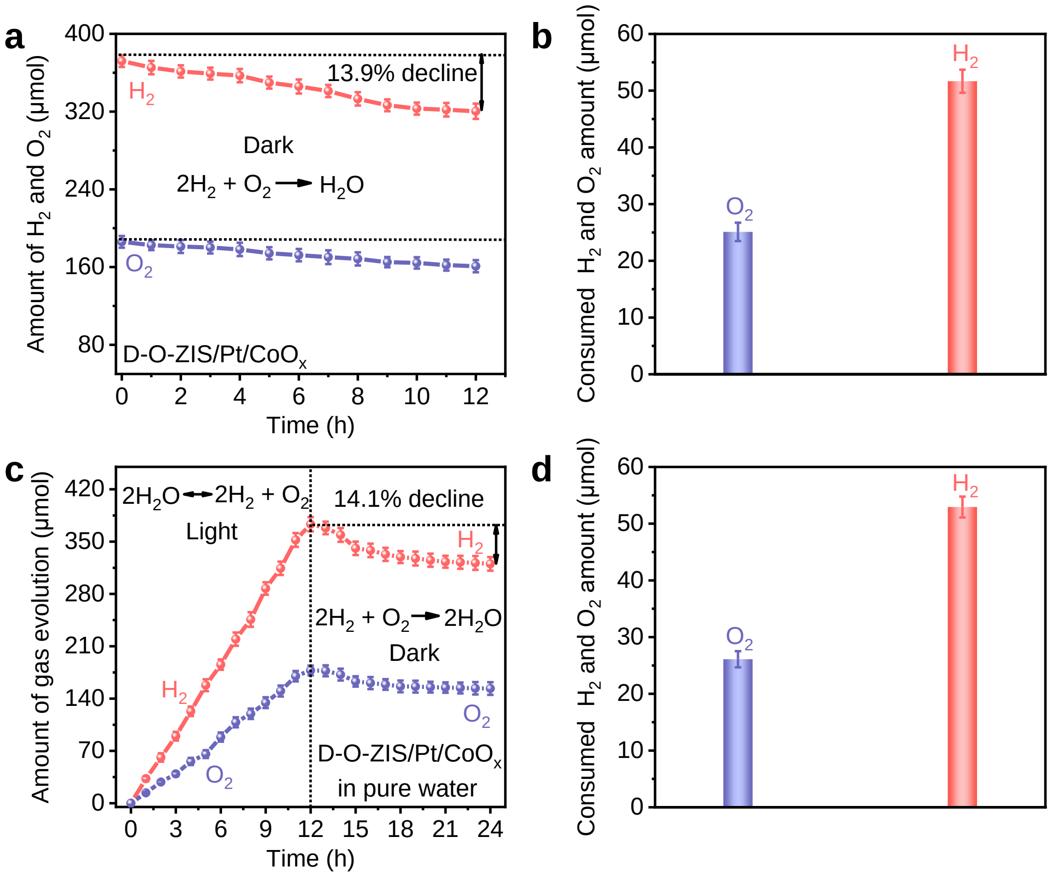

Supplementary Fig. 13. **Hydrogen-oxygen recombination reactions on D-O-ZIS/Pt/CoO_x photocatalyst. a** Hydrogen–oxygen recombination reaction by injecting stoichiometric amounts of H_2 and O_2 into the system, the injected H_2 amount is 372 umol and the injected O_2 amount is 186 umol; **b** The corresponding amounts of H_2 and O_2 consumed in the hydrogen–oxygen recombination reaction; **c** Hydrogen–oxygen recombination reaction after photocatalytic overall water splitting; **d** The corresponding amounts of H_2 and O_2 consumed in the hydrogen–oxygen recombination reaction. Error bars in Supplementary Fig. 13 represent the standard deviations from the statistic results of three sets of experiments.

As depicted in Supplementary Fig. 13a, b, we introduced a specific amount of H_2 (372 umol) and O_2 (186 umol) into the system at an approximate 2:1 ratio for a subsequent 12-hour dark reaction. The remaining H_2 and O_2 gases underwent chromatographic analysis. During the dark conditions, the amounts of H_2 and O_2 gradually decreased, indicating the occurrence of the reverse recombination reaction between hydrogen and oxygen. After 12 h of reaction, approximately 51.7 umol of H_2 and 25.3 umol of O_2 gases were consumed, reflecting a decrease of 13.9%.

We conducted photocatalytic overall water splitting followed by dark reactions on the D-O-ZIS/Pt/CoO_x catalyst to further study hydrogen-oxygen recombination. As shown in Supplementary Fig. 13c, d, initially, nearly stoichiometric amounts of H_2 (373 umol) and O_2 (178 umol) in a 2:1 ratio were generated under light. Upon removing light, H_2 and O_2 quantities gradually decreased while

maintaining an approximate 2:1 ratio. This gradual decline further illustrates the occurrence of hydrogen-oxygen recombination. Ultimately, around 52.9 umol of H_2 and 25.8 umol of O_2 gas were consumed, indicating a decrease of 14.1% after 12 h dark reaction, consistent with findings from injecting fixed H_2 and O_2 amounts for recombination. Hence, combined with the O_2 and H_2 adsorption energy results (Supplementary Fig. 11), we can conclude that the generated H_2 and O_2 will recombine at Pt site of D-O-ZIS/Pt/CoO_x during photocatalytic process with the proportion of hydrogen-oxygen recombination occurring at approximately 14% after 12 h dark reaction [27, 28].

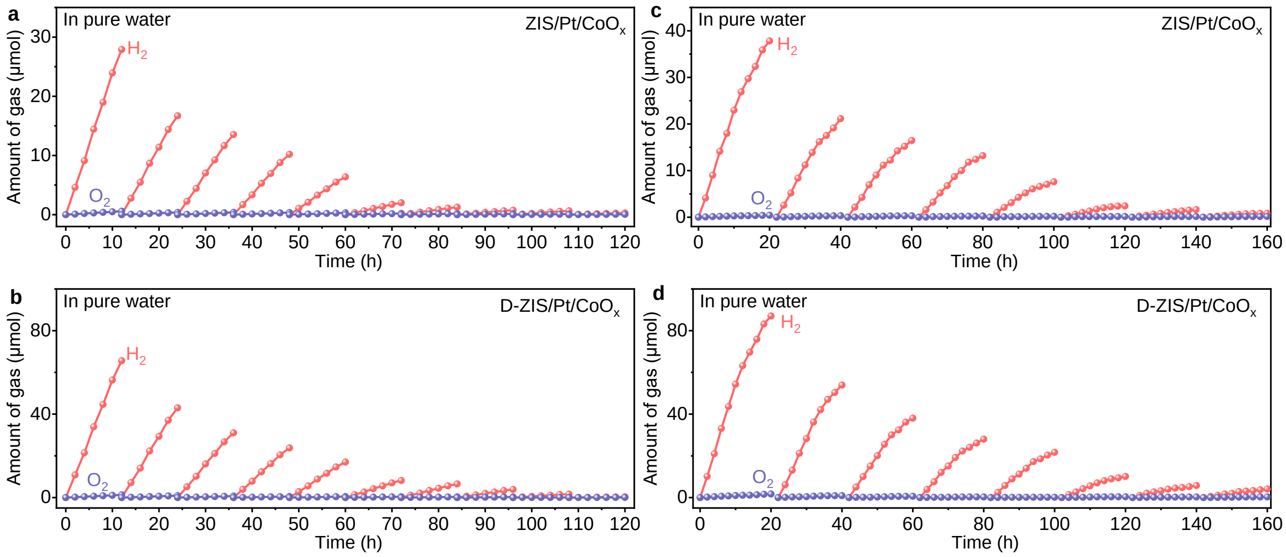

Supplementary Fig. 14. **Photocatalytic overall water splitting performance.** Time-dependent photocatalytic overall water splitting over **a** ZIS and **b** D-ZIS, in pure water under standard AM 1.5 illumination (100 mW cm^{-2}), Pt and CoO_x used as cocatalysts, Pt to CoO_x wt% ratio of 1:4, the photocatalyst mass was 35 mg and the photocatalytic activity was evaluated via the total hydrogen and oxygen yield of a cycle, the time of each cycle is 12 h. **c, d** Time-dependent photocatalytic overall water splitting over ZIS and D-ZIS with the time of each cycle of 20 h.

We extend the duration of each cycle from 10 to 20 hours to observe degrees of attenuation at each cycle. As shown in Supplementary Fig. 14c, d, ZIS and D-ZIS with cocatalysts loading exhibit attenuation of photocatalytic activity when extending the cycle time to 20 h. Thus, after a prolonged reaction time, the catalyst structure of ZIS and D-ZIS becomes deactivated and unstable, resulting in decreased and poor photocatalytic performance.

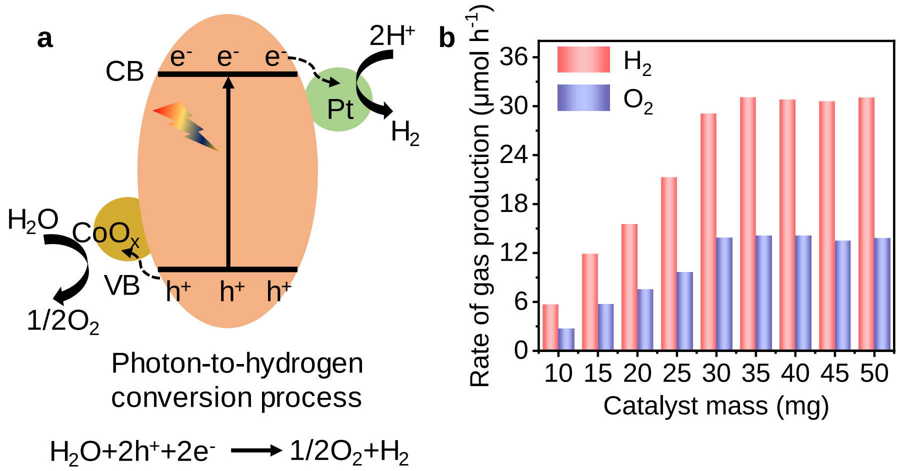

Supplementary Fig. 15. **Photocatalyst for the AQY in overall water splitting performance. a** Schematic illustration of AQY calculated following one-step excitation process [17]; **b** The overall water-splitting performance of D-O-ZIS/Pt/CoO_x with different catalyst masses (detail seen in Supplementary Table 4), Pt and CoO_x as cocatalysts with the wt% ratio of Pt:CoO_x of 1:4.

Supplementary Table 4. The overall water splitting performance of D-O-ZIS with different masses. Pt and CoO_x as cocatalyst and wt% ratio of 1:4.

Catalyst mass (mg)	H_2 evolution rate (μmol h^{-1})	O_2 evolution rate (μmol h^{-1})
10	5.7	2.5
15	11.9	5.6
20	15.5	7.4
25	21.3	9.5
30	29.1	13.5
35	31.1	14.6
40	29.3	14.1
45	28.9	13.2
50	28.3	13.6

Supplementary Table 5. Calculated AQY values of D-O-ZIS/Pt/CoO_x.

Wavelengths (λ, nm)	H_2 evolved (μmol h^{-1})	Catalyst mass (mg)	Light power (mW)	AQY (%)
400	26.3	35	29.3	14.90
420	21.2	35	27.3	12.31
425	18.9	35	26.5	11.20
450	14.2	35	26.2	8.04
500	12.8	35	30.0	5.68
550	8.1	35	29.8	3.33

The AQY could be calculated according to the equations (2) and (3). The AQY@400 nm of D-O-ZIS/Pt/CoO_x was calculated [1, 4]:

$$N = \frac{S \times P \times \lambda \times t}{h \times c} = \frac{29.3 \times 10^{-3} \times 400 \times 10^{-9} \times 3600}{6.626 \times 10^{-34} \times 3 \times 10^{8}} = 2.122 \times 10^{20}$$

$$AQY(\%) = \frac{\text{number of reacted electrons}}{\text{number of incident photons}} \times 100\%$$

$$= \frac{\text{number of evolved hydrogen molecules} \times 2}{N} \times 100\%$$

$$= \frac{6.02 \times 10^{23} \times 26.3 \times 10^{-6} \times 2}{2.122 \times 10^{20}} \times 100\%$$

$$= 14.90\%$$

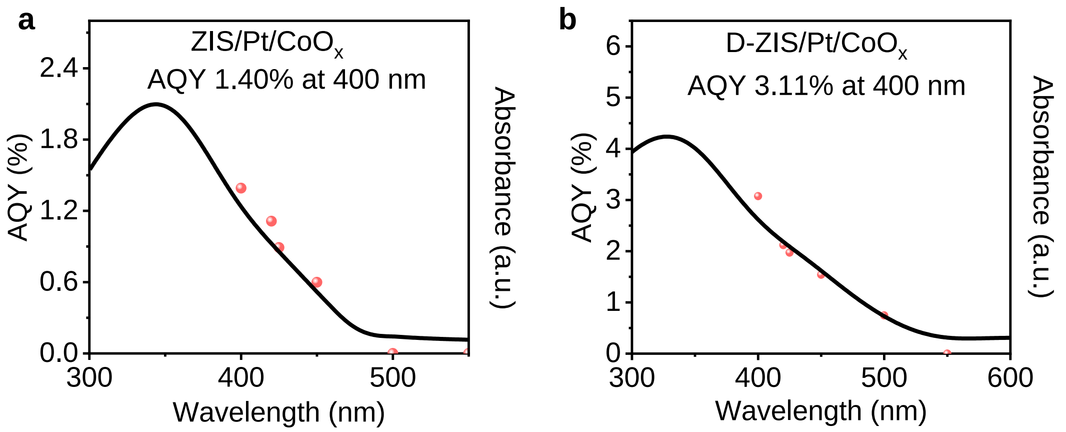

Supplementary Fig. 16. **Wavelength-dependent of AQY during photocatalytic overall water-splitting. a** ZIS, **b** D-ZIS. AQY denotes the apparent quantum yield that was calculated using equations (2) and (3) in Supporting Information following the one-step excitation process, Pt and CoO_x used as cocatalysts, Pt to CoO_x wt% ratio of 1:4.

Supplementary Table 6. Calculated AQY values of ZIS/Pt/CoO_x.

Wavelengths (λ, nm)	H_2 evolved (μmol h^{-1})	Catalyst mass (mg)	Light power (mW)	AQY (%)
400	2.4	35	29.3	1.40
420	1.9	35	27.3	1.11
425	1.5	35	26.5	0.89
450	1.0	35	26.2	0.59
500	0	35	30.0	0
550	0	35	29.8	0

The AQY@400 nm of ZIS/Pt/CoO_x was calculated:

$$N = \frac{S \times P \times \lambda \times t}{h \times c} = \frac{29.3 \times 10^{-3} \times 400 \times 10^{-9} \times 3600}{6.626 \times 10^{-34} \times 3 \times 10^{8}} = 2.122 \times 10^{20}$$

$$AQY(\%) = \frac{\text{number of reacted electrons}}{\text{number of incident photons}} \times 100\%$$

$$= \frac{\text{number of evolved hydrogen molecules} \times 2}{N} \times 100\%$$

$$= \frac{6.02 \times 10^{23} \times 2.4 \times 10^{-6} \times 2}{2.122 \times 10^{20}} \times 100\%$$

$$= 1.4\%$$

Supplementary Table 7. Calculated AQY values of D-ZIS/Pt/CoO_x.

Wavelengths (λ, nm)	H_2 evolved (μmol h^{-1})	Catalyst mass (mg)	Light power (mW)	AQY (%)
400	5.4	35	29.3	3.11
420	3.6	35	27.3	2.12
425	3.3	35	26.5	1.97
450	2.7	35	26.2	1.54
500	1.6	35	30.0	0.73
550	0	35	29.8	0

The AQY@400 nm of D-ZIS/Pt/CoO_x was calculated:

$$N = \frac{S \times P \times \lambda \times t}{h \times c} = \frac{29.3 \times 10^{-3} \times 400 \times 10^{-9} \times 3600}{6.626 \times 10^{-34} \times 3 \times 10^{8}} = 2.122 \times 10^{20}$$

$$AQY(\%) = \frac{\text{number of reacted electrons}}{\text{number of incident photons}} \times 100\%$$

$$= \frac{\text{number of evolved hydrogen molecules} \times 2}{N} \times 100\%$$

$$= \frac{6.02 \times 10^{23} \times 5.4 \times 10^{-6} \times 2}{2.122 \times 10^{20}} \times 100\%$$

$$= 3.11\%$$

Supplementary Table 8. Calculated STH values of D-O-ZIS/Pt/CoO_x for photocatalytic overall water splitting.

NO.	H_2 evolution rate (μmol h^{-1})	O_2 evolution rate (μmol h^{-1})	STH (%)
1	31.1	14.8	0.57
2	31.0	13.7	0.57
3	31.1	13.8	0.57
4	31.5	14.4	0.58
5	31.6	14.5	0.58
6	30.7	13.7	0.56
7	32.2	14.5	0.59
8	30.8	13.7	0.56
9	31.5	14.3	0.58
10	30.8	13.6	0.56
11	30.2	13.5	0.55
12	32.7	14.7	0.60

STH was calculated with cocatalysts of Pt and CoO_x, Pt to CoO_x wt% ratio of 1:4 [3, 4]:

$$\text{STH (\%)} = \frac{\text{energy of generated H}_2}{\text{Solar energy irradiating the reactor}} \times 100\%$$

$$= \frac{n \times \Delta G}{P \times S} \times 100\%$$

$$= \frac{31.1 \times 10^{-6} \times 237130}{100 \times 10^{-3} \times 3.6 \times 3600} \times 100\% = 0.57\%$$

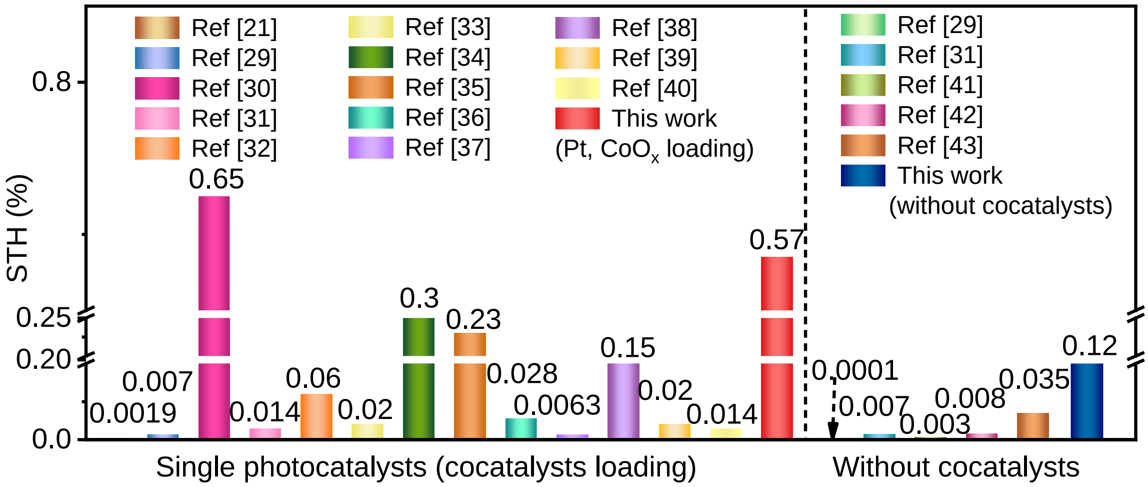

Supplementary Fig. 17. Comparison of photocatalytic overall water splitting for the single photocatalysts. (see Supplementary Table 9 for details). We calculated STH values using the provided equation for references that contained relevant parameters.

Supplementary Table 9. Comparation of photocatalytic overall water splitting in reported single photocatalysts.

Catalysts	Reaction conditions	H_2 evolution rate (μmol h^{-1})	O_2 evolution rate (μmol h^{-1})	AQY (%)	STH (%)	STH (without co-catalyst)	Ref.
D-O-ZIS	300 W Xe lamp, 0.035 g catalyst, 2wt% Pt and 8wt% CoO_x as cocatalysts	31.1 (λ>300 nm)	14.8 (λ>300 nm)	14.90@400 nm	0.57	0.12	This work
F-CN	300 W Xe lamp, 0.030 g catalyst, 3wt% Pt as co-catalyst.	5.33 (λ>300 nm)	1.39 (λ>300 nm)	0.57@400 nm	0.0019	/	[21]
$Y_2Ti_2O_5S_2$	300 W Xe lamp, 0.04 g catalyst, 1 wt% IrO_2 and 1.5 wt% Rh/Cr_2O_3 as cocatalyst.	31 (λ>420 nm)	15 (λ>420 nm)	0.36@420 nm<br>0.23@500 nm<br>0.05@600 nm	0.007	0.0001	[29]
$SrTiO_3$:Al	300 W Xe lamp, 0.1 g catalyst, 0.05 wt% Cr_2O_3, and 0.1 wt% Rh as cocatalyst.	2800 (λ>420 nm)	1400 (λ>420 nm)	95.7@350 nm<br>95.9@360 nm<br>91.6@365 nm	0.65	/	[30]
$[Pb_8I_8(H_2O)_3]^{8-}$ $O_2C(CH_2)_4CO_4$	300 W Xe lamp, 0.1 g catalyst, Rh as cocatalyst.	9.0 (λ>420 nm)	4.7 (λ>420 nm)	0.13@320 nm	0.014	0.007	[31]
3D g-C_3N_4 NS	300 W Xe lamp, 0.05 g catalyst, Pt and IrO_2 as cocatalyst.	5.07 (λ>420 nm)	2.46 (λ>420 nm)	1.40@420 nm	0.06	/	[32]
$ZnTiO_{3-x}N_y$	300 W Xe lamp, 0.05 g catalyst, Pt and RhO_x as cocatalyst.	8.5 (λ>420 nm)	3.7 (λ>420 nm)	0.22@420 nm	0.02	/	[33]
$SrTiO_3$	300 W Xe lamp, 0.05 g catalyst, $RhCrO_x$ as cocatalyst.	33 (λ>420 nm)	17 (λ>420 nm)	0.5@420 nm	0.3	/	[34]
Pt@TpBpy-NS	300 W Xe lamp, 0.08 g catalyst, Pt as cocatalyst.	1.98 (λ>420 nm)	0.9 (λ>420 nm)	2.8@450 nm	0.23	/	[35]
CTF-0	300 W Xe lamp, 0.01 g catalyst, 3 wt% Pt, 6 wt% Co_3O_4 as cocatalysts	0.82 (λ>420 nm)	0.4 (λ>420 nm)	/	0.028	/	[36]
$SrTaO_2N$	300 W Xe lamp, 0.15 g catalyst, Ir/IrO_x, Ru/RuO_2, CrO_xas cocatalysts	7.86 (λ>420 nm)	3.06 (λ>420 nm)	0.34@450 nm	0.0063	/	[37]
CdS	300 W Xe lamp, 0.1 g catalyst,	17 (λ>420 nm)	8.3 (λ>420 nm)	0.27@380 nm	0.15	/	[38]

	Ru(tpy)(bpy)Cl_2, Pt nanoparticles as cocatalysts.						
g-C_3N_4	300 W Xe lamp, 0.2 g catalyst, Pt and CoO_x as cocatalyst.	1.20 (λ>420 nm)	0.60 (λ>420 nm)	0.3@405 nm	0.02	/	[39]
Ta_3N_5	300 W Xe lamp, 0.2 g catalyst, Rh and Cr_2O_3 as cocatalyst.	33 (λ>420 nm)	12 (λ>420 nm)	2.2@320 nm	0.014	/	[40]
Ag/$ZnIn_2S_4$	300 W Xe lamp, 0.005 g catalyst.	0.283 (λ>420 nm)	0.145 (λ>420 nm)	0.70@400 nm 0.57@420 nm	/	0.003	[41]
Ni-Co LDH	300 W Xe lamp, 0.1 g catalyst.	3.63 (λ>420 nm)	1.80 (λ>420 nm)	1.38@380 nm	/	0.008	[42]
Oligo (phenylene butadiynylene) (OPB)	300 W Xe lamp, 0.05 g catalyst	1.88 (λ>420 nm)	0.95 (λ>420 nm)	/	/	0.035	[43]

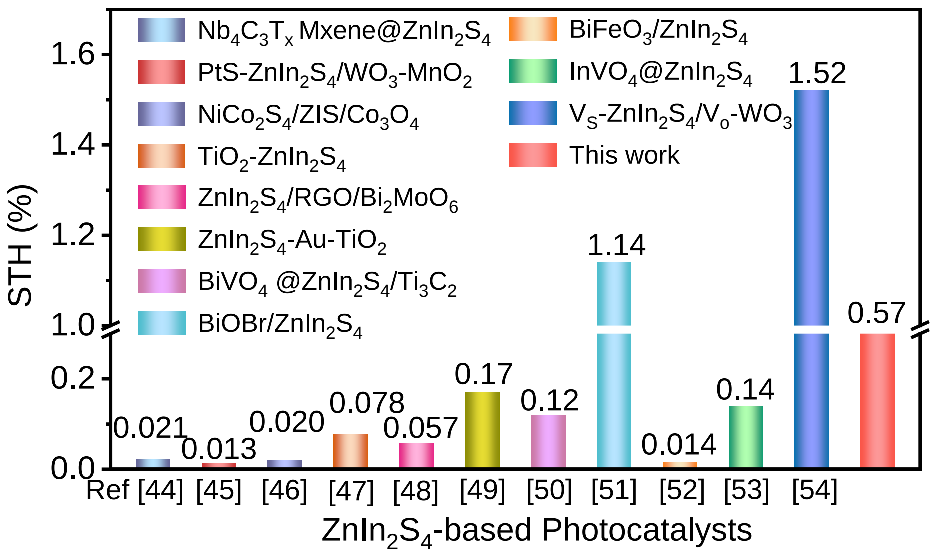

Supplementary Fig. 18. Comparison of photocatalytic overall water splitting for the $ZnIn_2S_4$-based nanocomposites photocatalysts. (see Supplementary Table 10 for details). We calculated STH values using the provided equation for references that contained relevant parameters.

Supplementary Table 10. Comparation of photocatalytic overall water splitting in reported $ZnIn_2S_4$-based nanocomposites photocatalysts.

Catalysts	Reaction conditions	H_2 evolution rate (μmol h^{-1})	O_2 evolution rate (μmol h^{-1})	AQY (%)	STH (%)	Ref.
D-O-ZIS	300 W Xe lamp, 0.035 g catalyst, 2wt% Pt and 8wt% CoO_x as cocatalysts	31.1 (λ>300 nm)	14.8 (λ>300 nm)	14.90@400 nm	0.57	This work
$Nb_4C_3T_x$ Mxene@$ZnIn_2S_4$	300 W Xe lamp, 0.02 g catalyst,	1.07 (λ>420 nm)	0.53 (λ>420 nm)	1.2@380 nm	0.021	[44]
PtS-$ZnIn_2S_4$/WO_3-MnO_2	300 W Xe lamp, 0.05 g catalyst, 0.5% PtS and 3.0% MnO_2 as cocatalyst.	0.74 (λ>420 nm)	0.28 (λ>420 nm)	0.50@420 nm	0.013	[45]
$NiCo_2S_4$/ZIS/Co_3O_4	300 W Xe lamp, 0.1 g catalyst, Pt, and Co_3O_4 as cocatalyst.	1.06 (λ>400 nm)	0.28 (λ>400 nm)	13.5@400 nm	0.020	[46]
TiO_2-$ZnIn_2S_4$	300 W Xe lamp, 0.02 g catalyst	4.29 (λ>420 nm)	1.63 (λ>420 nm)	36.17@365 nm	0.078	[47]
$ZnIn_2S_4$/RGO/Bi_2MoO_6	300 W Xe lamp, 0.1 g catalyst, Pt and CoO_x as cocatalyst.	3.08 (λ>420 nm)	1.56 (λ>420 nm)	/	0.057	[48]
$ZnIn_2S_4$-Au-TiO_2	300 W Xe lamp, 0.05 g catalyst	9.32 (λ>420 nm)	3.31 (λ>420 nm)	/	0.17	[49]
$BiVO_4$ @$ZnIn_2S_4$/Ti_3C_2	300 W Xe lamp, 0.06 g catalyst	6.16 (λ>400 nm)	3.05 (λ>400 nm)	2.4@410 nm	0.12	[50]
BiOBr/$ZnIn_2S_4$	300 W Xe lamp, 0.1 g catalyst, Pt as cocatalyst.	62.8 (λ>420 nm)	30.4 (λ>420 nm)	8.57@420 nm	1.14	[51]
$BiFeO_3$/$ZnIn_2S_4$	300 W Xe lamp, 0.012 g catalyst.	0.77 (λ>420 nm)	0.38 (λ>420 nm)	24.28@365 nm	0.014	[52]
$InVO_4$@$ZnIn_2S_4$	300 W Xe lamp, 0.005 g catalyst.	7.86 (λ>420 nm)	3.06 (λ>420 nm)	0.34@450 nm	0.140	[53]
Sulfur-Deffcient $ZnIn_2S_4$/Oxygen-Deffcient WO_3	300 W Xe lamp, 0.035 g catalyst. Pt and CoO_x as cocatalyst.	169.2 (λ>420 nm)	86.5 (λ>420 nm)	3.4@380 nm	1.52	[54]

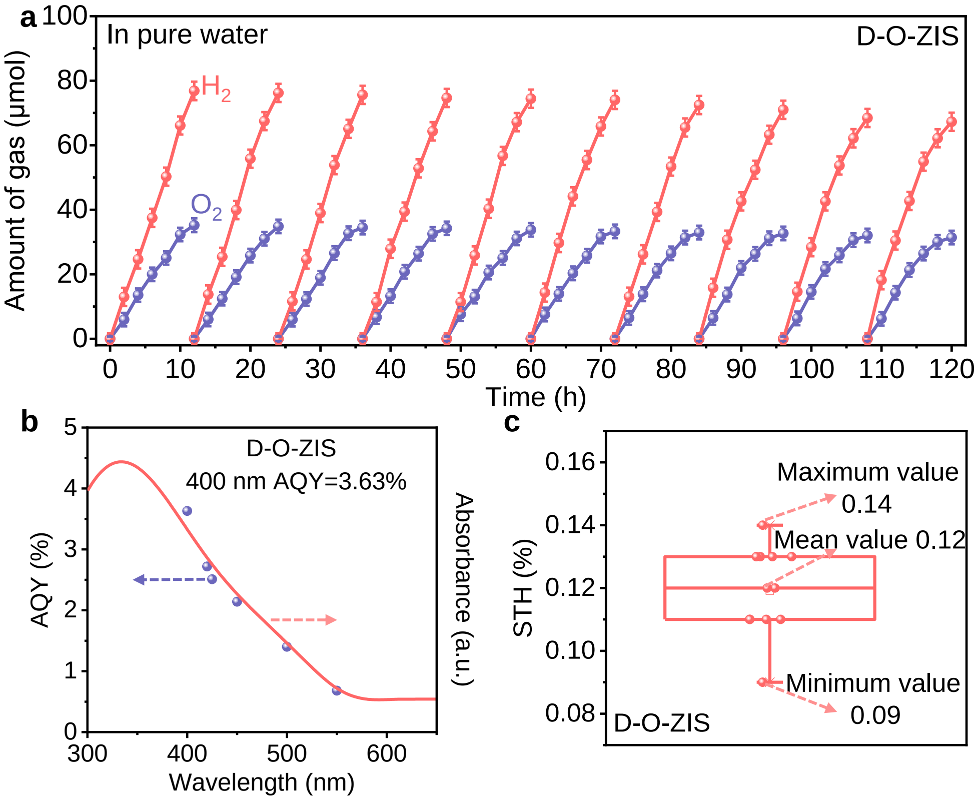

Supplementary Fig. 19. **Photocatalytic overall water splitting performance without cocatalysts loading. a** Time-dependent photocatalytic overall water splitting over D-O-ZIS in pure water under standard AM 1.5 illumination (100 mW cm^{-2}), the photocatalyst mass was 35 mg and the photocatalytic activity was evaluated via the total hydrogen and oxygen yield of a cycle, the time of each cycle is 12 h; Error bars represent the standard deviations from the statistic results of three sets of experiments. **b** Wavelength-dependent of AQY during photocatalytic overall water splitting based on D-O-ZIS. AQY denotes the apparent quantum yield that was calculated using equations (2) and (3) in Supporting Information following the one-step excitation process and shown in Supplementary Table 11; **c** The STH efficiency of D-O-ZIS for photocatalytic overall water splitting. The STH value was evaluated 12 times with separate samples as shown in Supplementary Table 12 and calculated using Equation (1) in main text. The centre line represents the median, the top and bottom box limits represent the upper and lower quartile, respectively, the small rectangle represents the mean value and the maximum/minimum values are indicated by the top/bottom bars.

Supplementary Table 11. Calculated AQY values of single D-O-ZIS without cocatalysts loading.

Wavelengths (λ, nm)	H_2 evolved ($\mu mol\ h^{-1}$)	Catalyst mass (mg)	Light power (mW)	AQY (%)
400	6.4	35	29.3	3.63
420	4.6	35	27.3	2.72
425	4.2	35	26.5	2.51
450	3.7	35	26.2	2.14
500	3.1	35	30.0	1.40
550	1.6	35	29.8	0.68

The AQY@400 nm of D-O-ZIS without cocatalysts loading was calculated:

$$N = \frac{S \times P \times \lambda \times t}{h \times c} = \frac{29.3 \times 10^{-3} \times 400 \times 10^{-9} \times 3600}{6.626 \times 10^{-34} \times 3 \times 10^{8}} = 2.122 \times 10^{20}$$

$$AQY(\%) = \frac{\text{number of reacted electrons}}{\text{number of incident photons}} \times 100\%$$

$$= \frac{\text{number of evolved hydrogen molecules} \times 2}{N} \times 100\%$$

$$= \frac{6.02 \times 10^{23} \times 6.4 \times 10^{-6} \times 2}{2.122 \times 10^{20}} \times 100\%$$

$$= 3.63\%$$

Supplementary Table 12. Calculated STH values of single D-O-ZIS without cocatalysts loading.

NO.	H_2 evolution rate (μmol h^{-1})	O_2 evolution rate (μmol h^{-1})	STH (%)
1	6.4	3.0	0.12
2	6.3	2.9	0.12
3	7.0	3.2	0.13
4	7.5	3.3	0.14
5	6.9	3.2	0.13
6	5.8	2.5	0.11
7	5.9	2.7	0.11
8	4.8	2.1	0.09
9	5.8	2.4	0.11
10	7.0	3.2	0.13
11	6.9	3.3	0.13
12	5.8	2.6	0.11

The STH of D-O-ZIS without cocatalysts loading was calculated was calculated:

$$\text{STH (\%)} = \frac{\text{energy of generated } H_2}{\text{Solar energy irradiating the reactor}} \times 100\%$$

$$= \frac{n \times \Delta G}{P \times S} \times 100\%$$

$$= \frac{6.4 \times 10^{-6} \times 237130}{100 \times 10^{-3} \times 3.6 \times 3600} \times 100\% = 0.12\%$$

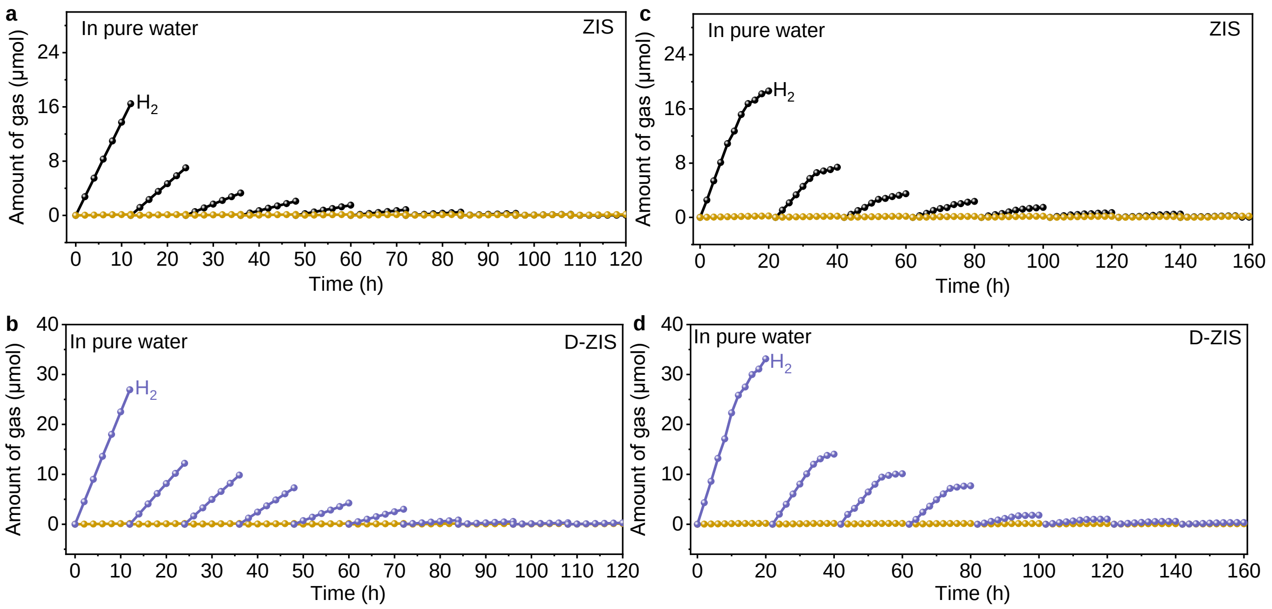

Supplementary Fig. 20. **The overall water splitting performance of ZIS and D-ZIS without cocatalysts loading.** Time-dependent photocatalytic overall water splitting over **a** ZIS and **b** D-ZIS in pure water. Reaction conditions: under standard AM1.5G illumination (100 mW cm^{-2}), the photocatalyst mass is 35 mg and the photocatalytic activity is evaluated via the total hydrogen and oxygen yield of a cycle, the time of each cycle is 12 h. Time-dependent photocatalytic overall water splitting over **c** ZIS and **d** D-ZIS with the time of each cycle of 20 h.

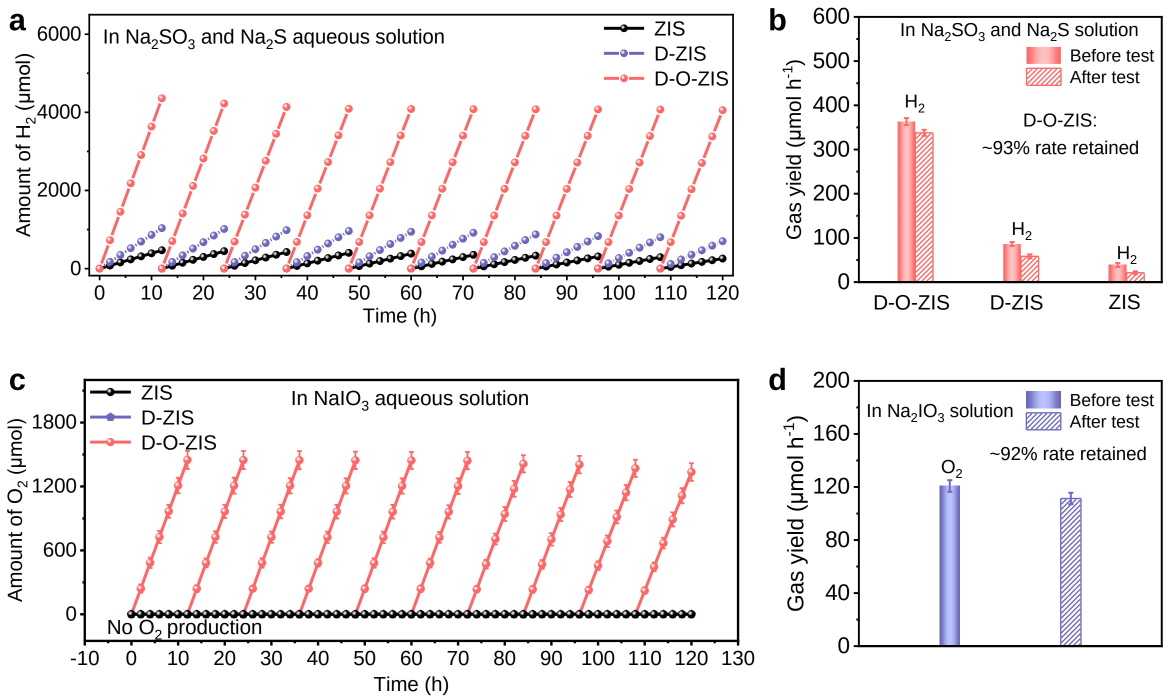

Supplementary Fig. 21. **The H_2 or O_2 evolution half reactions of photocatalysts. a** Time-dependent photocatalytic hydrogen gas evolution half-reaction for ZIS, D-ZIS and D-O-ZIS in the presence of sacrificial reagents (Na_2SO_3 and Na_2S solution); **b** Photocatalytic hydrogen gas evolution rate of ZIS, D-ZIS and D-O-ZIS before and after 120 h photocatalytic tests. Error bars represent the standard deviations from the statistic results of three sets of experiments; **c** Time-dependent photocatalytic O_2 evolution half-reaction for ZIS, D-ZIS and D-O-ZIS in the presence of sacrificial reagents (20 mM $NaIO_3$ aqueous solution). Error bars represent the standard deviations from the statistic results of three sets of experiments; **d** Photocatalytic O_2 gas evolution rate of D-O-ZIS before and after photocatalytic tests. Under standard AM 1.5 illumination (100 mW cm^{-2}), the photocatalyst mass is 35 mg and the photocatalytic activity is evaluated via the total oxygen yield of a cycle, the time of each cycle is 12 h. Error bars represent the standard deviations from the statistic results of three sets of experiments.

/

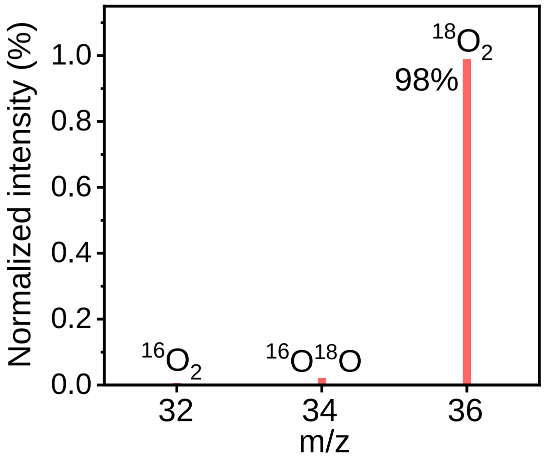

Supplementary Fig. 22. The mass spectrum of oxygen gas evolved for photocatalytic $H_2{}^{18}O$ splitting.

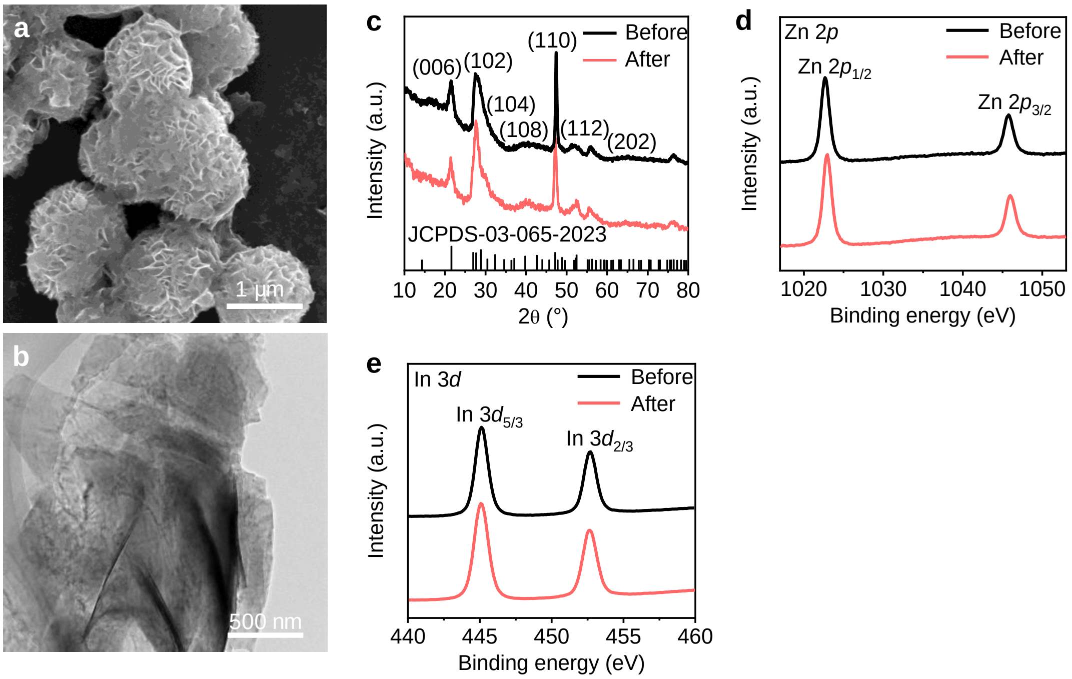

Supplementary Fig. 23. **Structural stability of the D-O-ZIS after 120 h photocatalytic test**. **a** SEM image of D-O-ZIS after photocatalytic testing; **b** TEM image of D-O-ZIS after photocatalytic testing; **c** XRD patterns of D-O-ZIS before and after photocatalytic testing; **d** XPS spectra of Zn 2*p* in D-O-ZIS before and after photocatalytic testing; **e** XPS spectra of In 3*d* in D-O-ZIS before and after photocatalytic testing.

As shown in Supplementary Fig. 23, after the photocatalytic reaction of D-O-ZIS, the catalyst maintains its flower-like layered structure (Supplementary Fig. 23a, b). The intensity of XRD peaks for D-O-ZIS remains almost unchanged before and after the reaction, with negligible shifts, indicating the preservation of the crystal structure (Supplementary Fig. 23c). Furthermore, the XPS spectra of Zn 2*p* and In 3*d* demonstrate minimal changes before and after the photocatalytic reaction (Supplementary Fig. 23d, e).

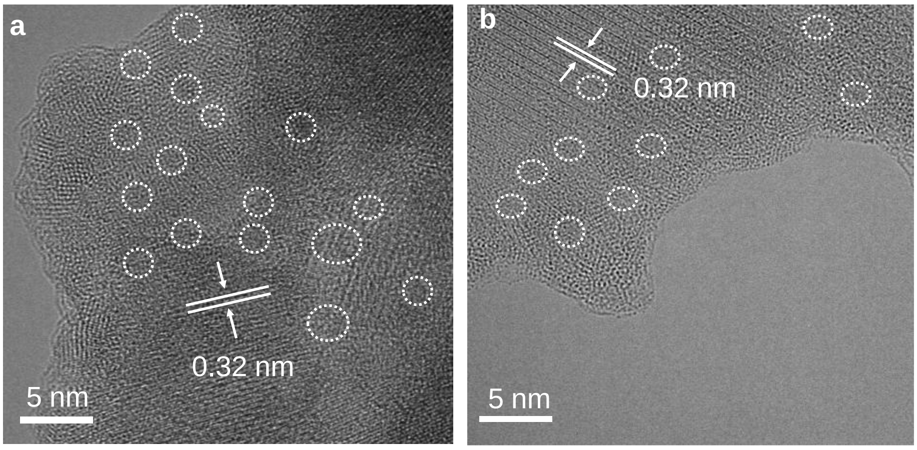

Supplementary Fig. 24. **HRTEM images of after testing. a** ZIS; **b** D-ZIS.

ZIS and D-ZIS suffer from corrosion under light irradiation, during which lattice S^{2-} ions are more easily escaped and oxidized by photoexcited holes into SO_4^{2-}, thus leading to deactivation of photocatalysts. The HRTEM image of ZIS and D-ZIS after testing revealed S leaching features, forming S atom loss structures and the shell thickness for D-ZIS is destroyed.

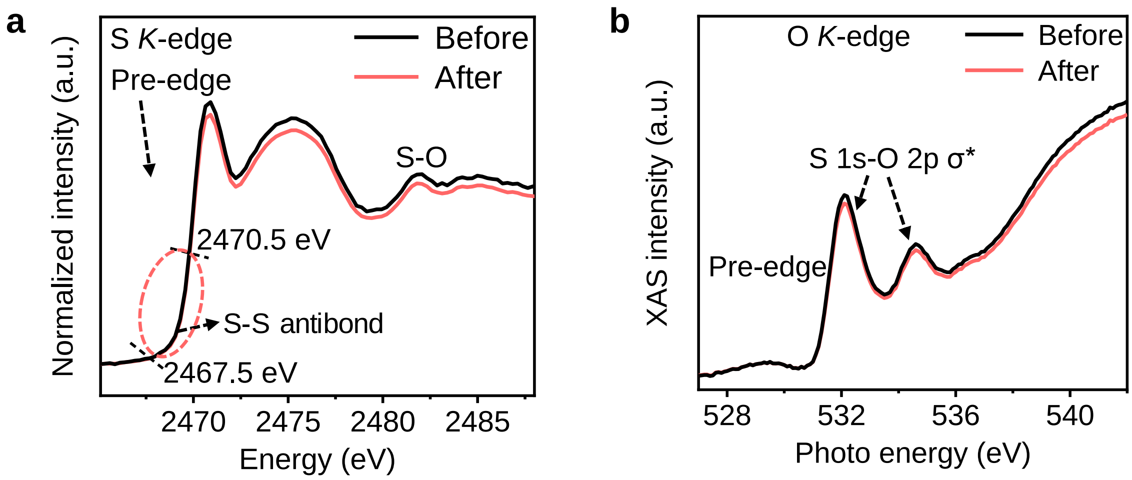

Supplementary Fig. 25. **Structural stability of the D-O-ZIS after 120 h photocatalytic test**. **a** The XANES spectra of S *K*-edge of D-O-ZIS before and after 120 h photocatalytic test; **b** Normalized O *K*-edge XANES spectra of D-O-ZIS before and after 120 h photocatalytic test.

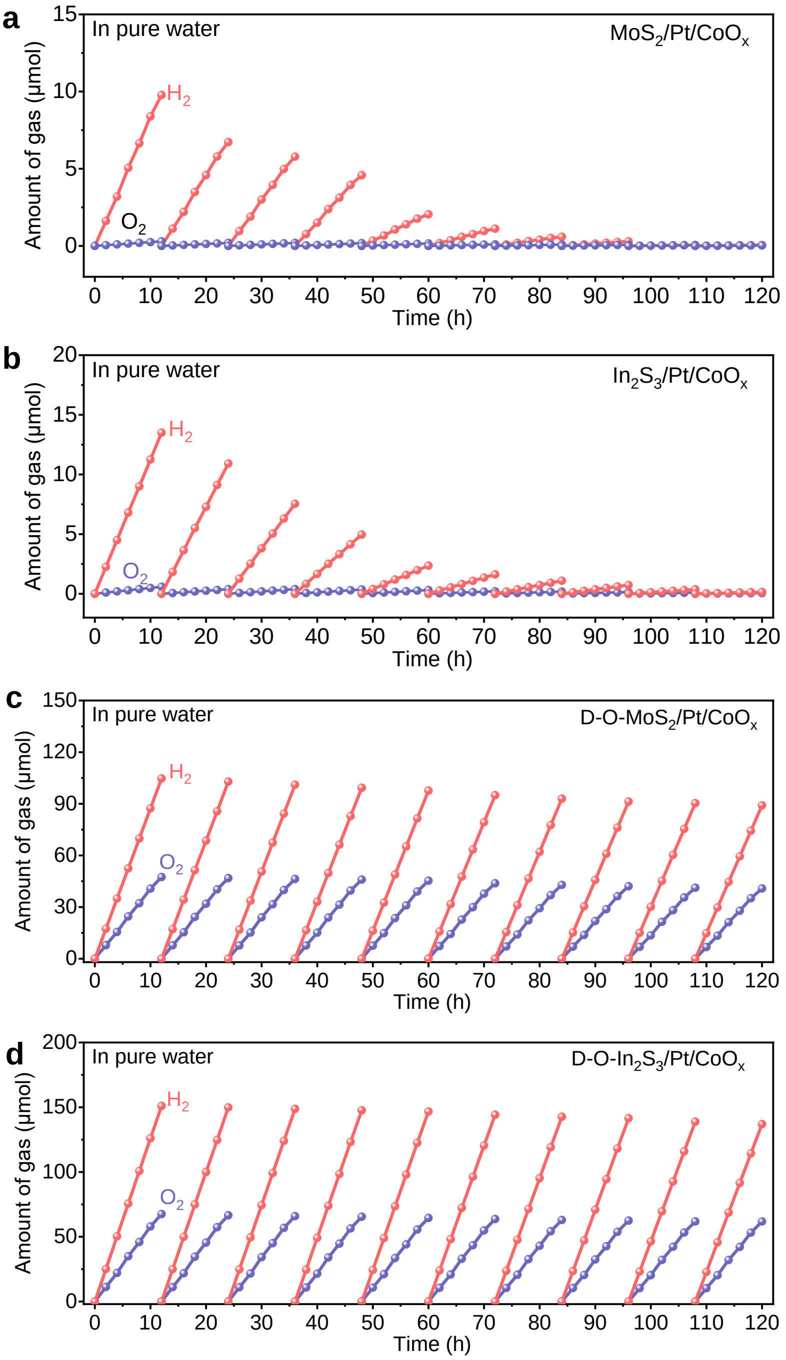

Supplementary Fig. 26. **The universality of overall water-splitting performance for metal sulfides photocatalysts.** **a** Time-dependent photocatalytic overall water splitting over $MoS_2/Pt/CoO_x$ in pure water under; **b** Time-dependent photocatalytic overall water splitting over $In_2S_3/Pt/CoO_x$ in pure water. **c** Time-dependent photocatalytic overall water splitting over D-O-$MoS_2/Pt/CoO_x$ in pure water; **d** Time-dependent photocatalytic overall water splitting over D-O-$In_2S_3/Pt/CoO_x$ in pure water. (Light intensity: AM 1.5 illumination (100 mW cm^{-2}), Pt to CoO_x wt% ratio of 1:4, the photocatalyst mass was 35 mg and the photocatalytic activity was evaluated via the total hydrogen and oxygen yield of a cycle, the time of each cycle is 12 h).

MoS_2 and In_2S_3 are typical photocatalysts among metal sulfides for hydrogen production. We obtained D-MoS_2, which exhibited substantial structural distortion through cathodic electrolysis [55]. We then treated the D-MoS_2 with oxygen plasma to introduce oxygen doping, resulting in the creation

of a photocatalyst with a distorted structure and O doping, called D-O-MoS_2. Additionally, we prepared metal sulfide In_2S_3 [56] and created a distortion state and oxygen doping on In_2S_3 through thermally induced atomic migration to obtain D-O-In_2S_3 photocatalyst.

We then investigated the photocatalytic performance of MoS_2/Pt/CoO_x and In_2S_3/Pt/CoO_x were under light irradiation at AM 1.5G (100 mW cm^{-2}) with Pt and CoO_x used as cocatalysts. Their performance decreased greatly and decayed to zero after 120 h. Almost no oxygen was produced on these photocatalysts. However, when we introduced distortion states and cation-site O doping into MoS_2, H_2 and O_2 were steadily produced over D-O-MoS_2, with H_2 and O_2 evolution amounts up to 104.6 and 47.4 μmol, respectively, accompanying ~87% of its original photocatalytic gas evolution rate, demonstrating excellent stability in overall water splitting performance. The solar to hydrogen (STH) efficiency was measured at AM1.5G (100 mW cm^{-2}) simulated sunlight irradiation with a mean value of 0.16%. Similarly, D-O-In_2S_3 exhibited H_2 and O_2 evolution amounts up to 151.1 and 67.5 μmol, respectively, and showed ~89% of its original photocatalytic gas evolution rate with high stability. The STH efficiency yielded value of 0.24%. By applying distortion and oxygen doping strategies to MoS_2 and In_2S_3 photocatalysts, their oxygen-inert basal planes were activated, achieving efficient photocatalytic overall water splitting with high stability and demonstrating the universality of this strategy.

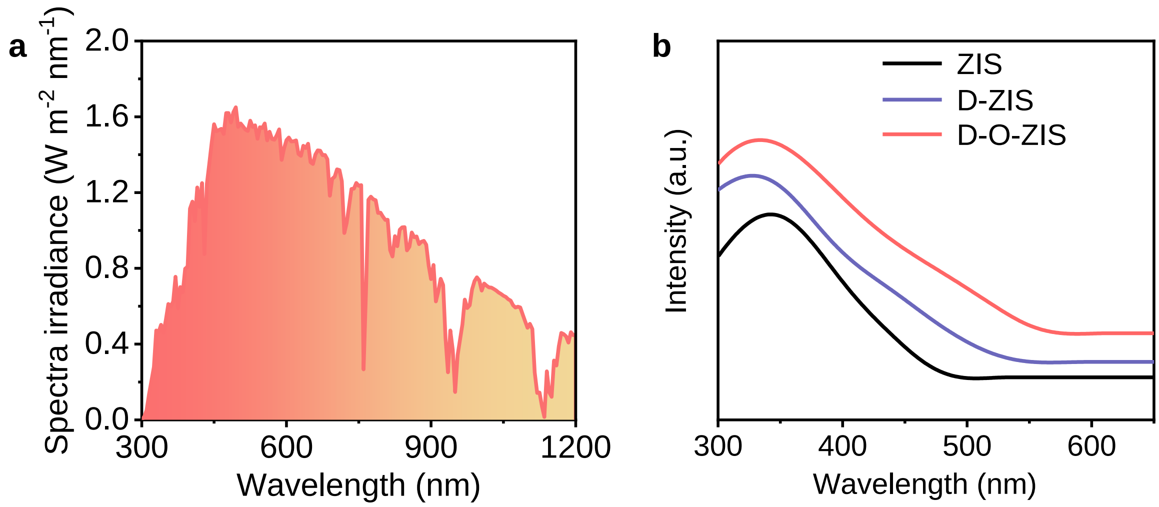

Supplementary Fig. 27. **The optical properties for photocatalysts. a** Spectrum irradiation intensity; **b** UV-vis absorption spectra of ZIS, D-ZIS, and D-O-ZIS.

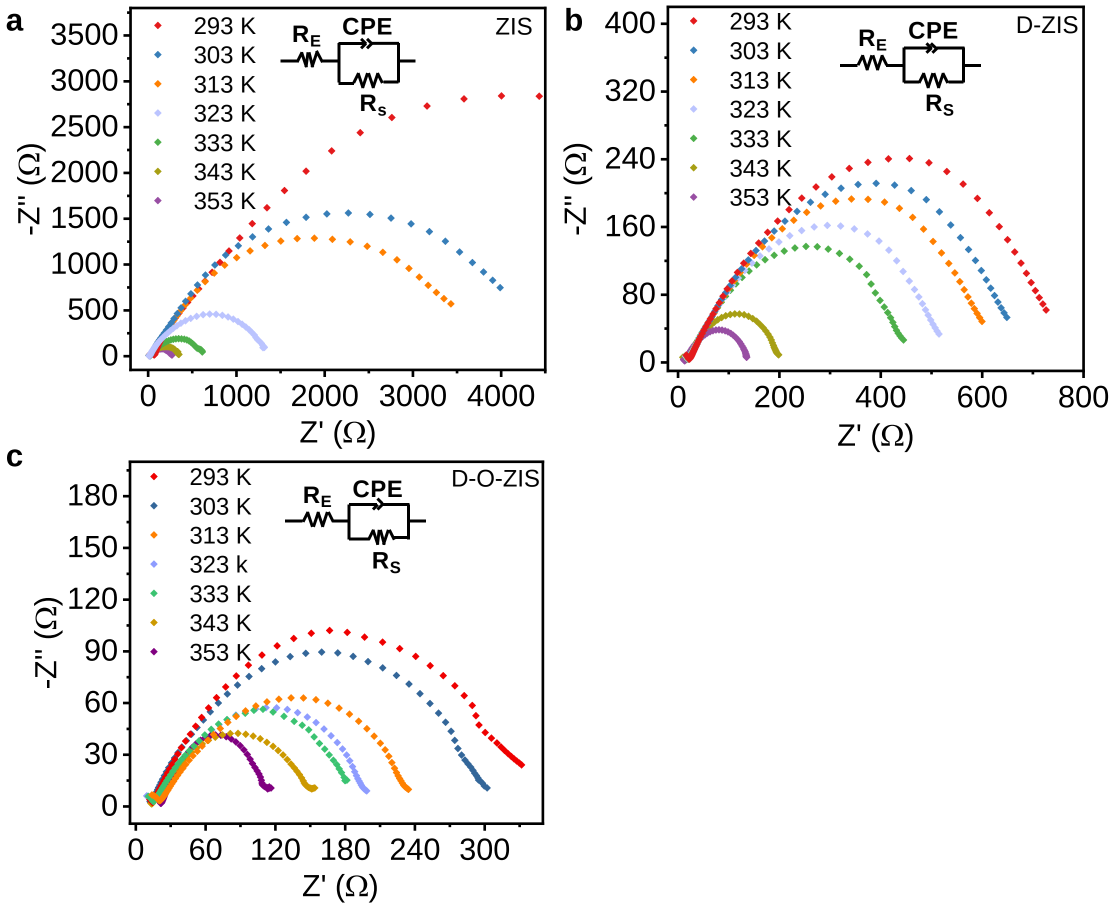

Supplementary Fig. 28. **The kinetics of charge transport for photocatalysts.** In-situ EIS plots under different temperatures (293 K, 303 K, 313 K, 323 K, 333 K, 343 K, and 353 K) for **a** ZIS; **b** D-ZIS; and **c** D-O-ZIS. The inset shows the equivalent circuit, where R_E, R_S, and CPE represent the electrolyte resistance, electrode resistance, and constant phase element, respectively.

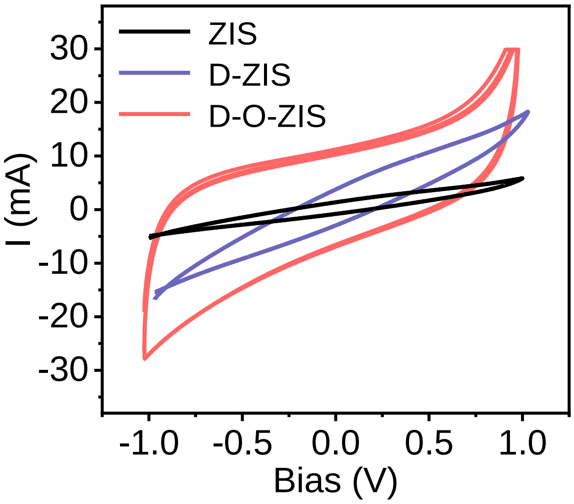

Supplementary Fig. 29. **The conductivity test.** Cyclic voltammetry at scanning rate of 50 mV s^{-1} for ZIS, D-ZIS, and D-O-ZIS.

We measured the capacitance-voltage (CV) using a device similar to a physical plane capacitor with a Ti/photocatalyst/In metal/dielectric/metal structure to elucidate the charge of internal electric field [7]. To improve the contact between the tip and the semiconductor film, we deposited an indium particle on the top of the photoelectrode. We found that pasting soft indium particles onto the electrode further enhanced the contact between the tip and the photocatalyst film. The use of solid-state indium prevented penetration through the film and short-circuiting of the bottom Ti foil. Our results showed that the internal electric field intensity was proportional to the potential shift from 0 V to the bias intersection voltage (V_i) in each V–log I curve, as seen in Fig. 4b from the CV measurement.

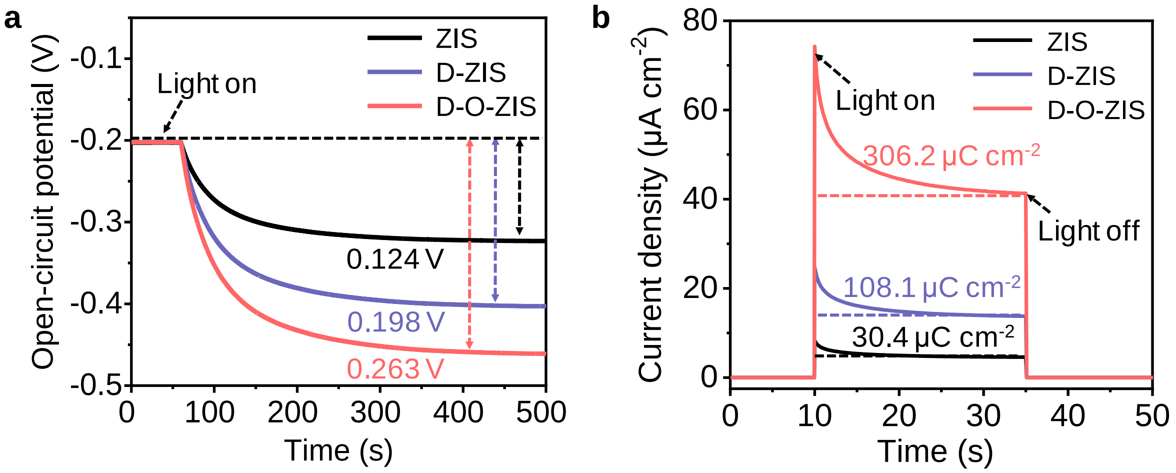

Supplementary Fig. 30. **The internal electric field intensity determination. a** The surface voltage of ZIS, D-ZIS, and D-O-ZIS; **b** The surface accumulated electrons density of ZIS, D-ZIS, and D-O-ZIS.

We calculated the internal electric field intensity of samples via transient photocurrent density and surface voltages according to kanetal model, which outlined in the Methods section. The surface voltages of ZIS, D-ZIS, and D-O-ZIS was 0.124, 0.198, and 0.263 V, respectively. The surface charge densities were then calculated by integrating the transient anodic photocurrent peaks. D-O-ZIS exhibited the highest integral value (306.2 $\mu C \cdot cm^{-2}$) among all specimens, which was 2.8 times that of D-ZIS (108.1 $\mu C \cdot cm^{-2}$), and 10 times that of ZIS (30.4 $\mu C \cdot cm^{-2}$). Thus, the D-O-ZIS showed the strongest internal electric field, which was 5.1 times stronger than that of ZIS and 2.0 times stronger than that of D-ZIS.

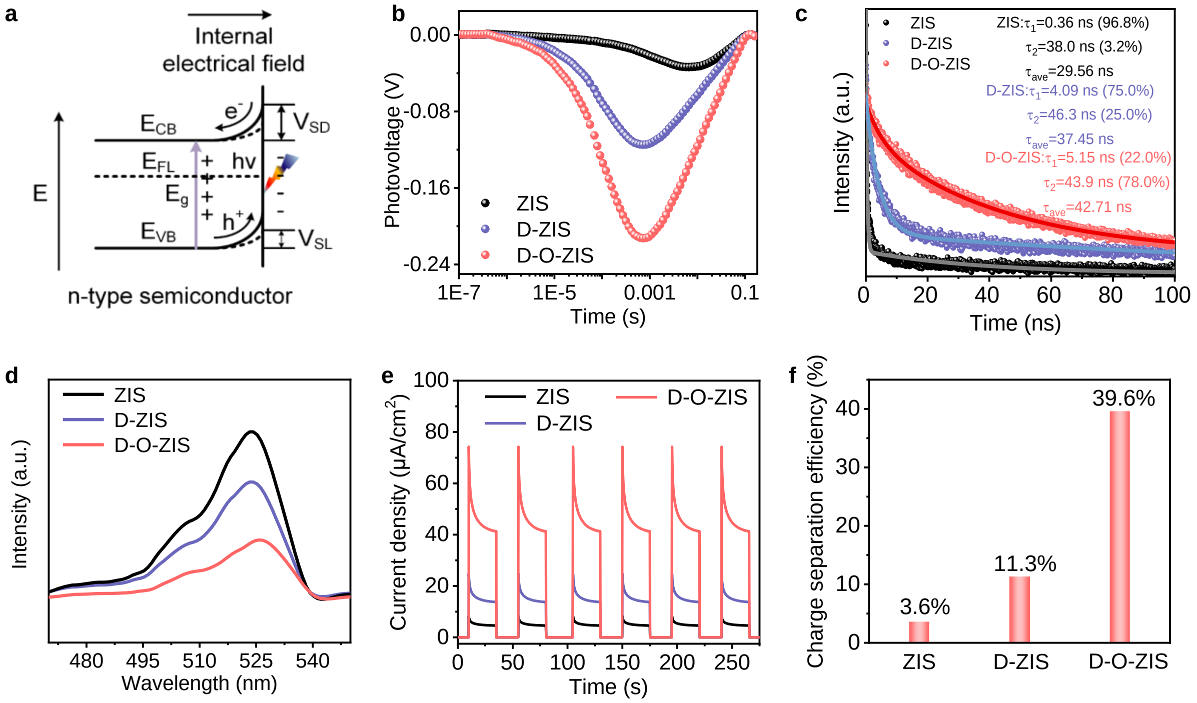

Supplementary Fig. 31. **The kinetics of charge separation for photocatalysts.** **a** Schematic illustration of the TPV generated in n-type semiconductor. Note that the more positive the TPV signal, the more efficient for the charge separation for an n-type semiconductor [8]; **b** TPV spectra of ZIS, D-ZIS and D-O-ZIS; **c** TRPL of ZIS, D-ZIS and D-O-ZIS; **d** PL spectra of ZIS, D-ZIS and D-O-ZIS (excited at 375 nm); **e** Photocurrent response of ZIS, D-ZIS and D-O-ZIS; **f** Charge separation efficiency of ZIS, D-ZIS and D-O-ZIS.

Supplementary Fig. 31a, b displays negative photovoltage resulting from photoelectron accumulation on the photocatalyst surface. The efficiency of charge separation was significantly improved by the robust internal electric field induced by the distortion states and cation-site O doping. To validate the photocarrier separation efficiency of the samples, TRPL and steady-state PL spectra were performed in Supplementary Fig. 31c [19-21]. The D-O-ZIS exhibited a prolonged lifetime with a value of 42.71 ns, which is longer than that of D-ZIS (37.45 ns) and ZIS (29.56 ns). Supplementary Fig. 31d shows the recorded PL spectra. The introduction of O-doping caused significant PL quenching in D-O-ZIS, resulting in the lowest PL intensity and a slight red shift. This indicates that there was an improvement in charge separation in D-O-ZIS. To determine the charge separation efficiency, an electrochemical method was used (details can be found in the Supporting Texts section). The results showed that D-O-ZIS had a maximum charge separation efficiency of 39.6%, which was 11.0 times higher than ZIS and 3.5 times higher than D-ZIS.

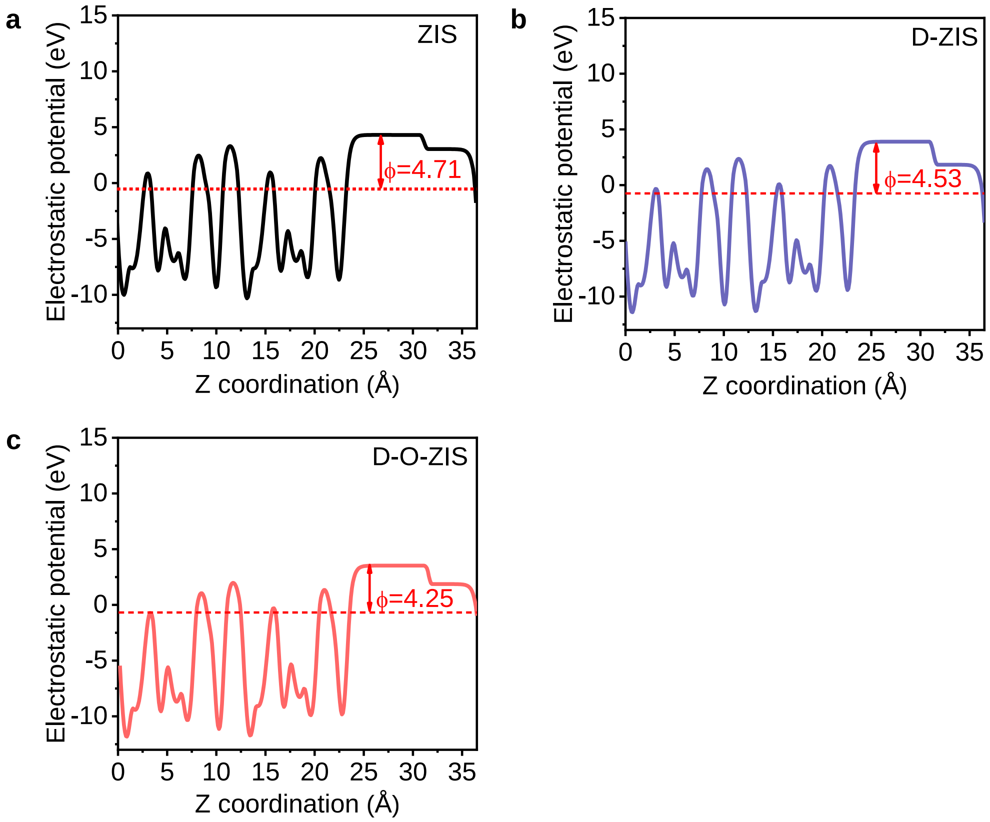

Supplementary Fig. 32. **The calculated average potential. a** ZIS; **b** D-ZIS; **c** D-O-ZIS. Calculated electrostatic potential profiles averaged on the plane perpendicular to the c-axis for the samples were obtained. The average potential reflects changes in the work function for the samples. The work functions for ZIS, D-ZIS, and D-O-ZIS were φ = 4.71, 4.53, and 4.25 eV, respectively. The obtained E_F was aligned at 0.21, 0.03, and -0.25 eV for ZIS, D-ZIS, and D-O-ZIS, respectively. This is consistent with the trends of the E_F obtained from experiment in Fig. 4d.

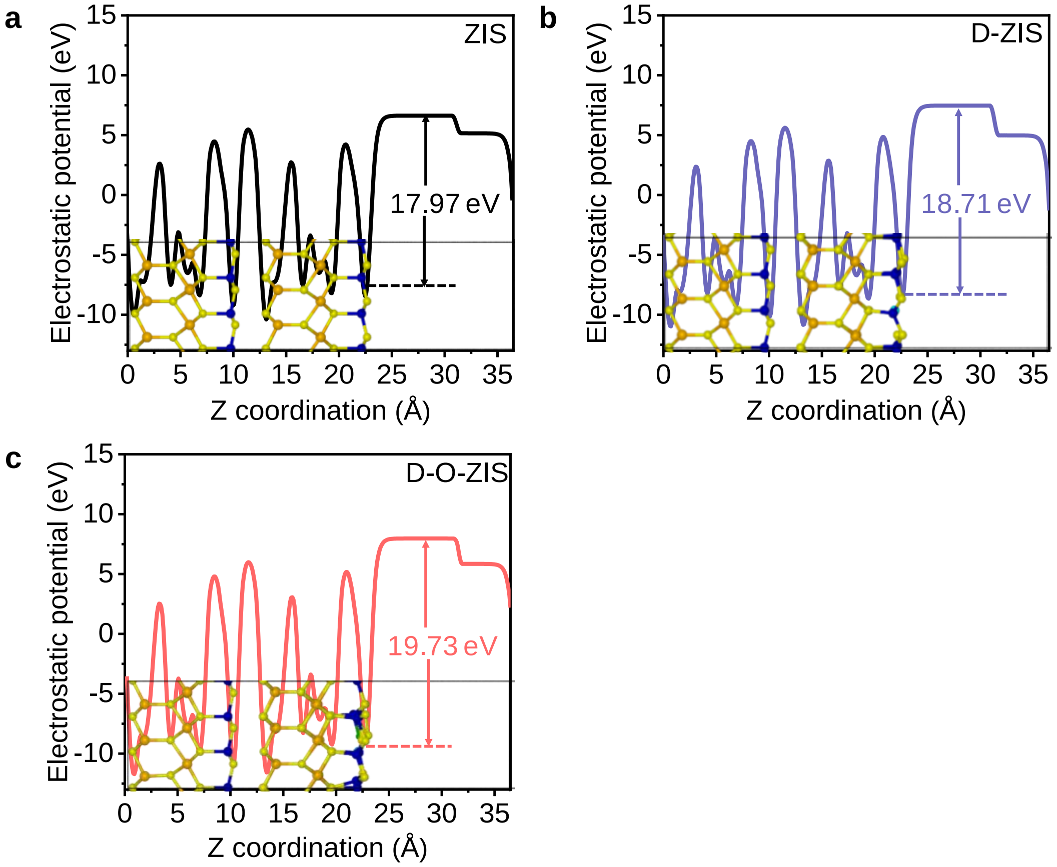

Supplementary Fig. 33. **Internal electric field determinated by DFT** [24]. **a** DFT calculation of the local internal electric field for ZIS; **b** DFT calculation of the local internal electric field for D-ZIS; **c** DFT calculation of the local internal electric field for D-O-ZIS.

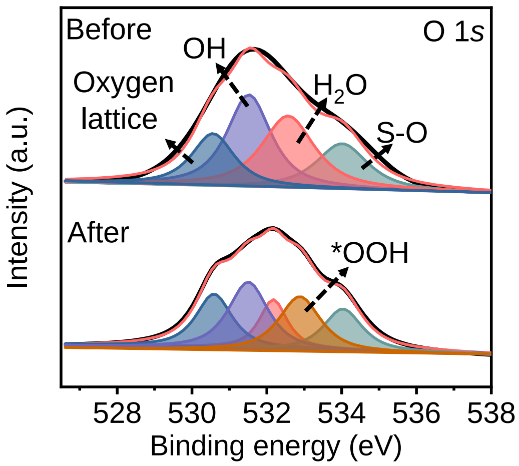

Supplementary Fig. 34. O 1*s* XPS spectra of D-O-ZIS before and after 120 h photocatalytic test.

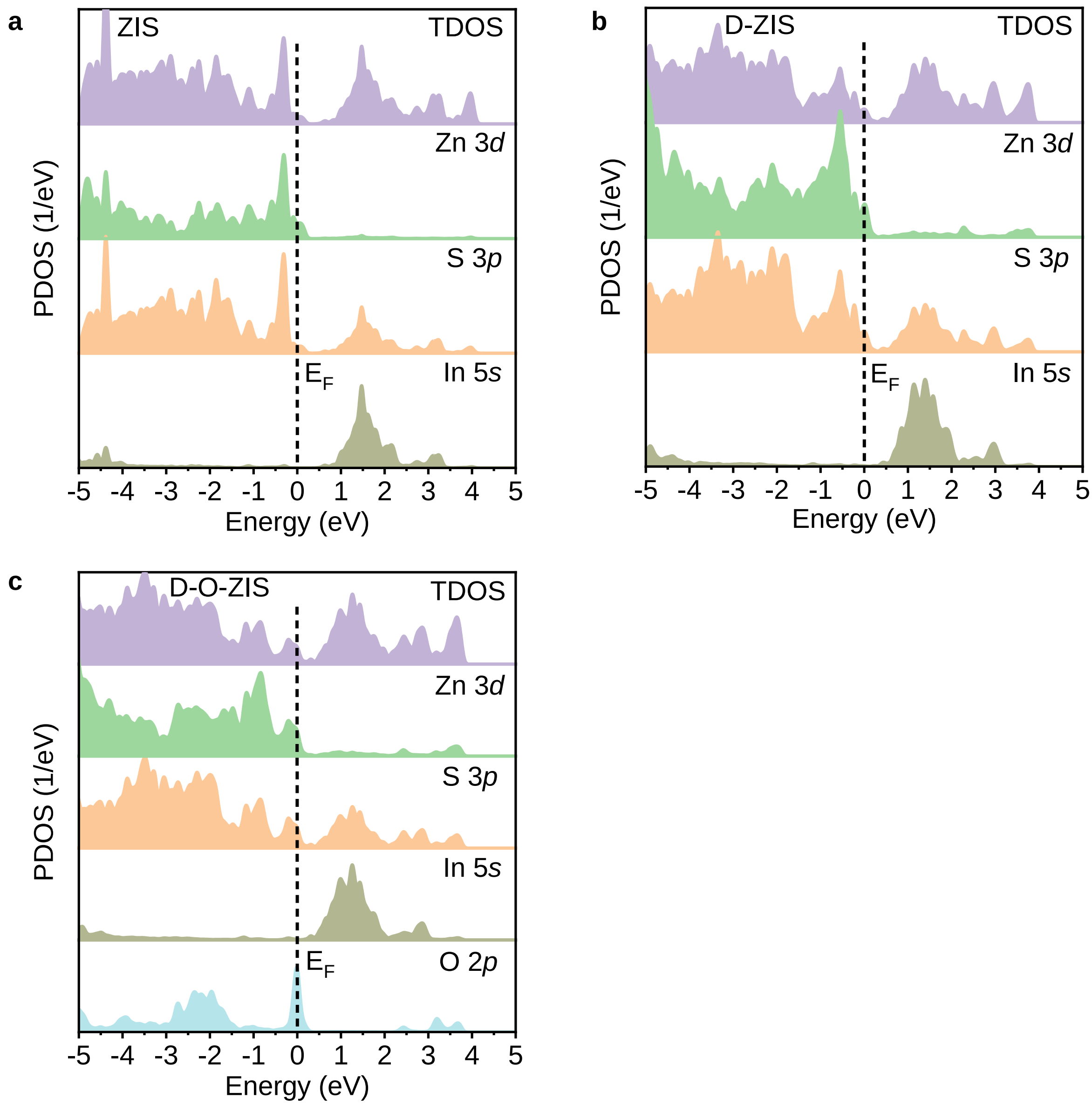

Supplementary Fig. 35. **Local structure activation determinated by DFT**. **a** The PDOS calculation of ZIS; **b** The PDOS calculation of D-ZIS; **c** The PDOS calculation of D-O-ZIS.

We investigate the effect of O doping on the electronic structure of D-O-ZIS. Our findings suggest that the coordinated O atoms hybridize with the *p* band of S atom, causing the adsorbate state to split into localized bonding and antibonding states. This results in an intense state at the Fermi level. We analyzed the total and partial density of states (TDOS and PDOS) profiles for D-O-ZIS and found that the dominant feature is S 3*p*-O 2*p* bonding resonances of O 2*p* near the Fermi level, which form hybridized electronic states. These states act as electron acceptors, enhancing the conductivity of D-O-ZIS and inhibiting the recombination of electron-hole pairs [23].

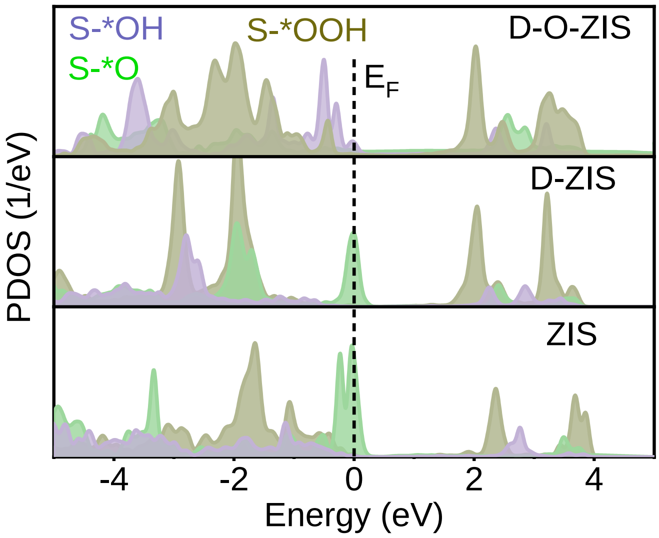

Supplementary Fig. 36. PDOS of S 3*p* bands in ZIS, D-ZIS, and D-O-ZIS for the intermediates of *OH, *O, and *OOH.

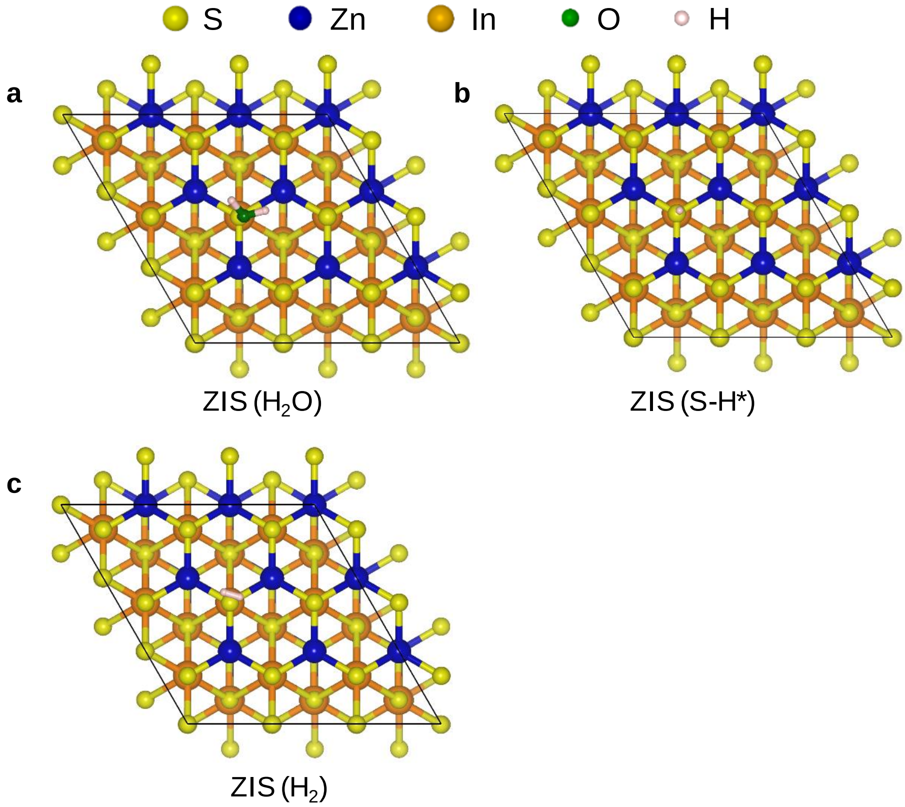

Supplementary Fig. 37. **DFT adsorption models of ZIS in the photocatalytic HER process**. **a** ZIS (H_2O); **b** ZIS (S-H*); **c** ZIS (H_2). The models are displayed in top view.

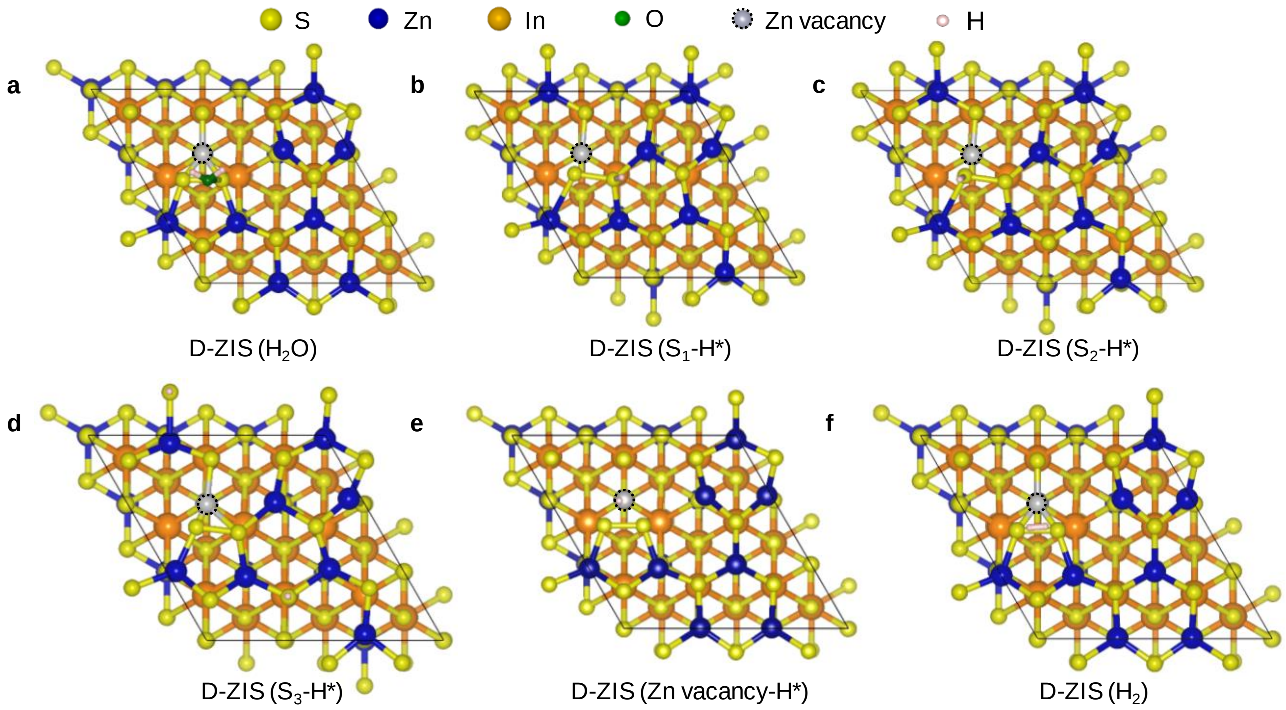

Supplementary Fig. 38. **DFT adsorption models of D-ZIS in the photocatalytic HER process**. **a** D-ZIS (H_2O); **b** D-ZIS (S_1-H*); **c** D-ZIS (S_2-H*); **d** D-ZIS (S_3-H*); **e** D-ZIS (Zn vacancy-H*); **f** D-ZIS (H_2). The models are displayed in top view.

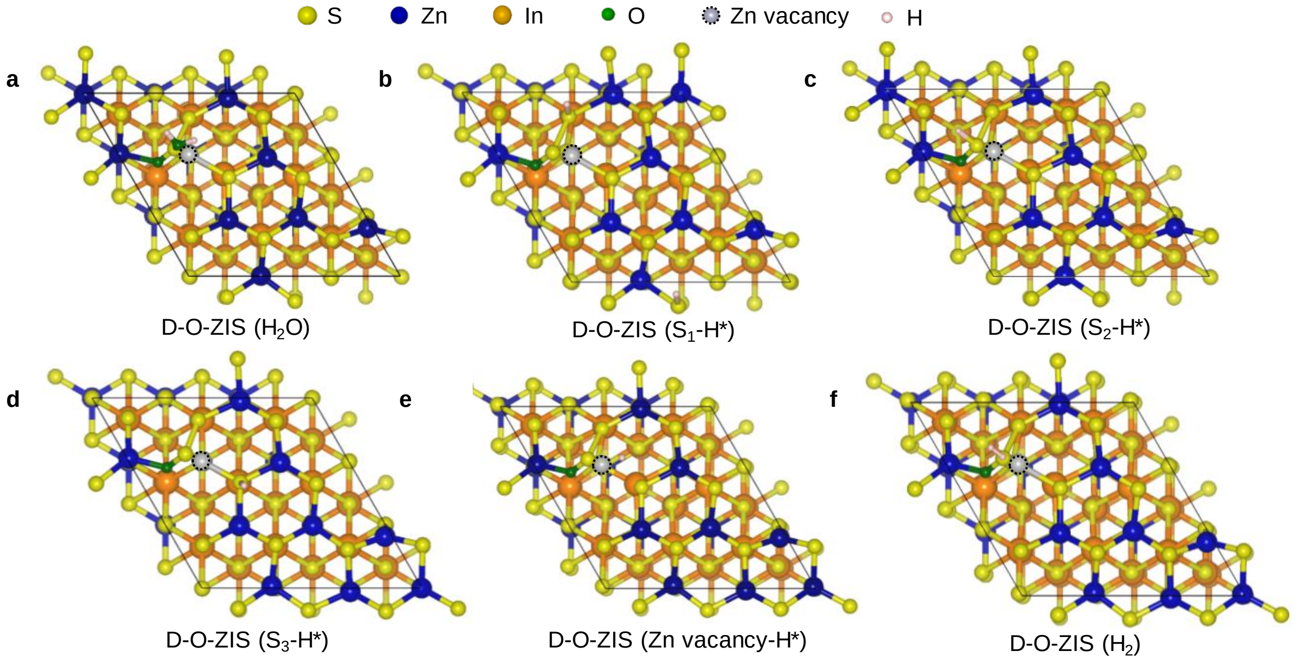

Supplementary Fig. 39. **DFT adsorption models of D-O-ZIS in the photocatalytic HER process**. **a** D-O-ZIS (H_2O); **b** D-O-ZIS (S_1-H*); **c** D-O-ZIS (S_2-H*); **d** D-O-ZIS (S_3-H*); **e** D-O-ZIS (Zn vacancy-H*); **f** D-O-ZIS (H_2). The models are displayed in top view.

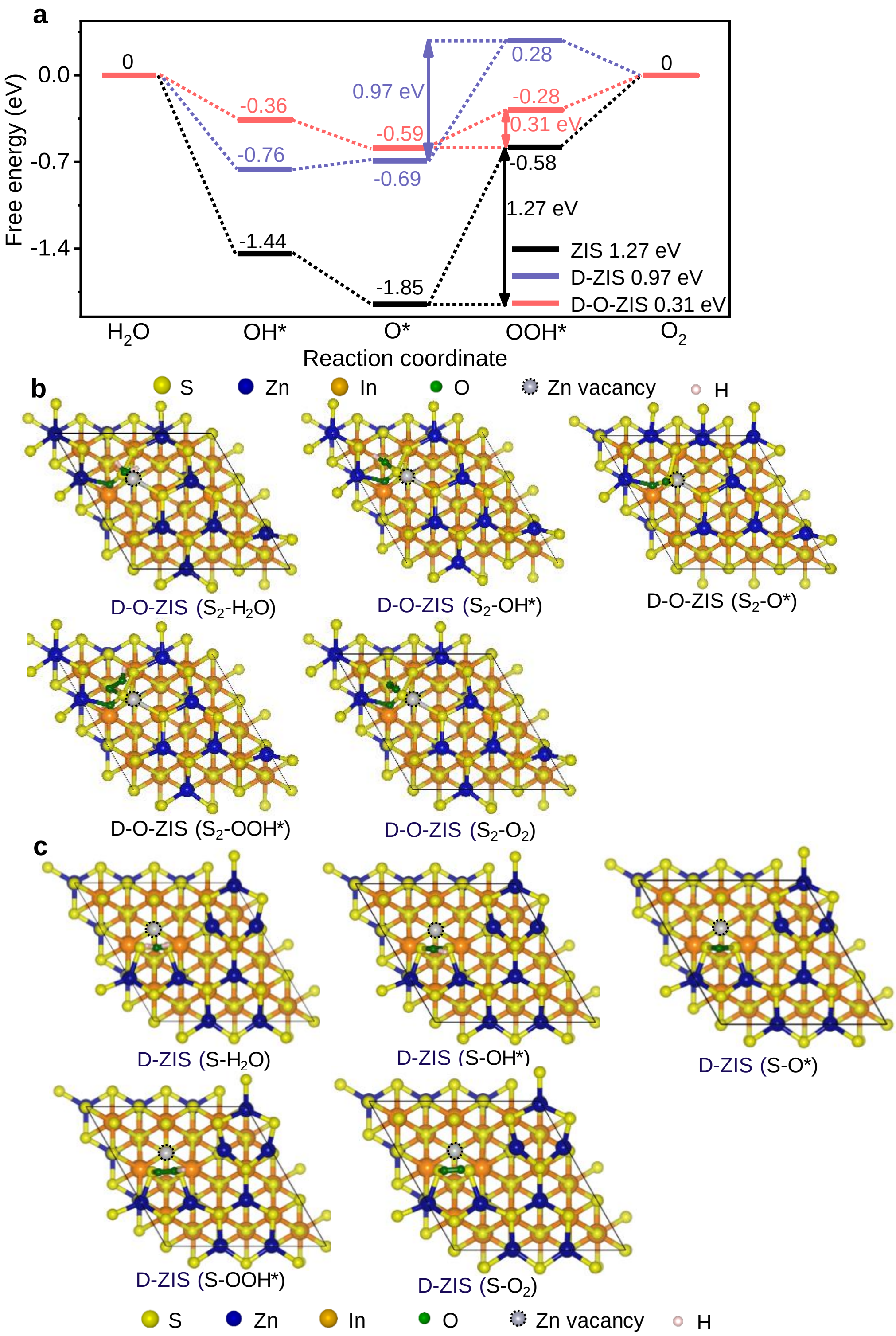

Supplementary Fig. 40. **DFT calculated for the photocatalytic OER process on S_2 sites of D-O-ZIS**. **a** DFT calculated free energy profile of OER process on ZIS, D-ZIS, and D-O-ZIS at pH=0 and U=1.23 V vs. SHE (where * represents the intermediate state); **b** The corresponding DFT models of H_2O, OH*, O*, OOH*, and O_2 intermediates adsorption on D-O-ZIS; **c** The corresponding DFT models of H_2O, OH*, O*, OOH*, and O_2 intermediates adsorption on D-ZIS. The corresponding DFT models of H_2O, OH*, O*, OOH*, and O_2 intermediates adsorption on ZIS seen in Supplementary Fig. 1k.

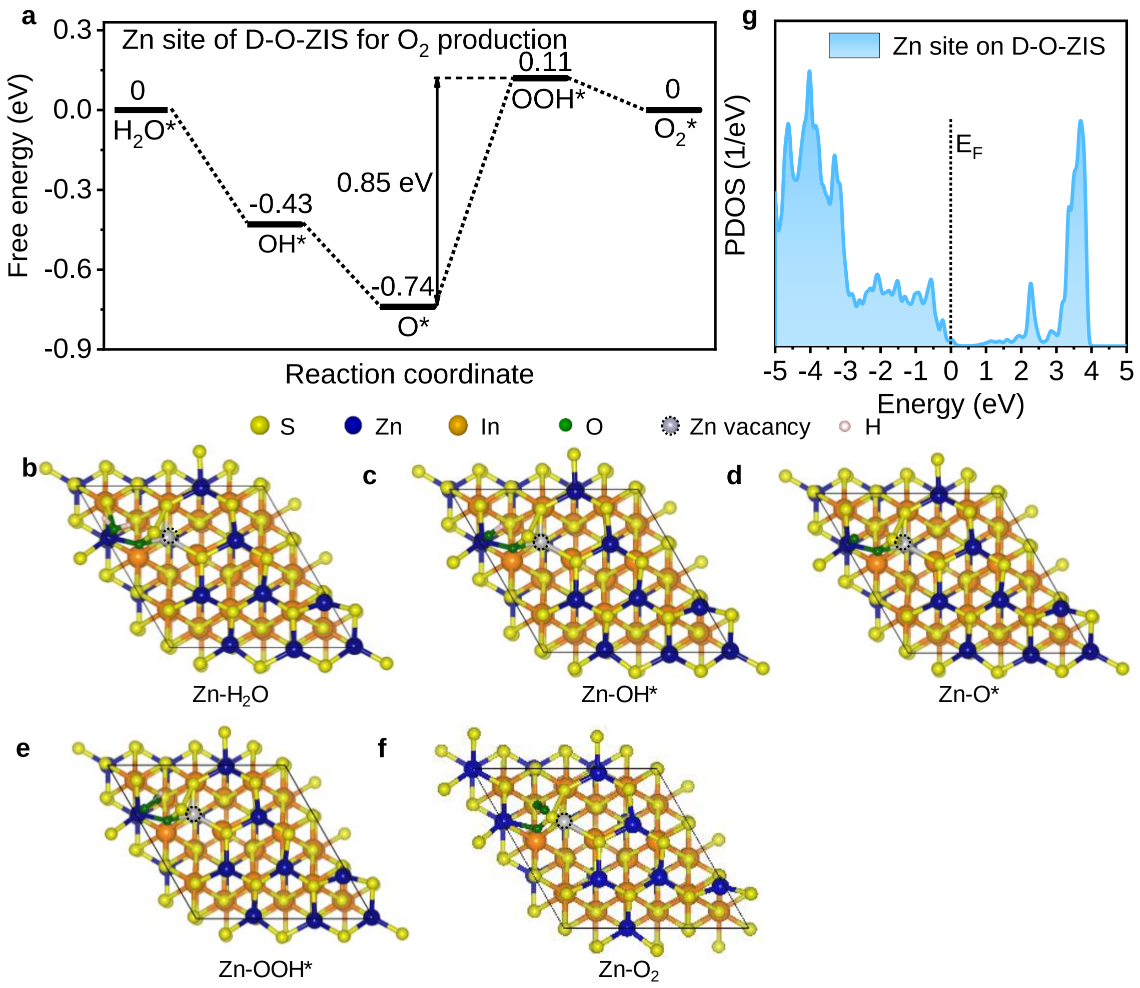

Supplementary Fig. 41. **DFT calculated for the photocatalytic OER process on Zn site of D-O-ZIS**. **a** DFT calculated free energy profile of OER process on Zn site of D-O-ZIS at pH=0 and U=1.23 V vs. SHE (where * represents the intermediate state); **b-f** The corresponding DFT models of H_2O, OH^*, O^*, OOH^*, and O_2 intermediates adsorption and activation on Zn site of D-O-ZIS; **g** The PDOS calculation of metal sites of Zn atoms in D-O-ZIS.

We investigate the oxygen evolution process on the outer surface Zn atoms as possible reacted sites of D-O-ZIS. Our findings suggest that Zn atoms are less active in oxygen production compared to the constructed active centers of S_1-S_2-O in D-O-ZIS. We observe a high free energy barrier of 0.85 eV for O^* adsorbed at the Zn site in the oxygen evolution process, which is much higher than that of adsorbed on the S_2 site (0.31 eV). Additionally, the minimum PDOS beyond the E_F of Zn atoms in D-O-ZIS indicates their electronic inactivity in water splitting reactions.

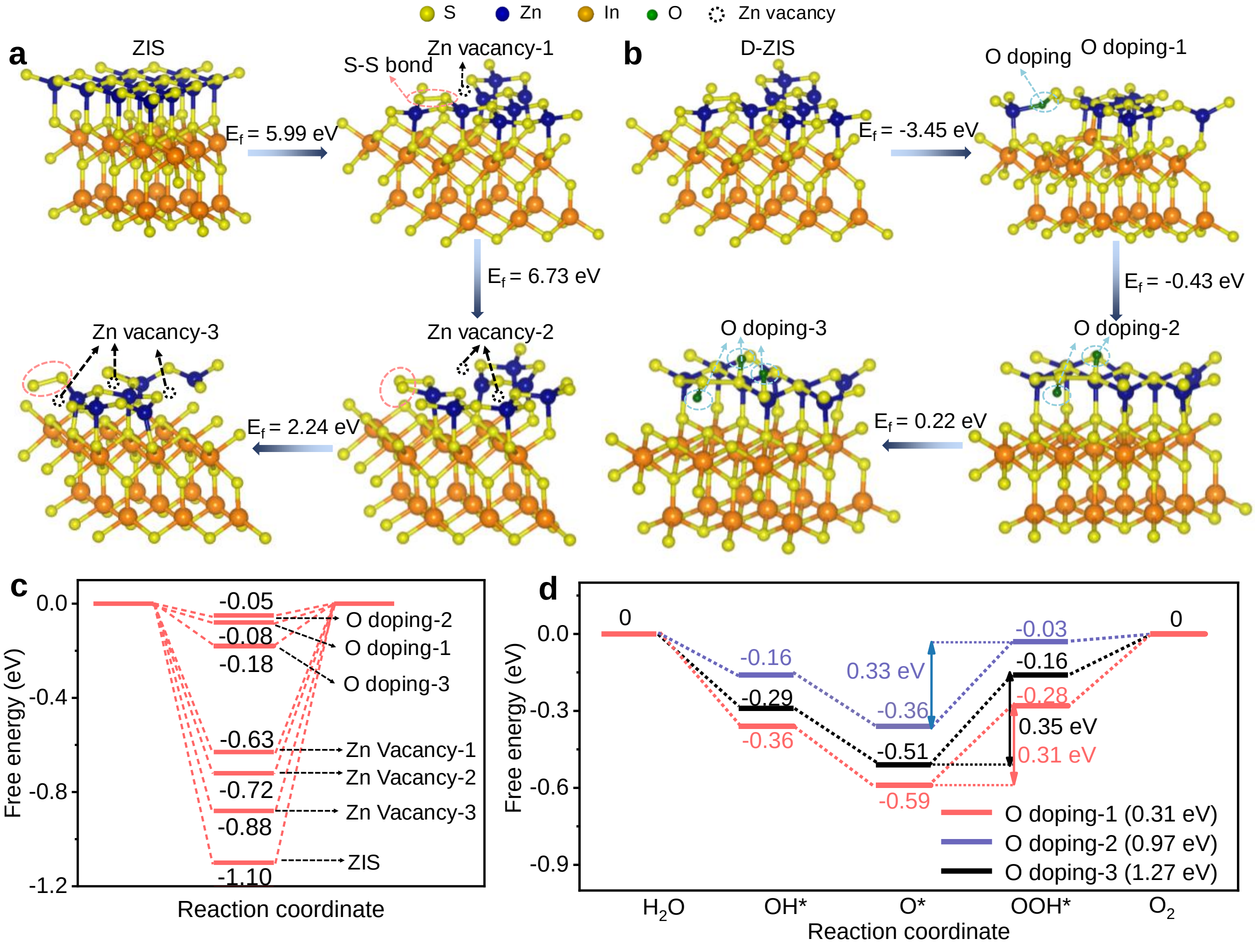

Supplementary Fig. 42. **DFT calculations on structures with varying Zn vacancy levels and O doping. a** Structural transition models and formation energies at different Zn vacancy concentrations; **b** Structural transition models and formation energies at different O doped concentrations; **c** The computed values of ΔG_{H*} at different sites in ZIS, Zn vacancy-1, 2, and 3, and O doping-1, 2, and 3; **d** DFT calculated free energy profile of OER process on O doping-1, 2, and 3 at pH=0 and U=1.23 V vs. SHE. (a single Zn vacancy: Zn vacancy-1; two Zn vacancies: Zn vacancy-2; three Zn vacancies: Zn vacancy-3), (a single oxygen dopant: O doping-1; two oxygen dopants: O doping-2; three oxygen dopants: O doping-3).

As shown in Supplementary Fig. 42a, the formation of a single vacancy (Zn vacancy-1) accompanied by the formation of an S-S bond requires an energy input of 5.99 eV. When two vacancies (Zn vacancy-2) are formed, a higher energy input of 6.73 eV is required. As the Zn vacancy concentration continues to increase to three (Zn vacancy-3), the energy input needed is 2.24 eV. Therefore, according to the principle of minimum energy, the ZIS structure tends to favor the formation of a stable structure of D-ZIS structure, which consists of a single vacancy and an accompanying S-S bond. However, as the Zn vacancy concentration further increases, the barrier for structure formation becomes higher, making it increasingly difficult to form. We conducted an investigation into various concentrations of O doping using calculations based on D-ZIS structure in Supplementary Fig. 42b.

When a single oxygen dopant (O doping-1) is introduced, the energy required for structure formation is -3.45 eV, indicating that oxygen doping is energetically favorable. As the concentration of O doping increases (with two oxygen dopants: O doping-2), the formation energy decreases to -0.43 eV. However, with further increases (O doping-3) in the O doping concentration, the energy barrier for structure formation becomes 0.23 eV, making it relatively challenging.

As shown in Supplementary Fig. 42c, d, it can be observed that the optimal hydrogen adsorption occurs at the S site when a single Zn vacancy concentration or two Zn vacancy concentrations are present, with corresponding values of -0.63 eV and -0.72 eV, respectively. As the Zn vacancy concentration continues to increase, the adsorption weakens, reaching -0.88 eV. For a single O doping concentration, the optimal hydrogen adsorption energy at the S site is -0.08 eV, while with two or three oxygen dopants, the hydrogen adsorption energy is -0.05 and -0.18 eV, respectively (Supplementary Fig. 42c). These results indicate that O doping enhances the electronic state of the S site, leading to favorable hydrogen adsorption. Supplementary Fig. 42d demonstrates that varying O doping concentrations have a minimal effect on the catalyst's oxygen evolution barriers. The oxygen evolution barriers for one, two, and three O doping concentrations are calculated to be 0.31 eV, 0.33 eV, and 0.35 eV, respectively.

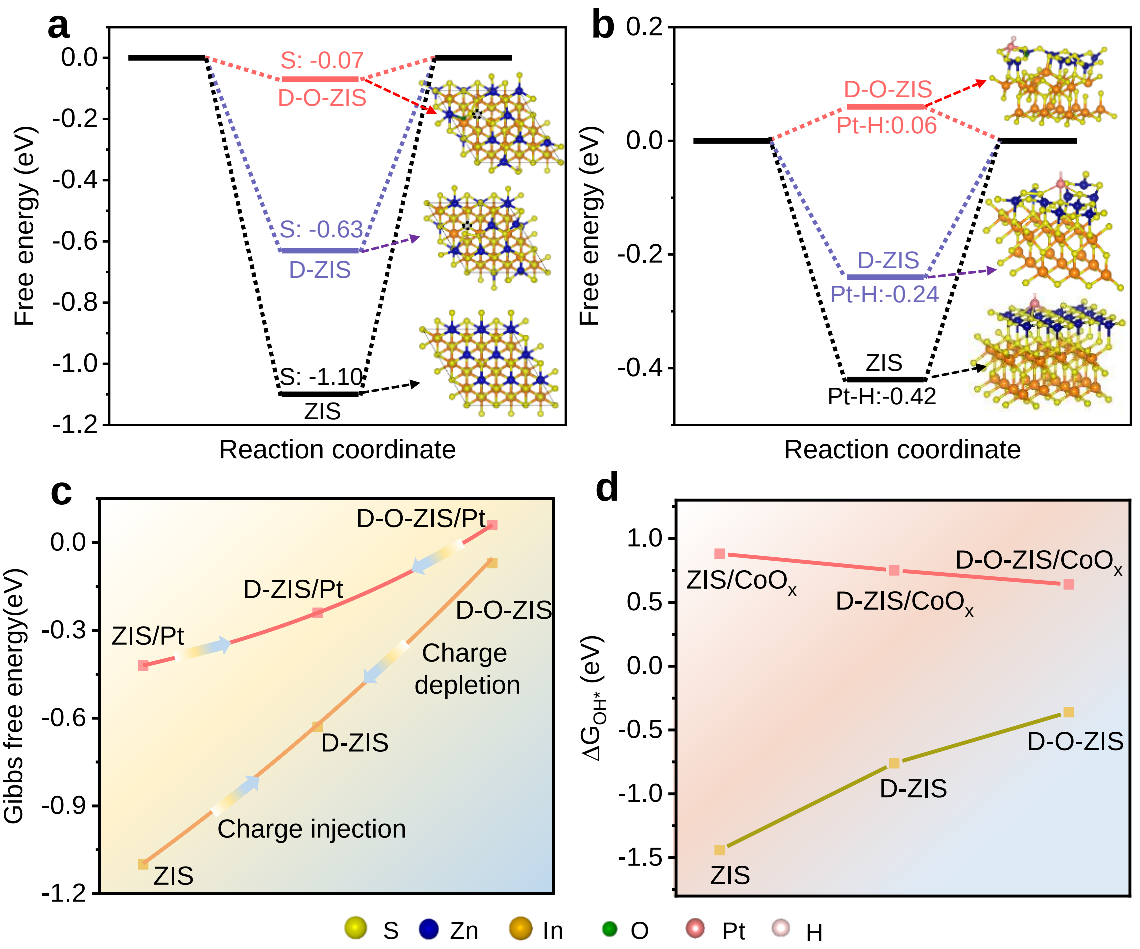

Supplementary Fig. 43. **The adsorption behavior of catalytic sites on photocatalysts. a** Gibbs free energies changes of HER process on S sites of ZIS, D-ZIS, and D-O-ZIS; **b** Gibbs free energies changes of HER process on Pt sites of ZIS/Pt, D-ZIS/Pt, and D-O-ZIS/Pt; **c** Electron injection/depletion effect of S sites and Pt sites on the ΔG_{H*}; **d** Adsorption free energies of OH* on ZIS, D-ZIS, and D-O-ZIS and on ZIS/CoO_x, D-ZIS/CoO_x, and D-O-ZIS/CoO_x at pH=0 and U=1.23 V vs. SHE.

Furthermore, we employed DFT calculations to investigate the adsorption behavior of crucial H and OH intermediates on photocatalysts. The hydrogen adsorption free energy (ΔG_{H*}) on ZIS, D-ZIS, and D-O-ZIS was firstly obtained, as shown in Supplementary Fig. 43a. The ZIS reveals the lowest adsorption energy of -1.10 eV. The low value demonstrates strong hydrogen adsorption, which indicates a relatively difficult desorption process. The ΔG_{H*} of D-ZIS is slight negative with a value of -0.63 eV, which means an improved adsorption process. Compared with D-ZIS, the ΔG_{H*} of D-O-ZIS is significantly improved from -0.63 to -0.07, demonstrating a moderate adsorption strength. We also examined the hydrogen adsorption on different Pt sites of ZIS/Pt, D-ZIS/Pt, and D-O-ZIS/Pt, as depicted in Supplementary Fig. 43b. The catalyst and Pt form a Schottky structure, and there is the largest Fermi level difference between D-O-ZIS and Pt, and the calculated D-O-ZIS/Pt has the optimal

hydrogen adsorption of 0.06 eV with charge equilibrium (Supplementary Fig. 43c) [24, 25].

Accordingly, the theoretical simulations verify that the different OH* adsorption strength on catalytic sites, thus regulating the hydroxide adsorption processes (Supplementary Fig. 43d). The relationship indicates an optimal value of ΔG_{OH*} of D-O-ZIS, which should be slightly negative. The biased adsorption energy may hinder either the hydroxide desorption process, causing the site blocking effect. The electron transfer in D-O-ZIS, D-O-ZIS/Pt, and D-O-ZIS/CoO_x modifys the electronic states of photocatalyst surface, significantly optimizing the adsorption of key intermediates and endowing D-O-ZIS with highly active oxygen evolution reaction sites [24, 25]. Thus, the hydrogen/oxygen adsorption further confirms the presence of charges injection and depletion of the D-O-ZIS, thereby stimulating hydrogen and oxygen activities of D-O-ZIS.

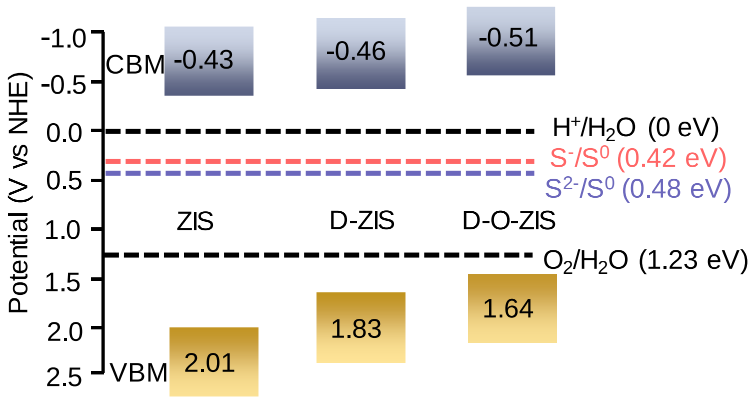

Supplementary Fig. 44. Schematic illustration of redox potentials of sulfur ions and band structure for samples.

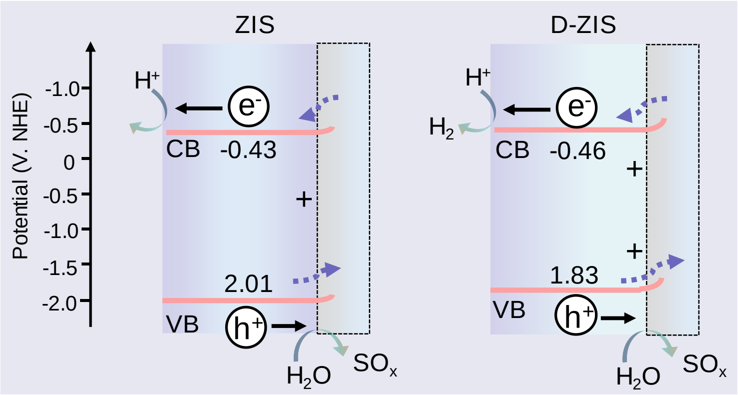

Supplementary Fig. 45. Photocatalytic water splitting mechanisms of ZIS and D-ZIS.

The catalytic mechanisms of ZIS and D-ZIS were illustrated in Supplementary Fig. 45. Firstly, ZIS and D-ZIS absorb incident photon to produce photogenerated charge carriers. The photogenerated electron-hole pairs are separated, and then transferred to the active sites to undergo a redox reaction. Due to the optimized energy bands, the CB of ZIS (-0.43 eV) and D-ZIS (-0.46 eV) is negative enough to produce H_2. Although the valence band (VB) energy is sufficiently positive to enable O_2 production in ZIS and D-ZIS, the inherent instability of sulfur atoms in these materials makes them prone to oxidation by holes. This leads to the generation of sulfate ions in the solution and, consequently, the degradation of the catalysts.

Supplementary References

[1] Wang, X., Wang, X., Huang, J., Li, S., Meng, A., Li, Z. Interfacial chemical bond and internal electric field modulated Z-scheme S_v-$ZnIn_2S_4/MoSe_2$ photocatalyst for efficient hydrogen evolution. *Nat. Commun.* ***12***, 4112-4123 (2021).

[2] Ravel, B., Newville, M. ATHENA, ARTEMIS, HEPHAESTUS: data analysis for X-ray absorption spectroscopy using IFEFFIT. *J. Synchrotron Radiat.* ***12***, 537-541 (2005).

[3] Zhao, D., Wang, Y., Dong, C., Huang, Y., Chen, J., Xue, F., Shen, S., Guo, L. Boron doped nitrogen-deficient carbon nitride-based Z-scheme heterostructures for photocatalytic overall water splitting. *Nat. Energy* ***6***, 388-397 (2021).

[4] Ekspong, J., Espino, E.G., Wagberg, T. Hydrogen evolution reaction activity of heterogeneous materials: a theoretical model. *J. Phys. Chem. C* ***124***, 20911-20921 (2020).

[5] Hu, Y., Pan, Y., Wang, Z., Lin, T., Gao, Y., Luo, B., Hu, H., Fan, F., Liu, G., Wang, L. Lattice distortion induced internal electric field in TiO_2 photoelectrode for efficient charge separation and transfer. *Nat. Commun.* ***11***, 2129-2139 (2020).

[6] Zhou, Y., Hao, W., Zhao, X., Zhou, J., Yu, H., Lin, B., Liu, Z., Pennycook, S.J., Li, S., Fan, H. Electronegativity-induced charge balancing to boost stability and activity of amorphous electrocatalysts. *Adv. Mater.* ***34***, 2100537-2100546 (2022).

[7] Suyatin, D.B., Jain, V., Nebolsin, V.A., Tragardh, J., Messing, M.E., Wagner, J.B., Persson, O., Timm, R., Mikkelsen, A., Maximov, I., Samuelson, L., Pettersson, H. Strong Schottky barrier reduction at Au-catalyst/GaAs nanowire interfaces by electric dipole formation and Fermi-level unpinning. *Nat. Commun.* ***5***, 3221-3229 (2020).

[8] Sun, G., Jiang, X., Li, X., Meng, L., Zhang, J., Qin, S., Kong, X., Li, J., Xin, J., Ma, W., Li, Y. High performance polymerized small molecule acceptor by synergistic optimization on π-bridge linker and side chain. *Nat. Commun.* ***13***, 5267-5278 (2022).

[9] Pean, E.V., Dimitrov, S., De Castro, C.S., Davies, M.L. Interpreting time-resolved photoluminescence of perovskite materials. *Phys. Chem. Chem. Phys.* ***22***, 28345-28358 (2020).

[10] Gu, J., Huang, Q., Yuan, Y., Ye, K., Wang, Z., Mai, W. In situ growth of a TiO_2 layer on a flexible Ti substrate targeting the interface recombination issue of $BiVO_4$ photoanodes for efficient solar water splitting. *J. Mater. Chem. A* **5**, 20195-20201 (2017).

[11] Yang, Q., Du, J., Li, J., Wu, Y., Zhou, Y., Yang, Y., Yang, D., He, H. Thermodynamic and kinetic influence of oxygen vacancies on the solar water oxidation reaction of α-Fe_2O_3 photoanodes. *ACS Appl. Mater. Interfaces* ***12***, 11625-11634 (2020).

[12] Kresse, G. Furthmüller, J. Efficient iterative schemes for ab initio total-energy calculations using a plane-wave basis set. *Phys. Rev. B* ***54***, 11169-11186 (1996).

[13] Kresse, G. Joubert, D. From ultrasoft pseudopotentials to the projector augmented-wave method. *Phys. Rev. B* ***59***, 1758-1775 (1999).

[14] He, Y., Rao, H., Song, K., Li, J., Yu, Y., Luo, Y., Li, C., Han, Y., Shi, Z., Feng, S. 3D hierarchical $ZnIn_2S_4$ nanosheets with rich Zn vacancies boosting photocatalytic CO_2 reduction. *Adv. Funct. Mater.* ***29***, 1905153-1905163 (2019).

[15] Tian, Z., Han, C., Zhao, Y., Dai, W., Lian, X., Wang, Y., Zheng, Y., Shi, Y., Pan, X., Huang, Z., Li, H., Chen, W. Efficient photocatalytic hydrogen peroxide generation coupled with selective benzylamine oxidation over defective ZrS_3 nanobelts. *Nat. Commun.* ***12***, 2039-2049 (2021).

[16] Zeng, K., Zheng, X., Li, C., Yan, J., Tian, J., Jin, C., Strasser, P., Yang, R. Recent advances in non noble bifunctional oxygen electrocatalysts toward large-scale production. *Adv. Funct. Mater.* ***30***, 2000503-2000526 (2020).

[17] Wang, Z., Li, C., Domen, K. Recent developments in heterogeneous photocatalysts for solar driven overall water splitting. *Chem. Soc. Rev.* ***48***, 2109-2125 (2019).

[18] Xu, W., Gao, W., Meng, L., Tian, W., Li, L. Incorporation of sulfate anions and sulfur vacancies in $ZnIn_2S_4$ photoanode for enhanced photoelectrochemical water splitting. *Adv. Energy Mater.* ***11***, 2101181-2101189 (2021).

[19] Pan, J., Fu, Y., Xiao, G., Niu, J., Cao, J., Wang, J., Zheng, Y., Li, C. Photocatalytic overall water splitting hydrogen evolution enhancement of ZnO nanoarrays/$LaCrO_3$ film heterojunction via HER/OER synergism of CoP/FTO. *J. Environ. Chem. Eng.* ***10***, 108587-108598 (2022).

[20] Shi, X., Dai, C., Wang, X., Hu, J., Zhang, J., Zheng, L., Mao, L., Zheng, H., Zhu, M. Protruding Pt single-sites on hexagonal $ZnIn_2S_4$ to accelerate photocatalytic hydrogen evolution. *Nat. Commun.* ***13***, 1287-1297 (2022).

[21] Wu, J., Liu, Z., Lin, X., Jiang, E., Zhang, S., Huo, P., Yan, Y., Zhou, P., Yan, Y. Breaking through water-splitting bottlenecks over carbon nitride with fluorination. *Nat. Commun.* ***13***, 6999-7007 (2022).

[22] Makula. P., Pacia, M., Macyk, W. How to correctly determine the band gap energy of modified semiconductor photocatalysts based on UV–vis spectra. *J. Phys. Chem. Lett.* ***9***, 6814-6817 (2018).

[23] Liu, F., Shi, R., Wang, Z., Weng, Y., Che, C., Chen, Y. Direct Z-scheme hetero-phase junction of black/red phosphorus for photocatalytic water splitting. *Angew. Chem. Int. Ed.* ***58***, 11791-11795 (2019).

[24] Han, T., Cao, X., Sun, K., Peng, Q., Ye, C., Huang, A., Cheong, W., Chen, Z., Lin, R., Zhao, D., Tan, X., Zhuang, Z., Chen, C., Wang, D., Li, Y. Anion exchange mediated internal electric field for boosting photogenerated carrier separation and utilization. *Nat. Commun.* ***12***, 4952-4963 (2021).

[25] Zhai, L., She, X., Zhuang, L., Li, Y., Ding, R., Guo, X., Zhang, Y., Zhu, Y., Xu, K., Fan, H., Lau, S. Modulating built-in electric field via variable oxygen affinity for robust hydrogen evolution reaction in Neutral Media. *Angew. Chem. Int. Ed.* ***61***, e202116057-e202116066 (2022).

[26] Chen, D., Lu, R., Yu, R., Dai, Y., Zhao, H., Wu, D., Wang, P., Zhu, J., Pu, Z., Chen, L., Yu, J., Mu, S. Work-function induced interfacial built-in electric fields in Os-$OsSe_2$ heterostructures for active acidic and alkaline hydrogen evolution. *Angew. Chem. Int. Ed.* ***61***, e202208642-e202208650 (2022).

[27] Zhou, P., Navid, I.A., Ma, Y., Xiao, Y., Wang, P., Ye, Z., Zhou, B., Sun, K., Mi, Z. Solar-to-hydrogen efficiency of more than 9% in photocatalytic water splitting. *Nature* ***613***, 66-70 (2023).

[28] Liu, M., Zhang, G., Liang, X., Pan, Z., Zheng, D., Wang, S., Yu, Z., Hou, Y., Wang, X. Rh/Cr_2O_3 and CoO_x cocatalysts for efficient photocatalytic water splitting by poly(triazine imide) crystals. *Angew. Chem. Int. Ed.* e202304694. DOI. 10.1002/anie.202304694.

[29] Wang, Q., Nakabayashi, M., Hisatomi, T., Sun, S., Akiyama, S., Wang, Z., Pan, Z., Xiao, X., Watanabe, T., Yamada, T., Shibata, N., Takata, T., Domen, K. Oxysulfide photocatalyst for visible-light-driven overall water splitting. *Nat. Mater.* ***18***, 827-832 (2019).

[30] Takata, T., Jiang, J., Sakata, Y., Nakabayashi, M., Shibata, N., Nandal, V., Seki, K., Hisatomi, T., Domen, K. Photocatalytic water splitting with a quantum efficiency of almost unity. *Nature* ***581***, 411-414 (2020).

[31] Song, X., Wei, G., Sun, J., Peng, C., Yin, J., Zhang, X., Jiang, Y., Fei, H. Overall photocatalytic water splitting by an organolead iodide crystalline material. *Nat. Catal.* ***3***, 1027-1033 (2020).

[32] Chen, X., Shi, R., Chen, Q., Zhang, Z., Jiang, Z., Jiang, W., Zhu, Y., Zhang, T. Three-dimensional porous g-C_3N_4 for highly efficient photocatalytic overall water splitting. *Nano Energy* ***59***, 644-650 (2019).

[33] Wei, S., Chang, S., Qain, J., Xu, X. Selective cocatalyst deposition on $ZnTiO_{3-x}N_y$ hollow nanospheres with efficient charge separation for solar driven overall water splitting. *Small* ***17***, 2100084-2100090 (2016).

[34] Lyu, Hao, Hisatomi, T., Goto, Y., Yoshida, M., Higashi, T., Katayama, M., Takata, T., Minegishi, T., Nishiyama, H., Yamada, T., Sakata, Y., Asakura, K., Domen, K. An Al-doped $SrTiO_3$ photocatalyst maintaining sunlight-driven overall water splitting activity for over 1000 h of constant illumination. *Chem. Sci.* ***10***, 3196-3201 (2018).

[35] Yang, Y., Chu, X., Zhang, H., Zhang, R., Liu, Y., Zhang, F., Lu, M., Yang, Z., Lan, Y. Engineering β-ketoamine covalent organic frameworks for photocatalytic overall water splitting. *Nat. Commun.* ***14***, 593-603 (2023).

[36] Kong, D., Xie, J., Guo, Z., Yang, D., Tang, J. Stable complete water splitting by covalent triazine-based framework CTF-0. *Chemcatchem* ***12***, 2708-2712 (2020).

[37] Chen, K., Xiao, J., Vequizo, J.J.M., Hisatomi, T., Ma, Y., Nakabayashi, M., Takata, T., Yamakata, A., Shibata, N., Domen, K. Overall water splitting by a $SrTaO_2N$-based photocatalyst decorated with an Ir-promoted Ru-based cocatalyst. *J. Am. Chem. Soc.* ***145***, 3839-3843 (2023).

[38] Wolff, C.M., Frischmann, P.D., Schulze, M., Bohn, B.J., Wein, R., Livadas, P., Carlson, M.T., Jackel, F., Feldmann. J., Wurthner, F., Stolarczyk, J.K. All-in-one visible-light-driven water splitting by combining nanoparticulate and molecular co-catalysts on CdS nanorods. *Nat. Energy* ***3***, 862-869 (2018).

[39] Zhang, G., Lan, Z., Lin, L., Lin, S., Wang, X. Overall water splitting by Pt/g-C_3N_4 photocatalysts without using sacrificial agents. *Chem. Sci.* *7*, 3062-3066 (2016).

[40] Wang, Z., Inoue, Y., Hisatomi, T., Ishikawa, R., Wang, Q., Takata, T., Chen, S., Shibata, N., Ikuhara, Y., Domen, K. Overall water splitting by an Ta_3N_5 nanorod single crystals grown on the edges of $KTaO_3$ particles. *Nat. Catal.* ***1***, 756-763 (2018).

[41] Pan, R., Hu, M., Liu, J., Li, D., Wan, X., Wang, H., Li, Y., Zhang, X., Wang, X., Jiang, J., Zhang, J. Two-dimensional all-in-one sulfide monolayers driving photocatalytic overall water splitting.

Nano Lett. ***21***, 6228-6236 (2021).

[42] Wang, M., Wang, J., Cheng, C., Zou, C., Zhang, R., Xie, Y., Guo, Z., Tang, C., Dong, C., Chen, Y., Du, X. A hydrogen-deficient nickel-cobalt double hydroxide for photocatalytic overall water splitting. *Angew. Chem. Int. Ed.* ***132***, 11510-11515 (2020).

[43] Wang, L., Liu, J., Wang, H., Cheng, H., Wu, X., Zhang, Q., Xu, H. Forming electron traps deactivates self-assembled crystalline organic nanosheets toward photocatalytic overall water splitting. *Sci. Bull.* ***66***, 265-274 (2021).

[44] Liu, X., Zhang, J., Xu, J., Li, Y., Du, Y., Jiang, Y., Lin, K. Hydroxyl-modified $Nb_4C_3T_x$ Mxene@$ZnIn_2S_4$ sandwich structure for photocatalytic overall water splitting. *J. Colloid Interface Sci.* ***633,*** 992-1001 (2023).

[45] Ding, Y., Wei, D., He, R., Yuan, R., Xie, T., Li, Z. Rational design of Z-scheme PtS-$ZnIn_2S_4$/WO_3-MnO_2 for overall photocatalytic water splitting under visible light. *Appl. Catal. B: Environ.* ***258,*** 117948-117956 (2019).

[46] Cai, X., Zeng, Z., Liu, Y., Li, Z., Gu, X., Zhao, Y., Mao, L., Zhang, J. Visible-light-driven water splitting by yolk-shelled $ZnIn_2S_4$ based heterostructure without noble-metal cocatalyst and sacrificial agent. *Appl. Catal. B: Environ.* ***297,*** 120391-120400 (2021).

[47] Zuo, G., Wang, Y., Teo, W., Xian, Q., Zhao, Y. Direct Z-scheme TiO_2-$ZnIn_2S_4$ nanoflowers for cocatalyst free photocatalytic water splitting. *Appl. Catal. B: Environ.* ***291,*** 120126-120134 (2021).

[48] Wan, S., Ou, M., Zhong, Q., Zhang, S., Song, F. Construction of Z-scheme photocatalytic systems using $ZnIn_2S_4$, CoO_x loaded Bi_2MoO_6 and reduced graphene oxide electron mediator and its efficient nonsacrificial water splitting under visible light. *Chem. Eng. J.* ***325,*** 690-699 (2017).

[49] Yang, G., Ding, H., Chen, D., Feng, J., Hao, Q., Zhu, Y. Construction of urchin-like $ZnIn_2S_4$-Au-TiO_2 heterostructure with enhance activity for photocatalytic hydrogen evolution. *Appl. Catal. B: Environ.* ***234,*** 260-267 (2018).

[50] Du, X., Zhao, T., Xiu, Z., Xing, Z., Li, Z., Pan, K., Yang, S., Zhou, W. $BiVO_4$@$ZnIn_2S_4$/Ti_3C_2 MXene quantum dots assembly all-solid-state direct Z-Scheme photocatalysts for efficient visible-light-driven overall water splitting. *Appl. Mater. Today* ***20***, 100719-100730 (2020).

[51] Yang, Y., Sun, Z., Liu, C., Wang, J., Qiu, M., Yan, G., Zhang, K. Boosting photocatalytic overall

water splitting on direct Z-Scheme $BiOBr/ZnIn_2S_4$ heterostructure by atomic-level interfacial charge transport modulation. *ACS Appl. Energy Mater.* ***5***, 15559-15565 (2022).

[52] Zhang, J., Zhang, Y., Li, L., Yan, W., Wang, H., Mao, W., Cui, Y., Li, Y., Zhu, X. Synergizing the internal electric field and ferroelectric polarization of the $BiFeO_3/ZnIn_2S_4$ Zscheme heterojunction for photocatalytic overall water splitting. *J. Mater. Chem. A* ***11,*** 434-447 (2023).

[53] Zuo, G., Ma, S., Yin, Z., Chen, W., Wang, Y., Ji, Q., Xian, Q., Yang, S., He, H. Z-Scheme modulated charge transfer on $InVO_4@ZnIn_2S_4$ for durable overall water splitting. *Small* ***19***, 2207031-2207040 (2023).

[54] Wang, Y., Huang, W., Guo, S., Xin, X, Zhang, Y., Guo, P., Tang, S., Li, X. Sulfur-deficient $ZnIn_2S_4$/Oxygen-deficient WO_3 hybrids with carbon layer bridges as a novel photothermal/photocatalytic integrated system for Z-Scheme overall water splitting. *Adv. Energy Mater.* ***11***, 2102452-2102461 (2021).

[55] Tran, P.D., Tran, T.V., Orio, M., Torelli, S., Truong, Q.D., Nayuki, K., Sasaki, Y., Chiam, S.Y., Honma, I., Barber, J., Artero, V. Coordination polymer structure and revisited hydrogen evolution catalytic mechanism for amorphous molybdenum sulfide. *Nat. Mater.* ***15***, 640-648 (2016).

[56] Zhang, Y., Miao, N., Xin, X., Wang, Y., Zhu, J., Guo, P., Wang, J., Sobrido, A.J., Titirici, M.M., Li, X. Boosting the photocatalytic performance via defect-dependent interfacial interactions from electrostatic adsorption to chemical bridging. *Nano Energy* ***104***, 107865-107875 (2022).
